# Supplementary material for: Multiscale metabolic engineering in biological lignin valorization
Source: Innovation (Camb). 2025 Jun 13;6(11):100993. doi: 10.1016/j.xinn.2025.100993 (PMC12628177; doi:10.1016/j.xinn.2025.100993)
Supplement: Document S1. Tables S1–S4 [file mmc1.pdf]

The Innovation, Volume *6*

Supplemental Information

Multiscale metabolic engineering in biological lignin valorization

Ruo-Ying Liu, Bing-Zhi Li, Ying-Jin Yuan, and Zhi-Hua Liu

Table S1 Lignocellulose fractionation and lignin depolymerization yield aromatic derivatives suitable for microbial metabolism

Strategies	Lignocellulose source or lignin type	System specifications or catalysts	Reaction temperature (°C)	Reaction time	Cleavage of linkages	Aromatic derivatives	Refs
Alkaline pretreatment	Sugarcane	NaOH (9% ww)	120	60 h	*β*-*O*-4	Phenol, guaiacol, 4-methylphenol, 4-methyl guaiacol, vanillin	S1
	Corn stover	50% Ethanol with 1% NaOH	120	80 h	*β*-*O*-4 and *β*-5	4-Hydroxybenzaldehyde, coumarone, *p*-coumaric acid, ferulic acid	S2
Acid pretreatment	Poplar chips	0.05 % (w/w) sulfuric acid	160-280	2-10 min	*β*-*O*-4	Vanillin, benzaldehyde, hydroxybenzaldehyde, 2-methoxy-4-vinylphenol, 2,6-dimethoxy-phenol, 4-hydroxy-3,5-dimethoxy-benzaldehyde, benzoic acids.	S3
	Alkali lignin	1.5 M hydrochloric acid with microwave heating	100	1 h	*β*-*O*-4	Phenol, Catechol, Isoeugenol, Eugenol, Vanillin, 2,6-dimethoxyphenol	S4
Extractive-ammonia pretreatment	Corn stover	Ammonia	120	30 min	Ester bonds	Low molecular weight lignin	S5
Organosolv pretreatment	Sorghum stem	1.90% NaOH with 70% ethanol	69.8	1 h	Ester bonds	*p*-Coumaric acid, ferulic acid	S6
	Rice straw	Ethanol/water mixture of 60.5% (w/w) ethanol concentration	30	1 h	Ester bonds	Tricin, vanillin, ferulic acid and *p*-coumaric acid	S7
Catalytic oxidative depolymerization	Aspen lignin	4-acetamido-TEMPO-initiated oxidation	Oxidation at 65°C, depolymerization at 110°C	Oxidation for 48 h	C-O bond	4-hydroxybenzoic acid, vanillin, vanillic acid, syringaldehyde, syringic acid	S8

	β-*O*-4 Linkages lignin model compounds	Heterogeneous cobalt nanoparticles as catalyst with dioxygen as the oxidant	150	24 h	C−C bond	Corresponding aromatic esters	S9
Catalytic reductive depolymerization	Poplar	Cobalt monatomic catalyst (CoSAs-N@LC)	160	1 h	C_{β}-C_{γ}, *β*-*O*-4	Methylparaben, *β*-hydroxypropiovanillone and acetovanillone	S10
	Soda lignin	Bimetallic $Ru_{0.6}Ni_{0.4}$ catalyst	350	40 min	*β*-*O*-4, *β*-5 and *β*-*β*	Guaiacol, 4-ethylphenol, 4-methylguaiacol, syringol	S11
Biological depolymerization	Lignin model compounds containing *β*-ether bonds	Versatile peroxidase from *B. adusta*	25	/	*β*-*O*-4	Guaiacol	S12
	Model dimeric lignin	Lignin peroxidase from *P. chrysosporium*	25	/	*β*-*O*-4	Guaiacol	S13
	Alkali lignin	DyP-type peroxidase from *Bacillus sp. Strain* BL5	35	20 min	*α*, *β* and *β*-ether bonds	Vanillin, 2-methyoxyphenol, *p*-hydroxybenzoic acid	S14
	Kraft lignin	Laccase from *Bacillus* sp. PCH94	50	Overnight	C−C bond	Ferulic acid, acetovanillone, dimers, trimer	S15
	Industry alkaline lignin	Laccase and manganese peroxidase from *L. betulina* and *T. versicolor*	30	168-192 h	Side-chain cleavage	4-Hydroxybenzoic acid, benzoic acid, phenylacetic acid	S16
	Alkaline lignin	Laccase from *Bacillus ligniniphilus* L1	37	24 h	*β*-*O*-4, *β*-5, *β*-*β,* 4-*O*-5, 5-5	Vanillyl alcohol, guaiacol, cetosyringenin	S17

	Corn stalk	Laccase from *Bacillus ligniniphilus* L1	37	24 h	*β*-*O*-4, *β*-5, *β*-*β,* 4-*O*-5, 5-5	Coniferyl aldehyde, syringaldehyde, 4-hydroxyphenylacetic acid, benzoic acid	S17
	5-5' Linkages lignin model compounds	Versatile peroxidase from *Physisporinus vitreus*	30	48 h	5-5'	Vanillic alcohol, vanillic aldehyde, and vanillic acid	S18
Combinatorial fractionation	Corn stover	Combinatorial chemical and mixed-enzyme treatments	/	/	*β*-*O*-4 and *β*-5	*p*-Coumaric acid, ferulic acid	S19

Table S2 Key enzymes and regulatory strategies in biological lignin valorization

Catalysis reaction	Enzymes	Gene origin	Classification	Coenzymes	Substrates	Products	Regulatory strategies	Regulation-related enzymes	Refs
O-demethylation	VanAB	*P. putida* KT2440	Rieske	FMN, NAD(P)H	Vanillic acid	Protocatechuic acid	Formaldehyde detoxification	HPS, PHI	S20
	VanAB	*Pseudomonas* sp. HR199	Rieske	FMN, NAD(P)H	Vanillic acid	Protocatechuic acid	NADH regeneration Formaldehyde detoxification	FADH, FDH	S21
	LigXaXcXd	*Sphingobium* sp. SYK-6	Rieske	FAD, NADH	5,5′-Dehydrovanillic acid	2,2′,3-Trihydroxy-3′-methoxy-5,5′-dicarboxybiphenyl	/	/	S22
	DesA	*Sphingobium* sp. SYK-6	THF-dependent demethylase	THF	Syringic acid	3-O-Methylgallic acid	THF regeneration	MetF, LigH	S23
	LigM	*Sphingobium* sp. SYK-6	THF-dependent demethylase	THF	Vanillic acid; 3-*O*-Methylgallic acid	Protocatechuic acid; Gallic acid	THF regeneration	MetF, LigH	S23
	GcoAB	*Amycolatopsis* sp. ATCC 39116	Cytochrome P450 enzyme	FAD, NADH	Guaiacol; Vanillin	Catechol; Protocatechualdehyde;	/	/	S24
Hydroxylation	PobA	*P. putida* KT2440	One-component FMO	FAD, NADPH	4-Hydroxybenzoic acid	Protocatechuic acid	Increasing cofactor availability	PraI	S25
	HpaBC	*E. coli*	Two-	FAD,	Tyrosine; Tyrosol	Levodopa;	$FADH_2$	LAAD	S26

			component FMO	NADH		Hydroxytyrosol	regeneration		
	HpaBC	HpaB from *P. aeruginosa* HpaC from *S. enterica*	Two-component FMO	FAD, NADH	*p*-Coumaric acid; 4-Hydroxybenzaldehyde;	Caffeic acid; Protocatechuic aldehyde	Enzyme fusion	/	S27
Decarboxylation	GS_120062	*G. subvermispora*	Oxidative decarboxylase	NAD(P)H	4-Hydroxybenzoic acid; Protocatechuic acid	Hydroquinone; 1,2,4-Benzenetriol	/	/	S28
	GS_90429	*G. subvermispora*	Oxidative decarboxylase	NAD(P)H	4-Hydroxybenzoic acid; Protocatechuic acid	Hydroquinone; 1,2,4-Benzenetriol	/	/	S28
	TV_32834	*T. versicolor*	Oxidative decarboxylase	NAD(P)H	Protocatechuic acid	1,2,4-Benzenetriol	/	/	S28
	FDC	*S. cerevisiae*	UbiD-type	PrFMN	*p*-Coumaric acid; Ferulic acid; Cinnamic acid	4-Vinylphenol; 4-Vinylguaiacol; Styrene	Co-expression of related proteins	PAD	S29
	AroY	*E. cloacae*	UbiD-type	PrFMN	Protocatechuic acid	Catechol	Co-expression of related proteins	EcdB; EcdD	S30
	LigW	*Sphingobium* sp. SYK-6	Amidohydrolase superfamily	Mn^{2+}	5-Carboxyvanillic acid	Vanillic acid	/	/	S31

Table S3 Examples of regulation of metabolic networks for valorizing lignin into high-value products

Classification	Products	Host strains	Substrates	Functional modules for regulation	Engineering strategies	Other strategies	Titers	Refs
Aromatic platform chemicals	Vanillin	*E. coli*	Ferulic acid	Vanillin synthesis module and CoA-regeneration module	Heterologous expression of *fcs* and *ech* from *Amycolatopsis* sp. HR104<br>Overexpression of *gltA*<br>Knockout of *icdA*	Application of XAD-2 resin for adsorption of toxic products	5.14 g/L	S32
	Vanillin	*S. cerevisiae*	Real hydrolysate of corn stover	Vanillin synthesis module, xylose metabolism module and tyrosine overproduction module	Knockout of *ADH*6, *ADH*7, *BDH*1, *BDH*2, *GRE*2, *HFD*1, *ARO*10, *PDC*5, *PHA*2, *FDC*1 and *PAD*1<br>Heterologous expression of *Pc*4CL, *Pp*Ech, *Pa*HpaB, *Se*HpaC, *At*COMT, *Nt*COMT, *Fj*TAL, *Ec*AROL,<br>Overexpression of *SAH1*, *MET6*, $MET13_{atsc}$, $Aro3^{K222L}$, $Aro4^{K229L}$, $Aro7^{G141S}$, XYL1, mXYL2, XKS1	Combined depolymerization strategies of lignin; Protein fusion expression	1.97 mM	S27
	Vanillin	*E. coli*	Ferulic acid	Product synthesis module and cell growth module	Combinatorial expression of PP3359 and *Pp*FCS	Two-Layer gene circuit	852 mg/L	S33
	p-Hydroxybenzaldehyde	*E. coli*	*p*-Coumaric acid	Product synthesis module and cell growth module	Combinatorial expression of PP3359 and *Pp*FCS	Two-Layer gene circuit	393 mg/L	S33
	Protocatechuic acid	*S. cerevisia*	Alkaline pretreatment	Protocatechuic acid synthesis	Knockout of *ADH6*, *ADH7*, *BDH2*, *FDC*, *MHT1*, *SAM4*	Fermentation optimization	810 mg/L	S34

		e	liquor	module and THF-regeneration module	Heterologous expression of *Pc*4CL, *Pp*ECH, *Pp*VDH, *Pp*PobA, *Pd*VanA, *Pd*VanB, *Sp*LigM, *Rd*Metf1<br>Overexpression of MET6			
	Protocatechuic acid	*P. putida* KT2440	*p*-Coumaric acid and ferulic acid	Protocatechuic acid synthesis module and aromatic substrates transport module	Knockout of PcaGH<br>Combinatorial expression of PobA, Hcnk, *Bs*PadR and *Ac*VanAB	Auto-regulatory system; Promoter engineering; Fed-batch fermentation	12.7 g/L	S35
	Vanillic acid	*E. coli*	Ferulic acid	Product synthesis module and cell growth module	Combinatorial expression of PP3359 and *Pp*FCS	Two-Layer gene circuit	880 mg/L	S33
	Gallic acid	*Rhodococcus opacus* PD630	Base-depolymerized ammonia fiber explosion lignin	Gallic acid synthesis module and THF-regeneration module	Knockout of catechol 2,3-dioxygenase gene and protocatechuate 3,4-dioxygenase gene<br>Overexpression expression of PobA$^{Tyr386Phe/Thr295Ala}$, DesV, DesA,<br>Heterologous expression of *Ss*LigM, *Ss*MetF, *Ss*LigH	Protein engineering<br>Combined depolymerization strategies of lignin	2.469 mM	S23
	Vanillylamine	*P. putida* KT2440	Ferulic acid	Vanillylamine synthesis module and L-alanine regeneration module	Knockout of PcaGH, AreA, VDH, ALD and BDH<br>Overexpression of FCS, ECH<br>Heterologous expression of *Cv*ATA, *Bs*AlaDH	Whole-cell bioconversion	0.473 mM	S36
	Vanillylamine	*E. coli*	Ferulic acid	Vanillylamine	Heterologous expression of *Pp*FCS,	Increasing the ratio	19.22	S37

				synthesis module and L-alanine regeneration	*Pp*ECH, *Cv*TA, *Bs*AlaDH	of the NH4Cl donor	mM	
	Vanillylamine	*E. coli*	Vanillic acid	Vanillylamine synthesis module and L-alanine regeneration	Heterologous expression of *Sr*CAR, *Ni*PPTase, *Cv*TA, *Bs*AlaDH	Increasing the ratio of the NH4Cl donor	18.9 mM	S37
	Catechol	*E. coli*	Vanillin	Catechol synthesis module and aromatic substrates transport module	Combinatorial expression of *Rp*CouP, *Sp*LigV, *Sp*LigM, *Kp*AroY	Autoregulatory system	Approx. 8 mg/L	S38
Ring-opening products	Polyhydroxyalkanoates (PHAs)	*P. putida* KT2440	*p*-Coumaric acid	PHA accumulation module, fatty acid synthesis module, and *β*-oxidation module	Knockout of PhaZ, FadA, FadB Overexpression of PhaG, AlkK, PhaC	Fed-batch fermentation	953 mg/L	S39
		P. putida KT2440	Soluble lignin-rich stream	PHA accumulation module, fatty acid synthesis module, and *β*-oxidation module	Knockout of PhaZ, FadA, FadB Overexpression of PhaG, AlkK, PhaC	Combined depolymerization strategies of lignin	116 mg/L	S39
		P. putida KT2440	*p*-Coumaric acid	PHA accumulation	Knockout of PhaZ,AldB, FadA, FadB Overexpression of PhaG, PhaC, PhaJ4	Fed-batch fermentation	2.46 g/L	S40

			module and fatty acid *β*-oxidation				
	P. putida KT2440	Real lignin hydrolysate	PHA accumulation module and fatty acid *β*-oxidation module	Knockout of PhaZ,AldB, FadA, FadB<br>Overexpression of PhaG, PhaC, PhaJ4	Fed-batch fermentation	0.93 g/L	[S40]
	P. putida A514	Lignin-enriched biorefinery residue	Lignin enzymatic depolymerization module, PHA accumulation module and fatty acid *β*-oxidation module	Heterologous expression of *Am*DyP2<br>Overexpression of VanAB, PhaJ4, PhaC1	Multi-omics analysis	Over 160 mg/L	[S41]
Polyhydroxybutyrate (PHB)	*Ralstonia eutropha* H16	Alkaline pretreated liquor of *Pinus massoniana*	*O*-demethylation module, Aromatic aldehyde metabolism module, and the mitigation of by-product inhibition module	Laboratory-evolved strains with the *gabD1* gene replaced with *pcaQ*^{R145K} /P_{PCA}-*vanAB* and *fdhA* gene; the *iclA* gene replaced with *pcaQ*^{R145K}/P_{PCA}-*vdh*-*aldBI*-*aldBII* and the *iclB* gene replaced with *pcaQ*^{R145K}/P_{PCA}-*vanAB* and Ptac *fdhA* gene	Fed-batch fermentation, protein engineering, laboratory evolution and autoregulation	2.38 g/L	[S42]
cis-cis Muconic acid	*P. putida* KT2440	Real lignin hydrolysate	Muconic acid synthesis module, the S-type lignin utilization	Knockout of PcaHG,<br>Overexpression of VanAB, ZwfA<br>Heterologous expression of *Ec*AroY, EcdB	Fed-batch fermentation	9.6 mM	[S43]

			module, and the NADPH regeneration module				
	P. putida KT2440	Glycerol, *p*-coumaric acid, ferulic acid, and syringic acid	Muconic acid synthesis module, the S-type lignin utilization module, and the NADPH regeneration module	Knockout of PcaHG,<br>Overexpression of VanAB, ZwfA<br>Heterologous expression of *Ec*AroY, EcdB	Fed-batch fermentation	46.5 mM	S43
	P. putida KT2440	*p*-Coumaric acid	Muconic acid synthesis module and global carbon metabolism module	Knockout of CatRBC, Crc, PcaHG<br>Heterologous expression of AroY, EcdBD	High pH fed-batch fermentation	50 g/L	S44
	P. putida KT2440	High-pH lignin streams	Muconic acid synthesis module and global carbon metabolism module	Knockout of CatRBC, Crc, PcaHG<br>Heterologous expression of AroY, EcdBD	Fed-batch fermentation	3.7 g/L	S44
β-Ketoadipic acid	*P. putida* KT2440	*p*-Coumaric acid	*β*-Ketoadipic acid synthesis module and global carbon metabolism	Knockout of PobA, Crc, PcaHG<br>Overexpression of VanAB, PacHG<br>Heterologous expression of *Pa*PraI$_{JJ\text{-}1b}$	Fed-batch fermentation	44.5 g/L	S45

				module				
		P. putida KT2440	Alkaline pretreated liquor	*β*-Ketoadipic acid synthesis module and global carbon metabolism module	Knockout of PobA, Crc, PcaHG, PcaIJ, LvaE<br>Overexpression of VanAB, PacHG<br>Heterologous expression of *Pa*PraI$_{\text{JJ-1b}}$	Fed-batch fermentation	25 g/L	S45
	2-Pyrone-4,6-dicarboxylic acid	*P. putida* KT2440	Protocatechuic acid	2-Pyrone-4,6-dicarboxylic acid synthesis module and aromatic substrates transport module	Knockout of PcaG<br>Heterologous expression of *Sp*LigAB<br>Overexpression of PcaK, PobA	Resting cells	0.58 g/L/h	S46
Natural products	Naringenin	*E. coli*	*p*-Coumaric acid	Flavonoids synthesis module and Malonate assimilation module	Heterologous expression of *Rt*MatB, *Rt*MatC, 4CL, CHS, CHI	Shake flask	155 mg/L	S47
	Naringenin	*E. coli*	*p*-Coumaric acid	Flavonoids synthesis module and malonyl CoA overproduction module	Knockout of SdhA, AdhE, BrnQ, CitE<br>Heterologous expression of CHI, CHS, 4CL2, ACC, BPL	Developed a cipher of evolutionary design to predict gene targets	215 mg/L	S48
	Homoeriodictyol	*S. cerevisiae*	*p*-Coumaric acid	Homoeriodictyol synthesis module, CoA supply module and SAM	Heterologous expression of *Pc*4CL, *Ms*CHI, *Ph*CHS, *Md*ECR, *At*ATR1, *At*F3'H, *Os*ROMT<br>Overexpression of ACC1, MET6, SAH1,	Protein engineering	3.2 mM	S49

				regeneration module	ADO1, MET13, Mthfr, Knockout of CIT2, FDC1, Tsc13,			
	Kaempferol	*S. cerevisiae*	*p*-Coumaric acid and Glucose	Kaempferol synthesis module and Acetyl-CoA overproduction module	Heterologous expression of *Eb*4CL, *Eb*CHS, *Eb*CHI,*Eb*PAL, *Eb*C4H, *At*F3H, *Pd*FLS Overexpression of *ADH2*, *ALD6*, ACS^{SE}, *ACC1*	Fed-batch fermentation	66.29 mg/L	S50

Table S4 Genome-scale metabolic models of ligninolytic microbes

Strains	Models	Metabolites	Genes	Reactions	Sources of data	Reconstruction	Aromatic compound metabolic pathways/enzymes	Refs
Pseudomonas putida KT2440	*i*JN746	911	746 (14%) [a]	950	TIGR, KEGG, PSEUDOCYC, SYSTOMONAS	Constraint-based reconstruction	*β*-Ketoadipate pathway, homogentisate pathway, phenylacetate pathway, gallate pathways and toluene and xylene degradation pathway	[S51]
	*i*JP815	888	815 (15%) [a]	877	The *Pseudomonas* Genome Database, NCBI, KEGG, BRENDA	Constraint-based reconstruction	Benzoate degradation via hydroxylation and benzoate degradation via CoA ligation	[S52]
	PpuMBEL1071	1044	900 (16.6%) [a]	1071	Genome annotation, CMR, KEGG, UMBBD, the *Pseudomonas* Genome Database, BioSilico, TransportDB	Constraint-based reconstruction	*β*-Ketoadipate pathway and homogentisate pathway	[S53]
	*i*JP962	992	962 (17%) [a]	1070	*Pseudomonas* Genome Database V2	Metabolic network reconciliation	Naphthalene degradation pathway, anthracene degradation pathway, phenylalanine degradation pathway and benzoate degradation pathway	[S54]
	*i*EB1050	1122	1050 (19%) [a]	1256	RFAM, UniProtKB, COG, HAMAP, FIGfam	MicroScope automatic reconstruction	CatA/A1, CatB, CatC,CatR, PcaHG,PcaIJ,PcaBCD, PcaP, PcaR, PcaK ,PcaF, GalABCD,GalT, GalP, BenABCDE, BenK, BenR, Fcs,Ech,	[S55]

							Vdh,VanAB, PobA, CalAB	
	PpuQY1140	1104	1140 (21%) [a]	1171	NCBI, KEGG, MetaCyc	Pathway-Consensus approach	PP_2518, PP_2513, PP_2515, PP_2514, PP_3357, PP_3358, PP_3356, PP_5120	S56
	*i*JN1462	2155	1462 (27%) [a]	2929	Genome annotation	Iterative tri-dimensional expansion	PcaK, 3-oxoadipatesuccinyl-CoA transferase and 3-oxoadipyl-CoA thiolase	S57
Rhodococcus opacus PD630	iGR1773	1956	1773	3025	Genome annotation, transcript data from the National Center for Bioinformatics Sequence Read Archive, , BiGG and KEGG	CarveMe automatic reconstruction	Phenol degradation pathway	S58
Novosphingobium aromaticivorans	iNovo479	604	479	645	KEGG annotation data for *N. aromaticivorans* DSM 12444, Integrated Microbial Genomes Database,	Constraints based on S-type aromatic metabolism	Vanillic acid demethylation pathway, syringic acid demethylation pathway, guaiacylglycerol-β-guaiacyl ether degradation pathway	S59
Pluralibacter gergoviae	*i*LL1097	1771	/	1886	Genome sequence for *P. gergoviae*, GenBank, UniProt , BiGG, KEGG and MetaCyc	Model Seed automatic reconstruction	DyP,AdhAB, Cat, Dld, Gox, Gpx, MdlC, NuoE,NuoF,NuoG, Sod, TrxB, CatABC, BenABCD, PobA, PcaHG,PcaIJ,PcaBCD, HpaBCG	S60
Rhodosporidium toruloides	/	2051	1141	2398	OrthoMCL, BiGG	COBRApy was used for modeling of the	Long-chain acyl-CoA synthetase, enoyl-CoA hydratase/isomerase, peroxisomal dehydratase, 3-	S61

		reconstructed metabolic network	oxoacyl-(acyl-carrier protein) reductase, 3-oxoacyl CoA thiolase, alpha/beta hydrolase, monooxygenase, lactonase, carboxymuconolactone decarboxylase, 3-oxoacid CoA-transferase, acetyl-CoA acyltransferase, mitochondrial 2-oxodicarboxylate transporter, aspartate aminotransferase, vanillin dehydrogenase, aldehyde dehydrogenase, Phenylalanine/tyrosine ammonia-lyase, cytochrome P450

a: % of genes represented

References

S1. Martínez P. M., Punt A. M., Kabel M. A., et al. (2016). Deconstruction of lignin linked *p*-coumarates, ferulates and xylan by NaOH enhances the enzymatic conversion of glucan. *Bioresour. Technol.* **216**:44 – 51. DOI:10.1016/j.biortech.2016.05.040

S2. Liu Z.H., Olson M. L., Shinde S., et al. (2017). Synergistic maximization of the carbohydrate output and lignin processability by combinatorial pretreatment. *Green Chem.* **19**:4939 – 4955. DOI:10.1039/c7gc02057k

S3. Zhang L., Yan L., Wang Z., et al. (2015). Characterization of lignin derived from water-only and dilute acid flowthrough pretreatment of poplar wood at elevated temperatures. *Biotechnol. Biofuels* **8**:1 – 14. DOI:10.1186/s13068-015-0377-x

S4. Duan D., Ruan R., Wang Y., et al. (2018). Microwave-assisted acid pretreatment of alkali lignin: Effect on characteristics and pyrolysis behavior. *Bioresour. Technol.* **251**:57 – 62. DOI:10.1016/j.biortech.2017.12.022

S5. da Costa Sousa L., Foston M., Bokade V., et al. (2016). Isolation and characterization of new lignin streams derived from extractive-ammonia (EA) pretreatment. *Green Chem.* **18**:4205 – 4215. DOI:10.1039/C6GC00298F

S6. Li D., Long L. and Ding S. (2020). Alkaline organosolv pretreatment of different sorghum stem parts for enhancing the total reducing sugar yields and *p*-coumaric acid release. *Biotechnol. Biofuels* **13**:106. DOI:10.1186/s13068-020-01746-4

S7. Moniz P., Serralheiro C., Matos C. T., et al. (2018). Membrane separation and characterisation of lignin and its derived products obtained by a mild ethanol organosolv treatment of rice straw. *Process Biochem.* **65**:136 – 145. DOI:10.1016/j.procbio.2017.11.012

S8. Rahimi A., Ulbrich A., Coon J. J., et al. (2014). Formic-acid-induced depolymerization of oxidized lignin to aromatics. *Nature* **515**:249 – 252. DOI:10.1038/nature13867

S9. Luo H., Wang L., Shang S., et al. (2020). Cobalt nanoparticles-catalyzed widely applicable successive C-C bond cleavage in alcohols to access esters. *Angew. Chem., Int. Ed. Engl.* **59**:19268 – 19274. DOI:10.1002/anie.202008261

S10. Ge J., Wang G., Sui W., et al. (2023). Highly efficient metal-acid synergetic catalytic fractionation of lignocellulose under mild conditions over lignin-coordinated N-anchoring Co single-atom catalyst. *Chem. Eng. J.* **462**:142109. DOI:10.1016/j.cej.2023.142109

S11. Kim J.Y., Park S. Y., Choi I.G., et al. (2018). Evaluation of Ru_xNi_{1-x}/SBA-15 catalysts for depolymerization features of lignin macromolecule into monomeric phenols. *Chem. Eng. J.* **336**:640 – 648. DOI:10.1016/j.cej.2017.11.118

S12. Zeng J., Mills M. J., Simmons B. A., et al. (2017). Understanding factors controlling depolymerization and polymerization in catalytic degradation of *β*-ether linked model lignin compounds by versatile peroxidase. *Green Chem.* **19**:2145 – 2154. DOI:10.1039/C6GC03379B

S13. Pham L. T. M., Seo H., Kim K.J., et al. (2018). In silico-designed lignin peroxidase from *Phanerochaete chrysosporium* shows enhanced acid stability for depolymerization of lignin. *Biotechnol. Biofuels* **11**:1 – 13. DOI:10.1186/s13068-018-1324-4

S14. Khan S. I., Zada N. S., Sahinkaya M., et al. (2021). Cloning, expression and biochemical characterization of lignin-degrading DyP-type peroxidase from *Bacillus* sp. Strain BL5. *Enzyme Microb. Technol.* **151**:109917. DOI:10.1016/j.enzmictec.2021.109917

S15. Kumar V, Chandra D, Thakur V, et al. (2023). Depolymerization of lignin using laccase from

Bacillus sp. PCH94 for production of valuable chemicals: a sustainable approach for lignin valorization. *Int. J. Biol. Macromol.* **234**:123601. DOI:10.1016/j.ijbiomac.2023.123601

S16. Cui T., Yuan B., Guo H., et al. (2021). Enhanced lignin biodegradation by consortium of white rot fungi: microbial synergistic effects and product mapping. *Biotechnol. Biofuels* **14**:1 – 11. DOI:10.1186/s13068-021-02011-y

S17. Zhu D., Liang N., Zhang R., et al. (2020). Insight into depolymerization mechanism of bacterial laccase for lignin. *ACS Sustainable Chem. Eng.* **8**:12920 – 12933. DOI:10.1021/acssuschemeng.0c03457

S18. Kong W., Fu X., Wang L., et al. (2017). A novel and efficient fungal delignification strategy based on versatile peroxidase for lignocellulose bioconversion. *Biotechnol. Biofuels* **10**:218. DOI:10.1186/s13068-017-0906-x

S19. Liu Z.H., Shinde S., Xie S., et al. (2019). Cooperative valorization of lignin and residual sugar to polyhydroxyalkanoate (PHA) for enhanced yield and carbon utilization in biorefineries. *Sustainable Energy Fuels* **3**:2024 – 2037. DOI:10.1039/c9se00021f

S20. Nguyen L. T., Tran M. H. and Lee E. Y. (2021). Co-upgrading of ethanol-assisted depolymerized lignin: A new biological lignin valorization approach for the production of protocatechuic acid and polyhydroxyalkanoic acid. *Bioresour. Technol.* **338**:125563. DOI:10.1016/j.biortech.2021.125563

S21. Lanfranchi E., Trajkovic M., Barta K., et al. (2019). Exploring the selective demethylation of aryl methyl ethers with a *Pseudomonas* rieske monooxygenase. *Chembiochem* **20**:118 – 125. DOI:10.1002/cbic.201800594

S22. Yoshikata T., Suzuki K., Kamimura N., et al. (2014). Three-component *O*-demethylase system essential for catabolism of a lignin-derived biphenyl compound in *Sphingobium* sp. strain SYK-6. *Appl. Environ. Microbiol.* **80**:7142 – 7153. DOI:10.1128/AEM.02236-14

S23. Cai C., Xu Z., Zhou H., et al. (2021). Valorization of lignin components into gallate by integrated biological hydroxylation, O-demethylation, and aryl side-chain oxidation. *science advance* **7**:eabg4585

S24. Mallinson S. J. B., Machovina M. M., Silveira R. L., et al. (2018). A promiscuous cytochrome P450 aromatic *O*-demethylase for lignin bioconversion. *Nat. Commun.* **9**:2487. DOI:10.1038/s41467-018-04878-2

S25. Kuatsjah E., Johnson C. W., Salvachua D., et al. (2022). Debottlenecking 4-hydroxybenzoate hydroxylation in *Pseudomonas putida* KT2440 improves muconate productivity from *p*-coumarate. *Metab. Eng.* **70**:31 – 42. DOI:10.1016/j.ymben.2021.12.010

S26. Wang H., Wang L., Chen J., et al. (2023). Promoting $FADH_2$ regeneration of hydroxylation for high-level production of hydroxytyrosol from glycerol in *Escherichia coli*. *J. Agric. Food Chem.* **71**:16681 – 16690. DOI:10.1021/acs.jafc.3c05477

S27. Xin X., Zhang R. K., Liu S. C., et al. (2024). Engineering yeast to convert lignocellulose into vanillin. *Chem. Eng. J.* **485**:149815. DOI:10.1016/j.cej.2024.149815

S28. Del Cerro C., Erickson E., Dong T., et al. (2021). Intracellular pathways for lignin catabolism in white-rot fungi. *Proc. Natl. Acad. Sci. U. S. A.* **118**:e2017381118. DOI:10.1073/pnas.2017381118

S29. Richard P., Viljanen K. and Penttila M. (2015). Overexpression of PAD1 and FDC1 results in significant cinnamic acid decarboxylase activity in *Saccharomyces cerevisiae*. *AMB Express.* **5**:12. DOI:10.1186/s13568-015-0103-x

S30. Johnson C. W., Salvachua D., Khanna P., et al. (2016). Enhancing muconic acid production from glucose and lignin-derived aromatic compounds via increased protocatechuate decarboxylase activity. *Metab. Eng. Commun.* **3**:111 – 119. DOI:10.1016/j.meteno.2016.04.002

S31. Peng X., Masai E., Kasai D., et al. (2005). A second 5-carboxyvanillate decarboxylase gene, ligW2, is important for lignin-related biphenyl catabolism in *Sphingomonas paucimobilis* SYK-6. *Appl. Environ. Microbiol.* **71**:5014 – 5021. DOI:10.1128/AEM.71.9.5014-5021.2005

S32. Lee E. G., Yoon S. H., Das A., et al. (2008). Directing vanillin production from ferulic acid by increased acetyl-CoA consumption in recombinant *Escherichia coli*. *Biotechnol. Bioeng.* **102**:200 – 208. DOI:10.1002/bit.22040

S33. Lo T. M., Chng S. H., Teo W. S., et al. (2016). A two-layer gene circuit for decoupling cell growth from metabolite production. *Cell Syst.* **3**:133 – 143. DOI:10.1016/j.cels.2016.07.012

S34. Zhang R. K., Tan Y. S., Cui Y. Z., et al. (2021). Lignin valorization for protocatechuic acid production in engineered *Saccharomyces cerevisiae*. *Green Chem.* **23**:6515 – 6526. DOI:10.1039/d1gc01442k

S35. Li J., Yue C., Wei W., et al. (2022). Construction of a *p*-coumaric and ferulic acid auto-regulatory system in *Pseudomonas putida* KT2440 for protocatechuate production from lignin-derived aromatics. *Bioresour. Technol.* **344**:126221. DOI:10.1016/j.biortech.2021.126221

S36. Manfrao-Netto J. H. C., Lund F., Muratovska N., et al. (2021). Metabolic engineering of *Pseudomonas putida* for production of vanillylamine from lignin-derived substrates. *Microb. Biotechnol.* **14**:2448 – 2462. DOI:10.1111/1751-7915.13764

S37. Fu B., Xiao G., Zhu Y., et al. (2021). Renewable vanillylamine synthesis from lignin-derived feedstocks. *ACS Agric. Sci. Technol.* **1**:566 – 571. DOI:10.1021/acsagscitech.1c00174

S38. Wu W., Liu F. and Singh S. (2018). Toward engineering *E. coli* with an autoregulatory system for lignin valorization. *Proc. Natl. Acad. Sci. U. S. A.* **115**:2970 – 2975. DOI:10.1073/pnas.1720129115

S39. Salvachua D., Rydzak T., Auwae R., et al. (2020). Metabolic engineering of *Pseudomonas putida* for increased polyhydroxyalkanoate production from lignin. *Microb. Biotechnol.* **13**:290 – 298. DOI:10.1111/1751-7915.13481

S40. Wang C., Liu R. Y., Xu T., et al. (2024). Valorizing lignin and coprecursors into homogeneous polyhydroxyalkanoates by engineered *Pseudomonas putida*. *ACS Sustainable Chem. Eng.* **12**:8402 – 8414. DOI:10.1021/acssuschemeng.4c01184

S41. Lin L., Cheng Y. B., Pu Y. Q., et al. (2016). Systems biology-guided biodesign of consolidated lignin conversion. *Green Chem.* **18**:5536 – 5547. DOI:10.1039/c6gc01131d

S42. Zhao Y., Xue L., Huang Z., et al. (2024). Lignin valorization to bioplastics with an aromatic hub metabolite-based autoregulation system. *Nat. Commun.* **15**:9288. DOI:10.1038/s41467-024-53609-3

S43. Liu H., Tao X., Ntakirutimana S., et al. (2024). Engineering *Pseudomonas putida* for lignin bioconversion into *cis-cis* muconic acid. *Chem. Eng. J.* **495**:153375. DOI:10.1016/j.cej.2024.153375

S44. Salvachúa D., Johnson C. W., Singer C. A., et al. (2018). Bioprocess development for muconic acid production from aromatic compounds and lignin. *Green Chem.* **20**:5007 – 5019. DOI:10.1039/c8gc02519c

S45. Werner A. Z., Cordell W. T., Lahive C. W., et al. (2023). Lignin conversion to β-ketoadipic acid by *Pseudomonas putida* via metabolic engineering and bioprocess development. *Sci. Adv.*

9:eadj0053. DOI: 10.1126/sciadv.adj0053

S46. Gómez-Álvarez H., Iturbe P., Rivero-Buceta V., et al. (2022). Bioconversion of lignin-derived aromatics into the building block pyridine 2, 4-dicarboxylic acid by engineering recombinant *Pseudomonas putida* strains. *Bioresour. Technol.* **346**:126638. DOI:10.1016/j.biortech.2021.126638

S47. Leonard E., Yan Y., Fowler Z. L., et al. (2008). Strain improvement of recombinant *Escherichia coli* for efficient production of plant flavonoids. *Mol. Pharmaceutics* **5**:257 – 265. DOI:10.1021/mp7001472

S48. Fowler Z. L., Gikandi W. W. and Koffas M. A. (2009). Increased malonyl coenzyme A biosynthesis by tuning the *Escherichia coli* metabolic network and its application to flavanone production. *Appl. Environ. Microbiol.* **75**:5831 – 5839. DOI:10.1128/AEM.00270-09

S49. Zhu S. Y., Liu S. C., Zhang C. X., et al. (2024). Pathway and enzyme engineering for the bioconversion of lignin derivatives into homoeriodictyol in *Saccharomyces cerevisiae*. *Green Chem.* 26:5260 – 5272. DOI:10.1039/d4gc00183d

S50. Duan L., Ding W., Liu X., et al. (2017). Biosynthesis and engineering of kaempferol in *Saccharomyces cerevisiae*. *Microb. Cell. Fact.* **16**:165. DOI:10.1186/s12934-017-0774-x

S51. Nogales J., Palsson B. O. and Thiele I. (2008). A genome-scale metabolic reconstruction of *Pseudomonas putida* KT2440: *i*JN746 as a cell factory. *BMC Syst. Biol.* **2**:79. DOI:10.1186/1752-0509-2-79

S52. Puchalka J., Oberhardt M. A., Godinho M., et al. (2008). Genome-scale reconstruction and analysis of the *Pseudomonas putida* KT2440 metabolic network facilitates applications in biotechnology. *PLoS Comput. Biol.* **4**:e1000210. DOI:10.1371/journal.pcbi.1000210

S53. Sohn S. B., Kim T. Y., Park J. M., et al. (2010). In silico genome-scale metabolic analysis of *Pseudomonas putida* KT2440 for polyhydroxyalkanoate synthesis, degradation of aromatics and anaerobic survival. *Biotechnol. J.* **5**:739 – 750. DOI:10.1002/biot.201000124

S54. Oberhardt M. A., Puchałka J., Martins dos Santos V. A., et al. (2011). Reconciliation of genome-scale metabolic reconstructions for comparative systems analysis. *PLoS Comput. Biol.* **7**:e1001116. DOI:10.1371/journal.pcbi.1001116

S55. Belda E., Van Heck R. G., José Lopez-Sanchez M., et al. (2016). The revisited genome of *Pseudomonas putida* KT2440 enlightens its value as a robust metabolic chassis. *Environ. Microbiol.* **18**:3403–3424. DOI:10.1111/1462-2920.13230

S56. Yuan Q., Huang T., Li P., et al. (2017). Pathway-consensus approach to metabolic network reconstruction for *Pseudomonas putida* KT2440 by systematic comparison of published models. *PloS one* **12**:e0169437. DOI:10.1371/journal.pone.0169437

S57. Nogales J., Mueller J., Gudmundsson S., et al. (2020). High-quality genome-scale metabolic modelling of *Pseudomonas putida* highlights its broad metabolic capabilities. *Environ. Microbiol.* **22**:255 – 269. DOI:10.1111/1462-2920.14843

S58. Roell G. W., Schenk C., Anthony W. E., et al. (2023). A high-quality genome-scale model for *Rhodococcus opacus* metabolism. *ACS Synth. Biol.* **12**:1632–1644. DOI:10.1021/acssynbio.2c00618

S59. Linz A. M., Ma Y., Scholz S., et al. (2022). iNovo479: Metabolic modeling provides a roadmap to optimize bioproduct yield from deconstructed lignin aromatics by *Novosphingobium aromaticivorans*. *Metabolites* **12**:366. DOI:10.3390/metabo12040366

S60. Peng Q., Zhao C., Wang X., et al. (2025). Modeling bacterial interactions uncovers the

importance of outliers in the coastal lignin-degrading consortium. *Nat. Commun.* **16**:639. DOI:10.1038/s41467-025-56012-8

S61. Kim J., Coradetti S. T., Kim Y. M., et al. (2020). Multi-omics driven metabolic network reconstruction and analysis of lignocellulosic carbon utilization in *Rhodosporidium toruloides*. *Front. Bioeng. Biotechnol.* **8**:612832. DOI:10.3389/fbioe.2020.612832
