# Supplementary material for: Multiscale metabolic engineering in biological lignin valorization
Source: Innovation (Camb). 2025 Jun 13;6(11):100993. doi: 10.1016/j.xinn.2025.100993 (PMC12628177; doi:10.1016/j.xinn.2025.100993)
Supplement: Document S2. Article plus supplemental information [file mmc2.pdf]

REVIEW

Multiscale metabolic engineering in biological lignin valorization

Ruo-Ying Liu,[1,2] Bing-Zhi Li,[1,2,*] Ying-Jin Yuan,[1,2] and Zhi-Hua Liu[1,2,*]
*Correspondence: bzli@tju.edu.cn (B.-Z.L.); zhliu@tju.edu.cn (Z.-H.L.)
Received: March 4, 2025; Accepted: June 11, 2025; Published Online: June 13, 2025; https://doi.org/10.1016/j.xinn.2025.100993
© 2025 The Authors. Published by Elsevier Inc. on behalf of Youth Innovation Co., Ltd. This is an open access article under the CC BY license (http://creativecommons.org/licenses/by/4.0/).

GRAPHICAL ABSTRACT

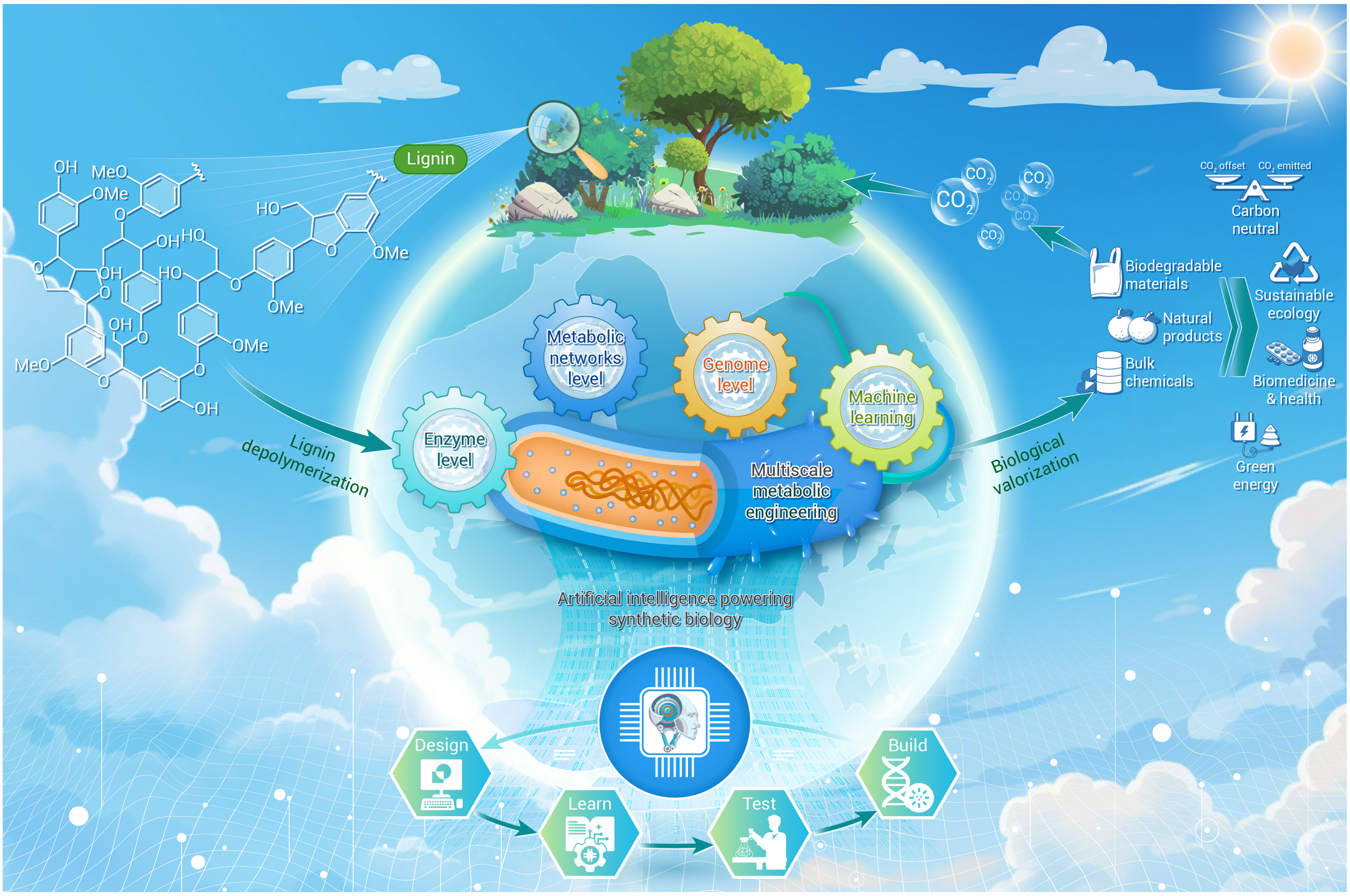

PUBLIC SUMMARY

- Multiscale metabolic engineering injects strong vigor into biological lignin valorization.
- Cutting-edge technologies in synthetic biology bring lignin bio-upgrading to an era of flexible regulation.
- Efforts are still needed to mine biological information and promote multidisciplinary integration.
- The convergence of synthetic biology with artificial intelligence will be the future trajectory.

OPEN ACCESS

REVIEW

The Innovation

Multiscale metabolic engineering in biological lignin valorization

Ruo-Ying Liu,[1,2] Bing-Zhi Li,[1,2,*] Ying-Jin Yuan,[1,2] and Zhi-Hua Liu[1,2,*]

[1]State Key Laboratory of Synthetic Biology and Frontiers Science Center for Synthetic Biology (Ministry of Education), Tianjin University, Tianjin, China

[2]School of Synthetic Biology and Biomanufacturing, Tianjin University, Tianjin, China

*Correspondence: bzli@tju.edu.cn (B.-Z.L.); zhliu@tju.edu.cn (Z.-H.L.)

Received: March 4, 2025; Accepted: June 11, 2025; Published Online: June 13, 2025; https://doi.org/10.1016/j.xinn.2025.100993

© 2025 The Authors. Published by Elsevier Inc. on behalf of Youth Innovation Co., Ltd. This is an open access article under the CC BY license (http://creativecommons.org/licenses/by/4.0/).

Citation: Liu R.-Y., Li B.-Z., Yuan Y.-J., et al., (2025). Multiscale metabolic engineering in biological lignin valorization. The Innovation **6(11)**, 100993.

Lignin, a natural and renewable aromatic polymer, serves as a plentiful source of aromatic building blocks for biomanufacturing. However, despite its potential, the bioconversion efficiency of lignin remains limited due to its inherently complex structure and the constraints of traditional metabolic engineering approaches. Synthetic biology-guided metabolic regulation offers a solution by coordinating intracellular resource allocation in ligninolytic strains, endowing their adaptation for industrial applications and promoting the sustainability of lignin-based bioeconomy. This review provides a comprehensive overview of multiscale metabolic regulation, including critical enzymes involved in biotransformation, metabolic pathway networks, genome-phenotype, and learning prediction. It highlights the significant roles and potential benefits of emerging cutting-edge technologies in advancing lignin valorization. Overall, synthetic biology-guided metabolic regulation has demonstrated its power in balancing the metabolic fluxes of ligninolytic strains, ensuring the continued vitality in future development.

INTRODUCTION

Lignin is the most abundant aromatic macromolecule biopolymer in nature, formed by oxidative coupling reactions of three main aromatic alcohol monomers—coniferyl, sinapyl, and *p*-coumaryl alcohol.[1] Additionally, other phenolic compounds (e.g., flavonoids, hydroxystilbenes, and hydroxycinnamic amides) possibly act as monomers and become building blocks of lignin polymer.[2] It exhibits significant bioactivity due to the diverse functional groups on these monomers, such as phenolic hydroxyl and methoxy groups.[3] It also possesses unique resource advantages, including renewability, biodegradability, and biocompatibility.[4] In recent years, as the "waste-to-treasure" concept has gained traction, the upgrading of lignin has attracted widespread attention.[5] Harnessing lignin resource is a necessary way toward reducing reliance on fossil fuels and addressing the challenges of economic, environmental, and industrial sustainability.[6,7] For example, according to the life cycle assessment, the carbon emission of lignin-based adipic acid is 4.87 kg CO_2/kg of acid, which is a reduction of 62%–78% compared with the petrochemical production route.[8] It is worth mentioning that with the vigorous development of synthetic biology, the development of microbial systems for lignin valorization offers a new green and sustainable strategy.

The first link in achieving lignin biological valorization is to obtain oligomers or monomers that can cross microbial cellular membranes.[9] Advanced lignocellulose fractionation and lignin depolymerization technologies have been applied to obtain bioavailable lignin derivatives.[10] For example, efficient pretreatment strategies—such as alkaline, extractive-ammonia, and organosolv methods—combined with reductive, oxidative, or biological depolymerization yield lignin-derived oligomers and monomers (Table S1).[4,11,12] Under reductive depolymerization, lignin fractions are converted into aromatic monomers (e.g., methylparaben, β-hydroxypropiovanillone, and acetovanillone) using redox catalysts and reducing agents.[13] Oxidative depolymerization, in contrast, cleaves lignin side chains via oxidants to generate phenolic compounds such as phenol and guaiacol.[14,15] Nature also offers microbial solutions, while ligninolytic microbes employ peroxidases (e.g., lignin peroxidases, manganese peroxidases, versatile peroxidases, and dye-decolorizing peroxidases) to non-enzymatically fragment lignin via radical-mediated reactions, producing heterogeneous aromatic monomers such as *p*-coumaric acid, ferulic acid, and vanillic acid.[16–18]

Billions of years of evolution have also enabled ligninolytic microbes in nature to develop versatile enzymatic toolboxes and diverse metabolic pathways to catabolize depolymerized lignin-derived compounds.[19] They can converge heterogeneous lignin-derived aromatics into central compounds, including protocatechuic acid, catechol, and gallic acid via the upper metabolic pathway, which acts as a "biological funnel."[20] These central compounds undergo subsequent ring-opening pathways and ultimately enter the tricarboxylic acid cycle as a source of energy supply.[9] However, the inherent heterogeneity, aromaticity and multifunctionality of lignin lead to its biotransformation inevitably undergoing many energy-consuming metabolic processes. The coordinated transformation of lignin functional groups, such as hydroxylation, *O*-demethylation, decarboxylation, and subsequent aromatic ring cleavage, imposes strict requirements on the homeostasis of bacteria.[9,21] Engineered pathways of lignin biotransformation depend on chassis strains to provide transcriptional and translational enzymes, adenosine triphosphate (ATP), various cofactors, and other endogenous metabolites. However, physiological imbalances in these engineered microorganisms—such as metabolic burden, cofactor supply chain bottlenecks, and the accumulation of toxic aromatic metabolites—hinder efficient lignin bioconversion.[22] For industrial-scale production, optimal engineered strains should achieve high lignin conversion efficiency, high product titers, and robust performance stability. To address these challenges, intelligent fine-grained metabolic regulation—enabled by interdisciplinary approaches for precise temporal and/or spatial control—offers a promising strategy for efficient lignin bio-upgrading.[23]

Metabolic engineering has endowed microorganisms with the capacity to produce a variety of target chemicals.[24] However, designing artificial biological funnel routes of lignin mechanistically by rewriting complex inherent pathways would disrupt the resource allocation within the microbial strains, limiting the yield and market competitiveness of bio-based products.[25] Fortunately, creative integration of multidisciplinary and new technologies has propelled the field of systems metabolic engineering into an unprecedented era of sophistication.[26] By complementing metabolic engineering with molecular biology, genome-scale metabolic modeling, and machine learning, we can now achieve more precise and programmable regulation of cellular phenotypes.[27] Molecular biology provides powerful toolboxes for stably reprogramming the genetic properties of host bacteria at the genomic level.[28] Genome-scale metabolic modeling guides metabolic engineering by optimizing reaction rate ratios, leveraging the cascade relationships among genes, enzymes, and metabolic reactions.[29] Moreover, machine learning relies on big data and language modeling to rapidly predict the optimal synthetic pathway for a specific bioproduct, greatly diminishing the blindness of metabolic regulation.[30,31] Consequently, this integration of cutting-edge disciplines is poised to propel lignin valorization toward unprecedented advancements.

This work thus aims to summarize the state-of-the-art strategies of multiscale metabolic regulation for lignin valorization. Herein, precise regulation of key enzymes is anticipated to facilitate lignin aromatic ring activation. Moreover, metabolic network-level interactions are summarized for promoting balanced intracellular resource allocation and enabling the construction of orthogonal, controllable metabolic pathways. Genome-scale metabolic modeling provides mathematical frameworks to simulate metabolic networks in chassis strains, while computational tools leverage microbial multi-omics data to generate predictive biological insights. Together, these integrated strategies could offer a systematic framework for designing efficient cell factories for lignin valorization, advancing the development of a sustainable bio-based economy.

REGULATION OF KEY ENZYMES TO DE-DIVERSIFY THE FUNCTIONAL GROUPS OF LIGNIN

Microorganisms in nature have evolved a "biological funnel" to overcome lignin heterogeneity.[32] Lignin macromolecules first undergo depolymerization to produce a mixture of aromatic monomers, which are transported into the cell and metabolized into specific shared aromatic intermediates, such as

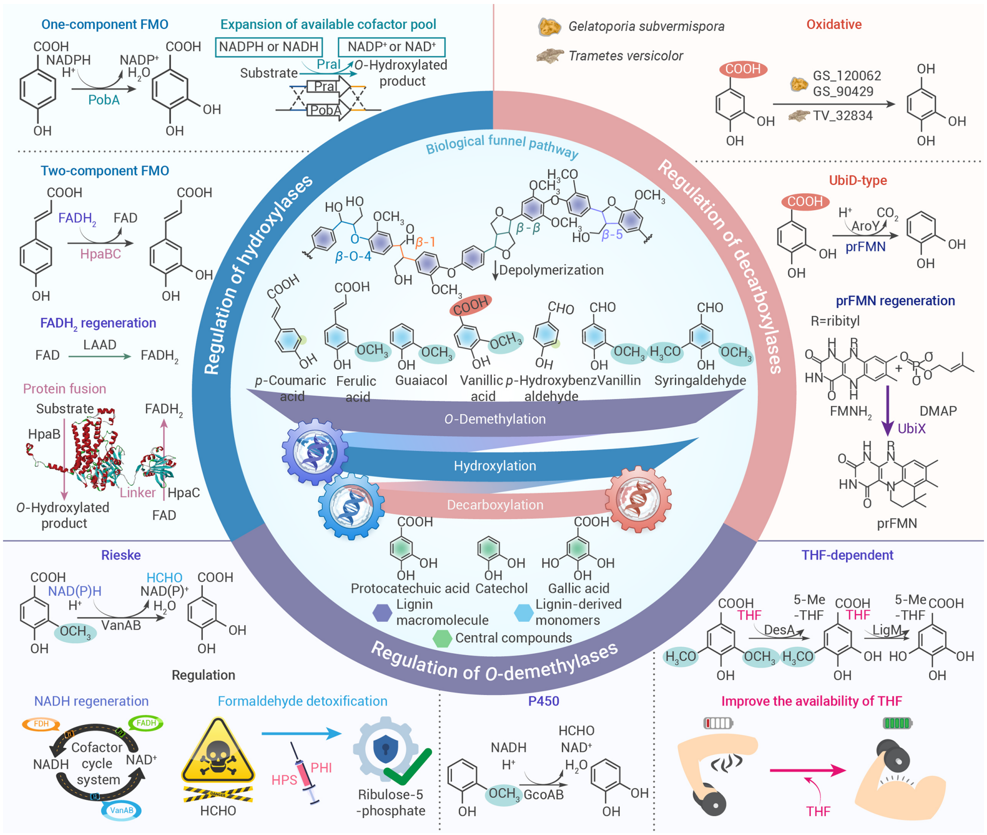

Figure 1. Critical enzymatic catalytic steps and regulatory strategies in microbial catabolism of lignin Heterogeneous aromatic compounds derived from the depolymerization of lignin are converted to central platform compounds via a "biological funnel" route involving *O*-demethylation, hydroxylation and decarboxylation. AroY, protocatechuate decarboxylase; DesA, syringate *O*-demethylase; FADH, glutathione-independent formaldehyde dehydrogenase; FDH, formate dehydrogenase; GcoAB, aromatic *O*-demethylase; HpaB, 4-hydroxyphenylacetate 3-monooxygenase oxygenase component; HpaC, 4-hydroxyphenylacetate 3-monooxygenase reductase component; LAAD, L-amino acid deaminase; LigM, vanillate/3-*O*-methylgallate *O*-demethylase; PobA, *p*-hydroxybenzoate hydroxylase; PraI, 4-hydroxybenzoate 3-monooxygenase; UbiX, flavin prenyltransferase; VanAB, vanillate *O*-demethylase oxygenase.

protocatechuic acid, gallic acid, and catechol.[33] In this process, enzymes such as *O*-demethylases, hydroxylases, and decarboxylases, which modify aromatic rings, play a crucial role (Figure 1).[21] As engineered microorganisms are increasingly employed in the production of lignin-based chemicals, these three enzyme-mediated reactions are often considered rate-limiting steps.[34,35] Understanding the catalytic mechanism, enzymatic properties, and rational regulation of these enzymes is critical for optimizing lignin valorization in biotechnological applications. The implementation of "addition and subtraction" in the bioconversion process, based on the specific characteristics of the catalytic reactions, is a potential strategy to optimize the catalytic performance of key enzymes. For "addition," strategies such as increasing the cofactor pool and maintaining a cellular equilibrium state can empower enzymes. And for "subtraction," strategies such as reducing the accumulation of by-products and eliminating feedback inhibition can reduce the burden on key enzymes.

The prevalence of coniferyl and sinapyl alcohol units in lignin results in its depolymerization producing a large number of methoxylated derivatives.[36] Microorganisms can perform *O*-demethylation through three classes of enzymes, namely Rieske non-heme iron oxygenases (ROs), cytochrome P450s (CYP450s), and tetrahydrofolate (THF)-dependent demethylases (Table S2).[21] Among them, ROs and CYP450s catalyze the activation of molecular oxygen and NAD(P)H-mediated electron transfer in a multi-component form, with hemiacetal compounds as intermediates, ultimately producing demethylated products and formaldehyde.[37,38] In contrast, THF-dependent demethylases catalyze the electron transfer between lignin derivatives and THF in a nonoxidative mode.[39] For ROs and CYP450s, formaldehyde produced during *O*-demethylation causes severe damage to enzymes and cells.[40] Therefore, relieving its toxic effects is an effective strategy to improve the catalytic activity of enzymes and the bioconversion efficiency of lignin (Figure 1). Currently, the ribulose monophosphate pathway has been extensively studied for the assimilation of formaldehyde.[41,42] The enhanced ribulose monophosphate pathway can relieve the biological toxicity of formaldehyde to efficiently regulate the catalytic activity of *O*-demethylases. Lignin derivatives containing methoxy groups, such as ferulic acid and vanillic acid, can be more readily metabolized or converted into high-value compounds.[41,42] By increasing the metabolic flux of the ribulose monophosphate pathway in *Burkholderia cepacia* TM1, which enables the utilization of methylated lignin monomers as a carbon source, the engineered strain exhibited significantly enhanced vanillic acid degradation and growth yield compared with the wild-type strain, even in the presence of up to 60 mM vanillic acid.[41] Furthermore, engineered *Pseudomonas putida* KT2440 with an enhanced ribulose monophosphate pathway produced protocatechuic acid at a titer of 6.73 mg/mL when using ethanol-assisted depolymerized lignin as a substrate, representing a 49.2% increase in yield compared with the control strain. The engineered strain also exhibited more robust cell growth, with a 27.6% increase in optical density.[42] It is worth mentioning that various biosensors responding to formaldehyde have been studied and they are promising for dynamically regulating the expression levels of *O*-demethylases and the formaldehyde assimilation pathway enzymes, maintaining formaldehyde levels within a bio-nontoxic range.[40,43]

Additionally, the maintenance of redox homeostasis is essential for regulating the catalytic process of *O*-demethylases (Figure 1). Imbalances in cofactor pairs such as $NAD(P)^+$/NAD(P)H, FMN/$FMNH_2$ and FAD/$FADH_2$ due to the purposeful modification can affect the efficiency of key enzymes.[44] Therefore, the industrial production of various high-value compounds coordinates the ratio between the reduced and oxidized forms of cofactors via multi-strategy.[45] For the biological valorization of lignin, the construction of a NADH regeneration system to maintain the efficient functioning of the electron transport chain cannot be ignored for the regulation of *O*-demethylase activity. A biocatalyst was obtained by constructing recombinant *Escherichia coli* that heterologously expressed an oxygenase-type vanillic acid *O*-demethylase (VanAB) and cofactor regeneration systems.[46] The production system included freeze-dried cells, NADH, $FeSO_4$, dithiothreitol, and vanillic acid. The dual-function system was designed with formaldehyde elimination and NADH regeneration mediated by formaldehyde dehydrogenase, resulting in the yield of protocatechuic acid at 56.5%. When additionally coupled with formate dehydrogenase, the yield of protocatechuic acid increased to 84.4%, and the biocatalyst achieved its highest titer of 4.1 mM.[46]

For THF-dependent demethylases, THF was used as a methyl acceptor to produce 5-methyl-THF instead of formaldehyde. A classic example is the LigM-catalyzed *O*-demethylation of vanillic acid. H_4folate-N_5 acts as a nucleophile in the bimolecular substitution, and its lone pair of electrons attacks the methyl carbon of vanillic acid.[47] Therefore, the availability of THF is an important constraint on *O*-demethylases. The construction of a THF regeneration system is an effective strategy to facilitate the efficient conversion of S- or G-type lignin substrates toward the target product. Co-expression of LigM and MetE in *E. coli* significantly reduced the THF cofactor required for vanillic acid *O*-demethylation, with the THF/substrate molar ratio reduced by 500-fold.[48] When only DesA and LigM from *Sphingobium* sp. SYK-6 were heterologously expressed in *Rhodococcus opacus*, *O*-demethylation of syringic acid was not observed. However, when an additional 0.5 mM of H_4folate was added to the culture system, the titer of gallic acid reached 0.146 mM.[49] In *Saccharomyces cerevisiae*, the engineered strain heterologously expressing only LigM obtained a

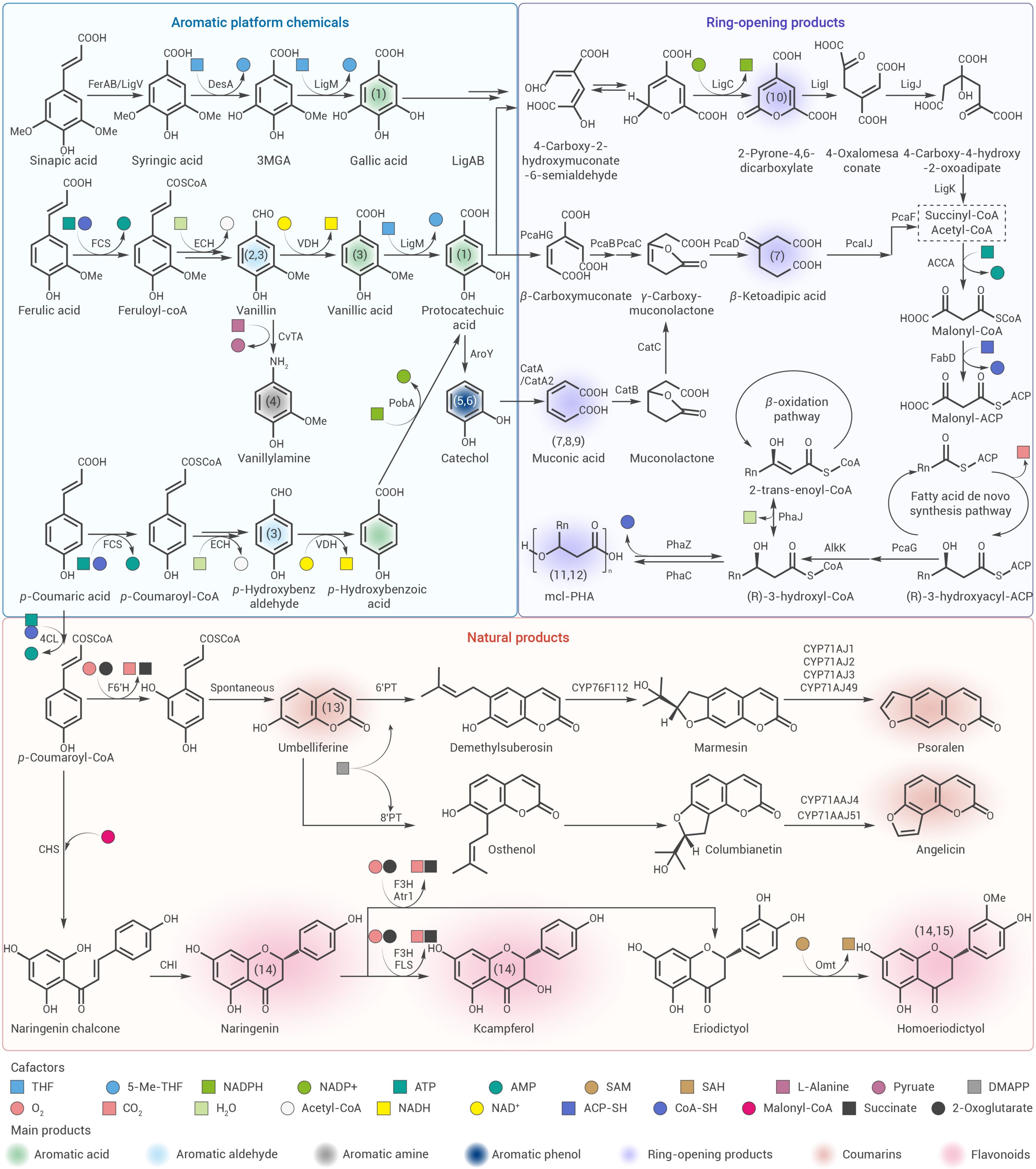

Figure 2. Metabolic networks for valorizing heterogeneous lignin derivatives to high-value chemicals, including aromatic platform chemicals, ring-opening products and aromatic natural products The numbers labeled on the chemicals correspond to the regulatory strategies in Figure 3. 4CL, 4-coumarate CoA ligase; ACCA, acetyl-coenzyme A carboxylase carboxyl transferase; AlkK, medium-chain-fatty-acid-CoA ligase; AroY, protocatechuate decarboxylase; BenABC, benzoate 1,2-dioxygenase; BenD, 1,6-dihydroxycyclohexa-2,4-diene-1-carboxylate dehydrogenase; CatA/CatA2, catechol 1,2-dioxygenase; CatB, *cis,cis*-muconate cycloisomerase; CatC, muconate cycloisomerase; CHI, chalcone isomerase; CHS, chalcone synthase; DesA, syringate *O*-demethylase; ECH, enoyl-CoA hydratase; F6′H, feruloyl-CoA 6′-hydroxylase; F3H, flavanone 3-hydroxylase; FabD, malonyl CoA-ACP transacylase; FCS, feruloyl-CoA synthase; FerA, feruloyl-CoA synthetase; FerB, feruloyl-CoA hydratase/lyase; FLS, flavonol synthase; LigAB, protocatechuic acid 4,5-dioxygenase; LigC, 4-carboxy-2-hydroxymuconate-6-semialdehyde dehydrogenase; LigH, 10-formyltetrahydrofolate synthetase; LigI, 2-pyrone-4,6-dicarboxylate hydrolase; LigJ, 4-oxalomesaconate hydratase; LigK, 4-carboxy-4-hydroxy-2-oxoadipate aldolase; LigM, vanillate/3-*O*-methylgallate *O*-demethylase; LigV, vanillin dehydrogenase; PcaB,

(legend continued on next page)

protocatechuic acid titer of 12 mg/L. L-Methionine synthesis and THF regeneration were regulated by overexpressing endogenous MET6 and knocking out MHT1 and SAM4.[50] While MetF1 from *R. dicambivorans* Ndbn-20 was heterologously expressed to maximize the availability of the cofactor. Regulating the rate-limiting step of *O*-demethylation achieved 33 mg/L of protocatechuic acid from ferulic acid.[50]

The enzymes that catalyze the hydroxylation of lignin derivatives are mainly flavin monooxygenases (Figure 1; Table S2).[51] Flavin monooxygenases activate molecular oxygen in single or multi-component form, inserting one oxygen atom into the lignin substrate to catalyze its hydroxylation, and another oxygen atom is reduced to a water molecule. In this process, NAD(P)H is used as a hydrogen donor.[52] Increasing the availability of cofactors can promote the hydroxylation of lignin substrates. In *P. putida* KT2440, the endogenous hydroxylase PobA, which strictly accepts NADPH, was replaced with PraI, a hydroxylase capable of accepting both NADH and NADPH. The modification eliminates the rate-limiting step of the *p*-hydroxybenzoic acid hydroxylation and decreases the accumulation of the intermediate 4-hydroxybenzoic acid by 50% at 12 h. The engineered strain produced a final titer of muconic acid at 40 g/L, with a yield of 100%.[53] The $FADH_2$ regeneration system mediated by L-amino acid deaminase and the NADH regeneration system mediated by formic acid dehydrogenase also promoted the aromatic hydroxylation catalyzed by HpaBC.[54,55] For the two-component flavin monooxygenase, coordinating the interactions between the cooperating components is also a promising option for modulating catalytic efficiency. In *S. cerevisiae*, $(GGGGS)_3$ linker was used to construct a fusion protein of HpaB and HpaC, and the efficiency of hydroxylation of hydroxybenzaldehyde to protocatechuic aldehyde was greatly improved. The engineered strain employing the enzyme fusion strategy achieved a protocatechualdehyde titer of 0.78 mM, representing an increase of over 100% compared with the control strain.[56]

Decarboxylation reactions are divided into nonoxidative and oxidative types, depending on whether the carboxyl groups in lignin derivatives are catalyzed to form hydrides or hydroxyls (Figure 1).[21] The decarboxylases identified in ligninolytic bacteria mainly belong to the amidohydrolase superfamily or the UbiD-type family, catalyzing the nonoxidative decarboxylation of lignin derivatives.[57,58] Oxidative decarboxylases have been found in white-rot fungi, catalyzing the conversion of 4-hydroxybenzoic acid and protocatechuic acid into hydroquinone and 1,2,4-benzenetriol, respectively (Table S2).[59] For UbiD-type decarboxylase, a typical research target is ferulic acid decarboxylase and phenylacrylic acid decarboxylase from *S. cerevisiae*. Phenylacrylic acid decarboxylase can synthesize the essential cofactor prenylated flavin (prFMN) of ferulic acid decarboxylase using dimethylallyl pyrophosphate as the isoprene donor.[60] Another classic case is protocatechuic acid decarboxylase. Such enzymes are often co-expressed in microorganisms with other small genes in an operon. These small genes can significantly regulate the catalytic activity of protocatechuic acid decarboxylase.[61] Although the function of the small-gene products, known as BCD proteins, remains to be fully elucidated, the auxiliary decarboxylation mechanism of phenylacrylic acid decarboxylase—a homolog of B protein—has been characterized. B protein may also be involved in cofactor synthesis.[62] Based on this catalytic property, auxiliary protein co-expression strategies have been widely applied to enhance the decarboxylation activity of protocatechuic acid decarboxylase for the efficient production of muconic acid from lignin derivatives.[62–64] Specifically, the co-expression of related proteins led to more than a 3-fold improvement in the titer, yield, and productivity of muconic acid, with the titer reaching 4.92 g/L.[62]

O-Demethylation, hydroxylation and decarboxylation are the major rate-limiting steps in lignin valorization. Modulation of the enzymes performing these three types of reactions is essential to overcome the bottleneck of lignin conversion. These enzymes belong to different classes with significant differences in catalytic mechanism, required cofactors and by-product generation. Therefore, targeting the specific catalytic features of key enzymes—through strategies such as by-product removal, cofactor regeneration, and auxiliary protein co-expression—can significantly enhance catalytic activity. Notably, with the ongoing advancement of biotechnology, the understanding of lignin metabolic enzymes continues to improve, providing insights for developing multi-faceted regulatory strategies for key enzymes.

RATIONALLY RESHAPING METABOLIC NETWORKS TO PROMOTE LIGNIN BIOCONVERSION

After a long period of evolution, ligninolytic microbes have formed a variety of sophisticated and cooperative metabolic pathways, which are crosslinked and influence each other to form a complex aromatic metabolic network (Figure 2).[65] The metabolic network can respond to specific biological and environmental triggers by regulating the biosynthesis of metabolites, cofactors and energy.[66,67] Although the rich biochemical information offers a valuable alternative reaction library for the lignin biological valorization, even slight disturbances within it can affect various functional modules.[68] In particular, the structural characteristics of lignin lead to its depolymerization to heterogeneous aromatic derivatives. The complex microbial metabolic network of these heterogeneous derivatives undoubtedly poses a challenge to the atom economic conversion of lignin. Additionally, the integration of metabolic modules for multiple types of lignin monomers places a heavy burden on chassis strains. The defects caused by the modifications of the primary purpose can be cured by engineering the basic metabolic modules of the strain, such as the tricarboxylic acid cycle and fatty acid synthesis. Therefore, rationally reshaping the metabolic networks of engineered microorganisms can guide biological processes in a way that better supports biomanufacturing.

Static regulation of microbial metabolic networks promotes collaboration among each lignin metabolism functional module and is widely used for the upgrading of lignin to high-value chemicals (Figure 3; Table S3). Regulating the upstream pathways of the “biological funnel” and optimizing background metabolic networks to ensure a more harmonious function of the modules is an effective strategy for enhancing the performance of engineered strains in producing aromatic platform compounds. To produce vanillin, the *fcs* and *ech* genes were heterologously expressed in *E. coli*. The excess acetyl-CoA produced in this pathway hinders the forward progress of production. Therefore, overexpressing *gltA* and knocking out the *icdA* gene, both of which are involved in the tricarboxylic acid cycle, converted acetyl-CoA into CoA required for FCS-catalyzed acetylation. The final vanillin titer was as high as 5.14 g/L, and the yield was 86.6%.[69] In *S. cerevisiae*, a biosynthetic module for vanillin was constructed and optimized by introducing heterologous synthesis pathways and blocking the branched metabolic pathways.[56] This module was then combined with an *S*-adenosylmethionine (SAM) regeneration module. The developed biosystem successfully produced 2.49 and 1.94 mmol/L vanillin from lignin-derived ferulic acid and *p*-coumaric acid, respectively. Notably, by constructing a xylose utilization module and regulating the carbon flux of the shikimate pathway, a yield of 10.5 mg vanillin/g carbon source was ultimately obtained from real lignocellulosic biomass hydrolysates.[56] Furthermore, the carbon flux of lignin-derived *p*-coumaric acid and ferulic acid was directed to the biosynthesis of protocatechuic acid to the greatest extent, and in combination with a THF regeneration system, 810 mg/L of protocatechuic acid was produced from alkaline pretreatment liquor.[50] Besides, a production system containing three gallic acid synthesis pathways and a THF regeneration system was integrated into *R. opacus*, and gallic acid was successfully produced from heterogeneous lignin derivatives in a one-pot process.[49] For the production of vanillylamine, the coordination of the heterologous synthesis of the product and the removal of by-products is essential.[70] Pyruvate is produced in the reversible amination reaction of vanillin. The implementation of alanine dehydrogenase to recover this byproduct made the reaction proceed in a direction that was more conducive to amination.[70]

It is worth mentioning that many ligninolytic microbes have evolved aromatic ring-opening pathways.[71] Modulation of the biological funnel downstream routes and the background metabolic network within the engineered strain allows for efficient microbial production of the ring-opening products (Figure 3; Table S3). Polyhydroxyalkanoates (PHAs) are promising precursors for the manufacture of bio-degradable bioplastics.[72] The carbon flux from *p*-coumaric acid to PHA was maximized through the regulation of several modules, including

3-carboxy-*cis*,*cis*-muconate cycloisomerase; PcaC, 4-carboxymuconolactone decarboxylase; PcaD, 3-oxoadipate enol-lactonase; PcaF, 4-hydroxy-2-oxovalerate aldolase; PcaG, protocatechuate 3,4-dioxygenase; PcaHG, protocatechuate 3,4-dioxygenase; PcaJ, 3-oxoadipate CoA-transferase; PHA, polyhydroxyalkanoates; PhaC, poly(3-hydroxyalkanoate) polymerase; PhaZ, poly(3-hydroxyalkanoate) depolymerase; PobA, *p*-hydroxybenzoate hydroxylase; PT, prenyltransferase; VanAB, vanillate *O*-demethylase oxygenase; VDH, vanillin dehydrogenase.

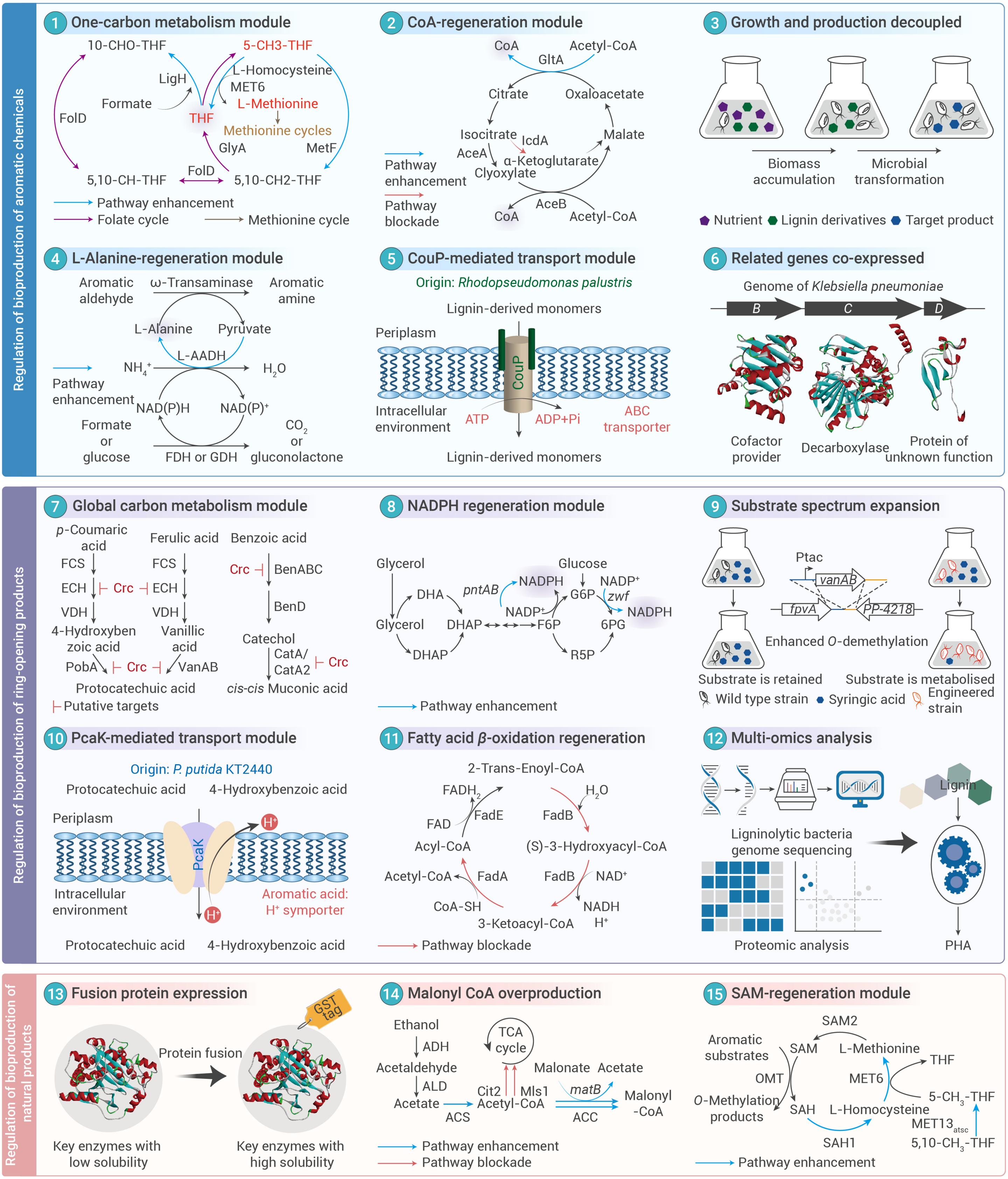

Figure 3. The regulation and coordination of multiple modules in the metabolic network enable efficient bioconversion of lignin into high-value products AceA, isocitrate lyase; AceB, malate synthase; ACC, acetyl-CoA carboxylase; ACS, acetyl-CoA synthetase; ADH, alcohol dehydrogenase; ALD, aldehyde dehydrogenase; Cit2, citrate synthase; CrC, catabolite repression control protein; CvTA, 8-amino-7-oxononanoate synthase; FadA, 3-ketoacyl-CoA thiolase; FadB, enoyl-CoA hydratase; FadE, acyl-CoA dehydrogenase; FDH, formate dehydrogenase; FolD, methenyltetrahydrofolate cyclohydrolase; GDH, glucose 1-dehydrogenase; GltA, citrate synthase; GlyA, glycine hydroxymethyltransferase; IcdA, isocitrate dehydrogenase; L-AAD, L-amino acid deaminase; MatB, malonate synthetase; MET6, homocysteine S-methyltransferase; MetF, 5,10-methylenetetrahydrofolate reductase; Mls1, malate synthase 1; OMT, caffeoyl-CoA *O*-methyltransferase; PntAB, proton-pumping counterpart; SAH1, SAH hydrolase; Zwf, glucose-6-phosphate dehydrogenase.

the PHA accumulation module, fatty acid synthesis module, and β-oxidation module.[73] The combinatorial regulation strategy of the metabolic network increased PHA production by an astonishing 200%.[73] Excitingly, multi-omics analyses provide a solid foundation for a comprehensive mapping of lignin microbial metabolism. Through genomics and proteomics, the mechanism of lignin metabolism by *P. putida* A514 was clearly elucidated.[74] In *P. putida* A514, dye-decolorizing peroxidase DyP is responsible for breaking the C_α-C_β bond to achieve lignin depolymerization in the presence of hydrogen peroxide, while oxidases involved in peripheral reactions are responsible for generating hydrogen peroxide. The lignin depolymerization module was further successfully constructed by screening efficient promoters, *dyP* genes and signal peptides. Additionally, the aromatic hydrocarbon metabolism module was constructed by overexpressing VanAB. This module enhanced the β-ketoadipic acid pathway and improved lignin metabolism by the engineered strain. Moreover, the PHA synthesis module was successfully constructed by overexpressing *phaJ4* and *phaC1* genes to regulate the metabolic flux from the fatty acid β-oxidation pathway to the PHA biosynthesis pathway. Coupling the three functional modules led to a 6-fold increase in PHA titer produced by the engineered strain from biorefinery waste, reaching 160 mg/L.[74] Another promising candidate for the development of lignin-based polymer is *cis-cis* muconic acid.[71] According to the techno-economic analysis, the price of muconic acid produced from first-generation sugars reaches $1.95/kg, while starting from lignin monomers it would be reduced to $0.86/kg.[71] The engineered *P. putida* KT2440 incorporates three complementary functional modules: a heterologous muconic acid synthesis module, an S-type lignin utilization module, and an NADPH regeneration module. After multi-module regulation, the engineered strain successfully produced muconic acid from three types of lignin monomers with a titer of 13.1 mM and a yield of up to 99.5%.[75] Meanwhile, this strategy achieved the simultaneous conversion of all three types of lignin monomers, offering a promising approach to address lignin heterogeneity. It is worth mentioning that synthetic biology-guided metabolic modeling allowed for obtaining a mathematical model of core carbon metabolism and muconic acid biosynthesis in *P. putida* KT2440. The potential regulatory targets were obtained by using constrained minimum cut sets and elementary flux vectors. The regulation of metabolic modeling successfully increased the yield of muconic acid from 6.4% to 33.3%.[71] β-Ketoadipic acid is a precursor of nylon-6,6 analog, and its efficient bioproduction is conducive to improving the market competitiveness of bio-based polymer materials.[76] Construction of the product production module in *P. putida* KT2440 by enhancing the upstream lignin-derived aromatics pooling to protocatechuic acid and blocking the downstream β-ketoadipic acid degradation pathway.[77] Subsequently, the intracellular carbon metabolism was regulated by knocking out the global regulator Crc. The combined regulation allowed the microbial production of β-ketoadipic acid to be sold at a gratifyingly low price of US$2.01/kg.[77] Similarly, weakening the carbon catabolite repression by knocking out Crc can also promote the production of muconic acid from *p*-coumaric acid, with the yield increased by about 70%.[78] For biological lignin valorization, another critical functional module that requires regulation is the aromatic substrate transport module. The availability of hydroxylated aromatics and the production of target products are constrained by the efficiency of substrate transport into the cell.[79] By blocking the endogenous β-ketoadipate pathway in KT2440 and heterologously expressing the synthesis pathway of 2-pyrone-4,6-dicarboxylic acid, a product synthesis module was constructed. Its cooperation with the pcaK-mediated enhanced protocatechuic acid transport module resulted in an 8-fold increase in productivity to 0.58 g/L/h.[79]

For aromatic natural products, their synthesis in microorganisms usually requires the recombination of long heterologous metabolic pathways, which severely limits their biological production performance.[80] Therefore, regulating the metabolic network to strike a delicate balance between product formation and cell metabolism offers a new bioconversion strategy for lignin valorization into aromatic natural products (Figure 3; Table S3). The homoeriodictyol synthesis module, CoA supply module and SAM regeneration module were assembled in *S. cerevisiae*, and a product titer of 3.2 mM was successfully obtained from lignin-derived *p*-coumaric acid.[81] Meanwhile, the proximity effect and heterologous expression efficiency of enzymes in the long pathway were regulated by fusion protein strategies, promoting the bio-upgrading of lignin to simple coumarins umbelliferone.[82,83] However, there is still a gap in the synthesis of furanocoumarins from lignin. Interestingly, the carbon flux of glycerol was concentrated toward *p*-coumaric acid by regulating the aromatic amino acid biological metabolic network in *S. cerevisiae*. Subsequently, it cascaded with the umbelliferone synthesis module and the marmesin synthesis module. Among them, the marmesin synthesis module was constructed and optimized through heterologous expression of the synthetic pathway and truncation of the transmembrane domain of the rate-limiting enzymes.[84] Given the easy availability of *p*-coumaric acid from lignin, this undoubtedly provides strategic guidance for efficiently and concisely regulating the metabolic network for the biotransformation of lignin to furanocoumarins.[85,86]

Dynamic regulation can promptly adjust metabolite levels to maintain an appropriate balance in response to changes in metabolic signals.[87] Microorganisms in nature have evolved a variety of promoters that respond to different signal molecules, providing an element box for dynamic regulation.[88] The characterization and implementation of these sensitive promoters have enabled innovative uses of biosensors and gene circuits in lignin biological valorization.[89] In order to alleviate the resource allocation pressure caused by the simultaneous cell growth and product synthesis, it is necessary to decouple these two processes.[90] The key to achieving dynamic regulation depends on the selection of transcription factors that are sensitive to lignin-derived aromatic monomers. The degradation regulator PP3359 repressed the promoter of the *ech* gene, and this repression was mitigated by hydroxycinnamic CoA. The *fcs* gene was placed under the control of the promoter of the *csiD* gene, whose activity increased with decreasing glucose. While *ech* and *vdh* genes were placed under the control of the promoter of *ech* gene, and PP3359 was set up for constitutive expression. The engineered strain containing this two-layered circuit could preferentially accumulate biomass. Subsequently, the expression of *fcs* gene was initiated to convert lignin-derived aromatic monomers into the corresponding thioester intermediates in order to relieve the inhibition of the expression of *ech* and *vdh* genes by PP3359. The genetic circuit thus developed resulted in a 2-fold increase in the growth rate, accompanied by the production of vanillic acid at titers up to 880 mg/L.[90] Furthermore, limiting the expression of toxic pathway enzymes to the target compound synthesis phase can effectively balance cell growth and bioproduction.[91] Through transcriptomic analysis, the promoter P_{urtA} that responds to nitrogen starvation signals was screened out and combined with T7 RNA polymerase and T7 lysozyme to develop a nitrogen-limitation biosensor. Dynamic regulation of the cytotoxic pathway enzyme CadA using this biosensor significantly improved cell growth and provided an informative strategy for promoting the conversion of lignin to itaconic acid.[91] Additionally, the development of the biosensor responsive to lignin-derived *p*-coumaric acid can intelligently sense lignin substrates and dynamically regulate the lignin metabolism network.[92] The biosensor uses *p*-coumaric acid to bind to the PadR protein, causing it to dissociate from the promoter P_{padC}, thereby initiating the protocatechuic acid synthesis metabolic network. This dynamic modulation strategy resulted in a titer of 12.7 g/L of protocatechuic acid.[92] Lignin-derived vanillin has also been used as an inducer to develop an autoregulatory system for the valorization of lignin to catechol.[93] Vanillin-inducible promoter ADH7 was applied to dynamically control the catechol biosynthetic module and aromatic transport module. This strategy delivered an answer to overcome the challenges of biotoxicity of lignin-derived aromatics and low economics caused by expensive exogenous inducers.[93] Additionally, many ligninolytic microbes have evolved a variety of gene regulatory elements that can respond to lignin-derived aromatic monomers. The application of substrate metabolic analysis, functional gene annotations and RT-qPCR successfully identified a variety of aromatic metabolic regulatory elements in *Ralstonia eutropha* H16.[94] The regulatory protein PcaQ and its activated promoter P_{pca} were utilized to construct an intelligent self-induction system, which could respond to different types of lignin-derived monomers and thus regulate the expression of rate-limiting enzymes. Moreover, rational protein engineering of PcaQ through hot spots prediction and molecular docking resulted in a mutant $PcaQ^{R145K}$ with high sensitivity. The engineered strain that assembled multifunctional modules successfully accumulated polyhydroxybutyrate from the alkaline pretreatment liquid of *Pinus massoniana* with a record titer of 2.38 g/L.[94] Notably, natural or engineered regulatory elements provide an instrumental basis for the hierarchical metabolic transformation of heterogeneous lignin derivatives.[95] The development of an aromatic-sensitive intelligent regulatory system is expected to enable the time-sequential regulation

of multi-substrate transformations. Automated dynamic regulatory systems combined with *in situ* product removal system of the products provide a prospective solution to overcome the heterogeneity of lignin.

Improving the interaction between artificially designed lignin valorization modules and the endogenous metabolic networks of chassis bacteria significantly increases the marketability of lignin-based products. However, the relationships between lignin-derived metabolites, their impact on cell growth, and the connection between genotype and phenotype remain largely unexplored. This gap leaves the efficient regulation of lignin metabolic networks without adequate bioinformatic support. Fortunately, systems biology provides accurate anchors for efficient regulation, while pioneering technologies have contributed to revolutionizing metabolic regulation strategies. For example, fluorescence resonance energy transfer technology reveals the dynamic fluctuations and regulatory mechanisms of metabolic networks. These cutting-edge technologies have undoubtedly provided strong momentum for regulating lignin biological metabolic networks.

GENOME-SCALE METABOLIC MODELS PREDICT REGULATORY TARGETS FOR LIGNIN METABOLISM

With the rapid advancement of sequencing technology, whole-genome sequencing has been completed for thousands of species, elevating metabolic regulation from localized networks to a global level.[96,97] Genome-scale metabolic network reconstruction, derived from genome annotation and the cataloging of all metabolic reactions in a target organism, enables transformation into mathematical models suitable for computational analysis.[98] Consequently, genome-scale metabolic models (GEMs) have emerged as a powerful tool for systematically analyzing cellular metabolic properties. By adjusting the stoichiometric matrix, GEMs can simulate gene editing and effectively predict effective strategies for systems metabolic engineering.[98] The reconstruction of GEMs for ligninolytic microbes facilitates the integration of lignin metabolism knowledge and enhances the roadmap for lignin biological valorization.

Next-generation sequencing technology has been extensively applied to the whole-genome sequencing of ligninolytic microbes, generating a wealth of high-quality data on genes and pathways associated with lignin metabolism. This provides a valuable foundation for reconstructing genome-scale metabolic networks.[99] White-rot fungi act as lignin utilizers in nature and they can metabolize lignin macromolecules through the cooperative interaction of carbohydrate-active enzymes and "auxiliary activity" enzymes.[100,101] Therefore, white-rot fungi hold unique technological potential for lignin depolymerization due to their extracellular enzymes, which could enable industrial-scale lignin bioprocessing to yield highly bioavailable derivatives. Benefiting from large-scale genome sequencing programs, the genomes of hundreds of white-rot fungi have been sequenced and are publicly available on the fungal genome database MycoCosm.[102] System biology can predict lignin degradation phenotypes based on information contained in the genomes of white-rot fungi.[101] *Cerrena unicolor* exhibits exceptional lignin-degrading and aromatic pollutant-removal capabilities, making it an ideal biocatalyst for lignin valorization and environmental remediation.[103] The 42.79 Mb genome of *C. unicolor* SP02 was sequenced, assembled and annotated, and it was found to encode multiple oxidoreductases and lignin-degrading auxiliary enzymes. Among them, lignin-oxidizing enzymes mainly include laccase, lignin peroxidase, and manganese peroxidase. Additionally, ligninolytic auxiliary enzymes include hydrogen peroxide-producing enzymes, such as glyoxal oxidase, pyranose oxidase, vanillyl alcohol oxidase, and benzoquinone reductases. Moreover, the genome of *C. unicolor* SP02 can also encode glutathione *S*-transferase, which cleaves the β-*O*-4 bond that is abundant in the lignin polymer structure.[103] However, the highly complex genetic structure of eukaryotes poses a serious challenge for accurate gene annotation. There is still a lack of high-quality datasets for non-specific enzymatic reactions, which poses a risk of non-robustness in the construction of GEMs.[104] Fortunately, the development of high-throughput platforms for characterizing enzyme-substrate catalytic mechanisms, coupled with advances in multi-omics technologies, holds great promise for unlocking the full potential of white-rot fungi in lignin valorization.

P. putida KT2440 possesses versatile metabolic capabilities and exhibits strong robustness. Its irreplaceable metabolic performance toward aromatics makes it a high-value biocatalyst widely used in lignin bio-upgrading.[105,106] The 6.18 Mb genome of *P. putida* KT2440 was sequenced, revealing its various pathways for metabolizing multiple lignin-derived aromatics.[107] Its genome contains a considerable number of genes encoding proteins related to aromatics transport, absorption, and metabolism. In addition, hundreds of genomic islands were found in the genome of KT2440. Genome streamlining through the deletion of these genomic islands has emerged as a promising strategy for developing industrially optimized chassis strains, enhancing the biological conversion of lignin into PHA.[108,109] What is more, the whole genome sequencing of *Sphingobium* sp. SYK-6 helped to discover more microbial metabolic systems of lignin-derived biaryls.[110] The genome of *Rhodococcus* sp. RHA1 is one of the largest bacterial genomes ever reported to have been sequenced, approximately 9.8 Mb.[111] The RHA1 genome contains genes encoding 203 oxygenases, many of which are predicted to play roles in the biological metabolism of aromatic compounds. Additionally, it includes at least 26 peripheral pathways and 8 central pathways dedicated to aromatic compound metabolism.[111] These genomic data serve as a rich resource for understanding lignin microbial metabolism and form a foundational basis for constructing GEMs.

With the growing enthusiasm for lignin biological valorization, many GEMs of ligninolytic microbes have been constructed, especially *P. putida* KT2440 (Table S4). The first GEM of *P. putida* KT2440, *i*JN746, was constructed using the constraint-based reconstruction and analysis approach.[112] Notably, *i*JN746 categorizes the 950 included reactions into 55 functional subsystems, with the aromatic acid metabolism pathway being among the 3 subsystems containing the highest number of reactions. Meanwhile, iJN746 also demonstrated the utility of identifying new substrates to be used for PHA production.[112] Additionally, the *i*JP815 model drove the re-annotation of 16 genes.[113] Moreover, the model was also used to predict possible metabolic regulation strategies for expanding the acetyl coenzyme A precursor pool to promote PHA production. Six regulation strategies were proposed through the OptKnock algorithm. Therefore, *i*JP815 shows the potential for industrial application in promoting biomanufacturing.[113] Furthermore, the PpuMBEL1071 model refined the metabolism of aromatics into five categories and predicted the biomass formation rate of different aromatics.[114] Thus, the model provides guidance for the development of metabolic regulation strategies for lignin-derived aromatics as a cheap carbon source or for conversion into high-value chemicals.[114] However, since the metabolic network reconstruction process is not standardized, comparisons between GEMs from multiple species cannot be made.[115] To overcome this difficulty, a novel metabolic network reconciliation process was used to construct models without non-biological differences between *P. aeruginosa* and *P. putida*, i.e., *i*MO1086 and *i*JP962. This strategy facilitates the comparison and analysis of GEMs from different ligninolytic microbes, providing new insights into the standardized entire lignin metabolic process.[115] Subsequently, two consensus models, *i*EB1050 and PpuQY1140, were published based on the new annotation information of the genome and the reactions in previous GEMs.[116,117] The expanded *i*JN1462 model simulates the metabolism in detail, capturing a wider range of carbon and nitrogen sources than other previous models.[118] Moreover, 82 GEMs of *P. putida* strains could be generated using *i*JN1462 as a template, demonstrating the powerful metabolic functional core of this species toward lignin-derived aromatics.[118] The upgraded GEM was developed by constraining *i*JN1462 using proteomic and kinetic data, and the industrial potential of the pyruvate-overproducing KT2440 strain to produce high-value chemicals was predicted.[119]

Notably, oleaginous yeasts can efficiently convert lignin into microbial oils, positioning them as ideal chassis microbes for producing high-value chemicals and biofuels.[120] Among these, *Rhodosporidium toruloides* stands out for its ability to accumulate lipids using diverse lignin-derived aromatic compounds, including *p*-coumaric acid, *p*-hydroxybenzoic acid, ferulic acid, and vanillic acid. A high-quality GEM of *R. toruloides* was constructed based on accessible genome sequences, gene models, and gene annotations, combined with multi-omics analysis.[121] The draft metabolic network was reconstructed using orthologous protein groups and existing metabolic models. However, the initial model failed to predict the growth on *p*-coumaric acid. Multi-omics analysis showed that *p*-coumaric acid induced the upregulation of the expression of the long-chain acyl-CoA synthetase ACSL, multifunctional enzyme FOX2, and 3-ketoacyl-CoA thiolase POT1. Further analysis suggested that *p*-coumaric acid is first converted to protocatechuic acid in the peroxisome via a β-oxidation-like pathway, after which protocatechuic acid is translocated to the cytosol for further degradation through the 3-oxoadipate pathway. This study provides

the first elucidation of lignin-derived aromatic monomer metabolism in *R. toruloides*.[121]

The manual curation of genome-scale metabolic reconstructions, encompassing genome annotation, model refinement, and correction, is a time-intensive and laborious process. To address this challenge, automated reconstruction tools have been developed and employed for constructing GEMs of ligninolytic microbes.[122,123] CarveMe is an efficient automated genome-scale reconstruction tool.[124] CarveMe is based on Python and uses the BiGG database to build a universal model. Starting with this model, GEMs of single-species and microbial consortiums are constructed using a top-down approach.[124] CarveMe was used to create the first genome-wide annotation-based GEM of *R. opacus* PD630, iGR1773.[125] *R. opacus* PD630 converts heterogeneous lignin-derived aromatics into triacylglycerol via a "biological funnel."[126] The iGR1773 model contains approximately 3,000 reactions and 2,000 metabolites and was validated by growth rate and flux predictions. Substrate utilization patterns of *R. opacus* PD630 were predicted, and the results indicate that the strain metabolized glucose and phenol at similar relative ATP maintenance costs.[125] Therefore, this model provides a powerful tool for revealing metabolic rules of the aromatic substrate, thereby predicting potential targets for metabolic regulation to improve substrate utilization. Additionally, the Seed model, which enables high-throughput and automated construction, was also applied to obtain GEMs of member bacteria within the coastal lignin-degrading consortium.[127,128] Compared with the lignin degrader *Pluralibacter gergoviae* alone, the consortium containing three other non-ligninolytic microbes, namely *Vibrio alginolyticus*, *Aeromonas hydrophila*, and *Shewanella putrefaciens*, promoted lignin degradation. To investigate the complex mechanism of lignin degradation by multi-species interactions, four single-species metabolic network models and seven multi-species combination models were constructed and validated.[128] Flux balance analysis simulations indicated that the four-species consortium achieved optimal growth performance in lignin-containing media. These predictions were further validated through co-culture experiments, demonstrating the effectiveness of the modeling approach in capturing the complex dynamics of the lignin-degrading community. The ligninolytic strain *P. gergoviae* plays a central role by breaking down lignin and supplying succinic and malic acids to support the growth of other strains. In return, non-ligninolytic microbes contribute metabolites such as glycerol, alanine, and aspartic acid, which promote the growth and lignin-degrading activity of *P. gergoviae*.[128] This study highlights the potential of genome-scale metabolic modeling to guide the rational design and synthetic assembly of microbial consortia for efficient lignin valorization.

GEM, as a knowledge-integrated intelligent tool, has been widely used for microbial understanding, enzyme function prediction, drug development, and especially metabolic regulation.[129,130] Based on the high-quality GEM *i*JN1462, the EMA and Opt approaches were combined to predict the key regulatory targets of the KT2440 endogenous metabolites degradation pathway and the central carbon metabolic network.[131] After prediction, eight priority gene targets were obtained, and they were knocked out individually or in combination to enhance the metabolic flux for synthesizing isoprenoid alcohols via the heterologous mevalonate pathway. Metabolic regulation guided by genome-scale metabolic modeling and rational pathway optimization resulted in a 10-fold increase in the titer of isoprenol. Moreover, the engineered strain could produce isoprenol from sorghum hydrolysate at a titer of 432 mg/L. It is noteworthy that the engineered strain showed enhanced growth rates in media with additional sorghum hydrolysate. This result suggests that glucose and various aromatic compounds in the hydrolysate can serve as an additional source of energy supply for KT2440. Therefore, GEMs provide a guiding regulatory strategy for lignin valorization to sustainable aviation fuel and its precursor.[131] Combining the *i*JN1462 model of KT2440 with the minimal cut set approach, a workflow that can be scaled to any carbon source, chassis strains, and target high-value chemicals was developed.[132] The process allows for the prediction of minimal sets of reactions for elimination, enabling a strong coupling of product production and cell growth. Taking indigoidine as an example, 14 target genes were knocked out, successfully shifting the production from the stationary phase to the exponential phase, with the titer reaching 25.6 g/L. Therefore, this workflow provides a pre-emptive decision point for screening suitable chassis strain-chemical pairs to promote lignin bio-upgrading.[132] It is worth mentioning that GEMs of non-ligninolytic microbes also have a great potential to guide metabolic regulation of chassis strain for lignin biological valorization. A cipher of evolutionary design (CiED) was developed by extending the *i*JR904 model of *E. coli*. Gene targets were predicted and experimentally validated using CiED to redirect carbon flux toward malonyl-CoA synthesis. Model-directed metabolic engineering resulted in an astonishing increase in the production of naringenin and eriodictyol from *p*-coumaric acid and caffeic acid by more than 660% and 420%, respectively.[133]

The complementary relationship between computer science and bioinformatics makes GEMs a powerful digital tool to guide metabolic engineering. GEMs developed for ligninolytic microbes effectively describe and simulate lignin biological metabolic processes on a global scale, offering researchers valuable insights into the mechanisms of lignin metabolism and enabling the identification of key metabolic nodes for regulation (Figure 4). While GEMs have been extensively applied in metabolic regulation, their potential in lignin valorization remains underexplored. The cellular processes of most ligninolytic microbes are extremely complex, and gaps in the microbial metabolic networks involved in lignin degradation pose significant challenges for genome-scale metabolic modeling. Although various reconstruction tools have been developed, manual curation remains essential for specific steps, particularly the metabolism of lignin-derived aromatic compounds. Fortunately, the availability of comprehensive multi-omics datasets provides a robust foundation for enhancing model quality. Furthermore, the development of advanced toolkits and improvements in computational power are accelerating the high-throughput construction of metabolic models. Integrating high-quality datasets into multi-constraint models will improve the precision of metabolic regulation predictions, thereby promoting the broader application of GEMs in lignin valorization.

MACHINE LEARNING SIMULATES THE BIOLOGICAL METABOLISM REGULATION OF LIGNIN

Machine learning, as a sub-discipline of artificial intelligence, has diverse application scenarios, such as natural language processing, image recognition and healthcare.[134,135] With its powerful data-driven learning and prediction capabilities, it holds the promise of addressing the technical barriers in the biological metabolism regulation of lignin.[136] The exponentially growing omics data can provide more training datasets for machine learning and obtain more accurate learning functions to predict new outputs.[137] Machine learning can statistically correlate complex relationships in biological lignin metabolism, such as promoter selection and target product yield, enzyme sequence, and catalytic function.[138] Therefore, machine learning has a wide range of uses in processing omics datasets, protein engineering, pathway prediction, and optimization for lignin valorization.

The booming genomic revolution has led to a rapid decline in sequencing costs, generating a large amount of transcriptome, proteome, and metabolome data.[139] While valuable for metabolic regulation, the sheer volume can overwhelm metabolic engineers. Machine learning offers crucial support for analyzing these data, with independent component analysis (ICA), an unsupervised algorithm for signal separation, widely used to extract independent components from mixed variables.[140] In the field of metabolic engineering, ICA has been successfully applied to RNA-seq datasets and RB-TnSeq datasets.[141,142] By employing ICA on the *P. putida* KT2440 RNA-seq datasets, 84 groups of iModulons, namely independently modulated genes, were identified. Analysis of the biological information embedded in the iModulons allowed the genome-scale transcriptional regulatory network of KT2440 to be resolved, the transcriptome changes when it utilizes lignin derivatives as a carbon source.[141] Taken together, iModulon has undoubtedly contributed strongly to the identification of the boundaries of genes required for lignin microbial metabolism and the discovery of new regulators.[141] ICA also provides an automated method for analyzing RB-TnSeq fitness data to facilitate the analysis and prediction of gene modules in KT2440.[142] ICA identified 84 functional modules, or fModules, in which lignin metabolism-related fModules, such as fModules_28 and fModule_20, contain gene members involved in microbe-specific metabolic functions. These gene members are involved in metabolic processes such as *O*-demethylation, regeneration of multiple cofactors and aromatic transport. Therefore, this method facilitates the discovery of relationships between functional genes involved in the microbial metabolism of lignin. Moreover, combined with iModulon data, it has the potential to reveal the transcriptional regulatory mechanism of functional gene module expression.[142]

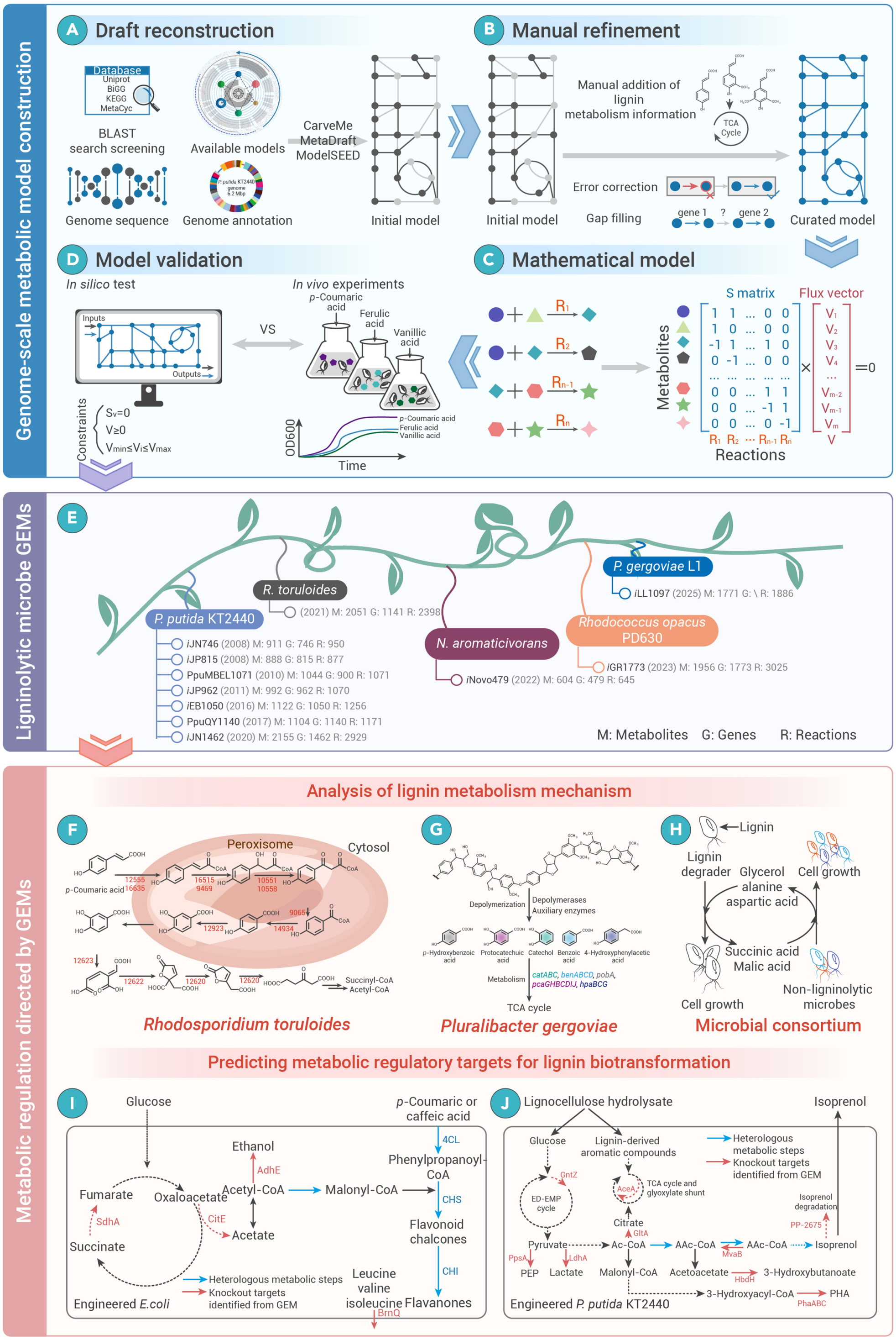

Figure 4. Genome-scale metabolic models efficiently guide metabolic regulation for lignin biological valorization (A) Initial models were drafted using genome sequence data and various bioinformatics tools. (B) The models were manually refined by identifying and adding missing reactions by means of literature checking, homologous gene matching, etc., and ensuring the accuracy of the information for each reaction and metabolite. (C) Converting curated models to mathematical models using software packages. (D) Experimental data were used to validate the predictive power and accuracy of the model. (E) Currently constructed genome-scale metabolic reconstructions of ligninolytic microbes. (F) GEMs can be applied to resolve the intracellular metabolic mechanisms of lignin-derived monomers in yeast, such as *R. toruloides*. (G) According to GEMs, ligninolytic bacteria depolymerize lignin polymer macromolecules into monomers via depolymerases and auxiliary enzymes, followed by aromatic ring-opening degradation. (H) For lignin-degrading consortium, ligninolytic specie is responsible for metabolizing lignin and providing nutrients to other species. At the same time, other non-ligninolytic species provide ligninolytic specie with small molecules needed for growth, promoting its accelerated growth and lignin metabolism. (I) By the utilization of GEMs, carbon fluxes were successfully redirected to malonyl-CoA to promote the conversion of lignin-derived monomers into high-value flavonoids. (J) The design and optimization of engineered strains by GEMs can enable the valorization of lignocellulosic hydrolysates into isoprenol. 4CL, 4-coumarate CoA ligase; AceA, isocitrate lyase; *adhAB*, alcohol dehydrogenase genes; AdhE, bifunctional aldehyde-alcohol dehydrogenase; *benABC*, benzoate 1,2-dioxygenase genes; *benD*, benzoate 1,2-dioxygenase gene; BrnQ, branched-chain amino acid permease; *cat*, catalase gene; *catA*, catechol 1,2-dioxygenase gene; *catB*, *cis*,*cis*-muconate cycloisomerase gene; *catC*, muconate cycloisomerase gene; CitE, citrate lyase; CHS, chalcone synthase; CHI, chalcone isomerase; *dypAB*, lignin oxidase genes; *dld*, dihydrolipoamide dehydrogenase gene; *hpaBC*, 4-hydroxyphenylacetate 3-monooxygenase gene; HbdH, 3-hydroxybutyrate dehydrogenase; *hpaG*, 4-hydroxyphenylacetate degradation bifunctional isomerase/decarboxylase gene; GltA, citrate synthase; GntZ, 6-phosphogluconate dehydrogenase; *gox*, glycolate oxidase gene; *gpx*, glutathione peroxidase gene; LdhA, D-lactate dehydrogenase; *mdlC*, benzoylformate decarboxylase gene; MvaB, hydroxymethylglutaryl-CoA lyase; *nuoEFG*, quinone reductase genes; *pobA*, *p*-hydroxybenzoate hydroxylase gene; *pcaHG*, protocatechuate 3,4-dioxygenase gene; *pcaB*, 3-carboxy-*cis*,*cis*-muconate cycloisomerase gene; *pcaC*, 4-carboxymuconolactone decarboxylase gene; *pcaD*, 3-oxoadipate enol-lactonase gene; *pcaIJ*, 3-oxoadipate CoA-transferase gene; PhaA, poly(3-hydroxyalkanoate) polymerase 1; PhaB, poly(3-hydroxyalkanoate) depolymerase; PhaC, poly(3-hydroxyalkanoate) polymerase; PpsA, phosphoenolpyruvate synthase; *sod*, superoxide dismutase gene; SdhA, succinate dehydrogenase; *trxB*, thioredoxin reductase gene.

Additionally, the random forest machine-learning algorithm was applied to identify and compare carbohydrate-active enzymes in white-rot fungi and brown-rot fungi.[143] For ligninolytic enzymes, machine learning models predicted the importance of the number of class II peroxidases and glyoxal oxidase for the classification of wood-rotting fungi. The glyoxal oxidase provides hydrogen peroxide to class II peroxidases to activate their ligninolytic activity. It is noteworthy that white-rot fungi have a greater number of genes encoding class II peroxidases and glyoxal oxidase than brown-rot fungi. As a result, machine learning classified wood-rotting fungi based on the number of enzyme families in the genome with 98% accuracy.[143] This presents an opportunity for machine learning to unravel the complex mechanisms of lignin metabolism and guide metabolic regulation, ushering lignin biovalorization into the era of artificial intelligence.

Machine learning-guided protein engineering aims to achieve ambitious goals, including predicting protein structures to enhance catalytic efficiency, stability, and solubility, while expanding enzyme options for pathway design and metabolic regulation.[144] Deep learning-based *de novo* protein structure prediction methods can extract biological information from massive protein sequence and structure data for downstream task applications. A web platform trRosetta based on deep learning and Rosetta was developed.[145] The combination of structure prediction performed by trRosetta and stability-design calculations performed by PROSS has led to the design of versatile peroxidases, which can efficiently decompose lignin.[146] The designed mutants can be functionally expressed in yeast and show diverse catalytic functions. Additionally, the surprising stability of these mutants under specific environmental conditions makes them effective tools for applications in the fields of microbial

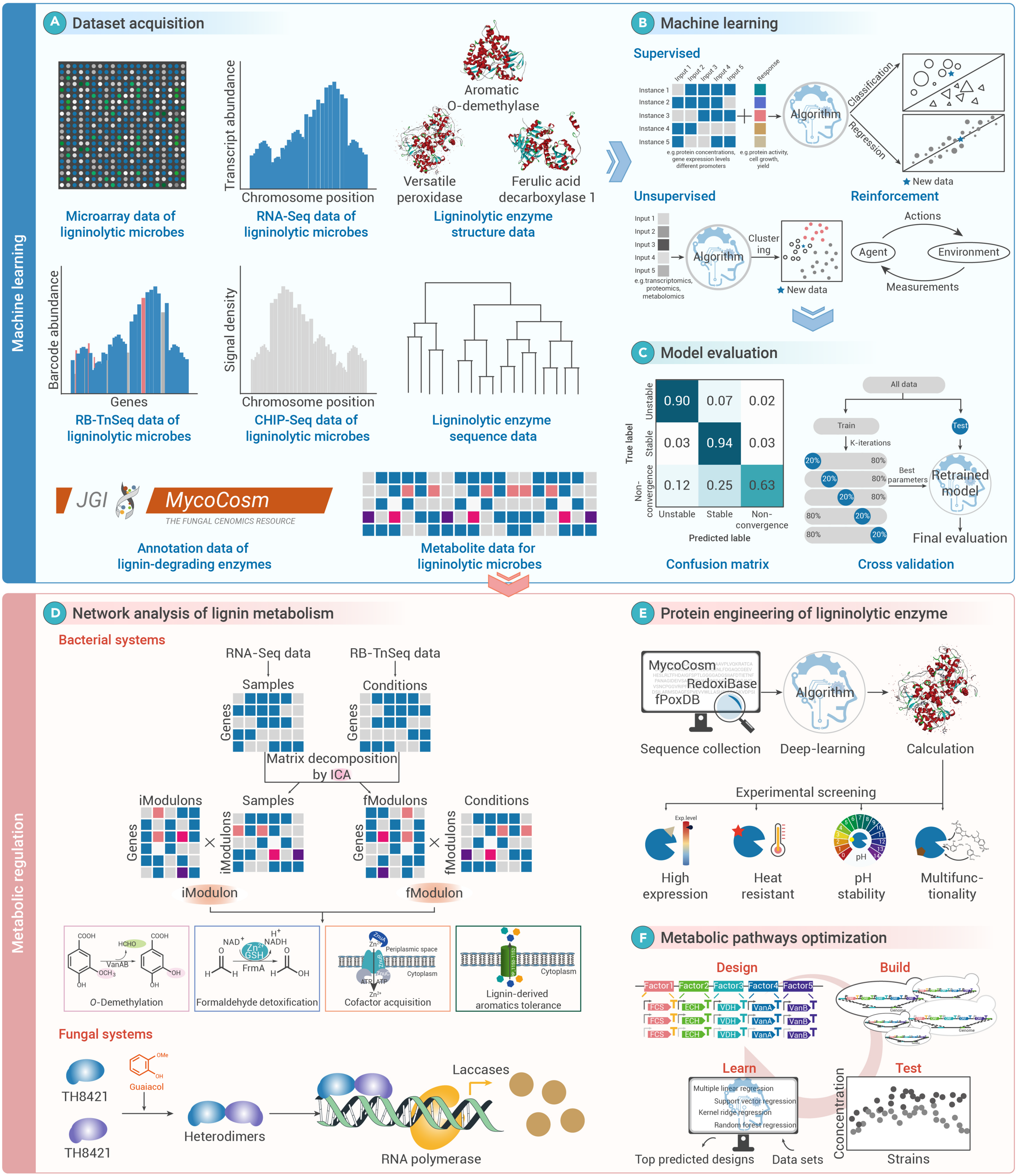

Figure 5. Machine learning brings lignin biological valorization and metabolic regulation into the era of artificial intelligence (A) The rapid development of multi-omics including genomics, metabolomics, transcriptomics, and proteomics has resulted in large datasets of high quality, providing a solid foundation for training machine learning models to mine useful biological information. (B) Computers can learn automatically based on a given dataset through different methods such as supervised learning, unsupervised learning and reinforcement learning. Multiple metabolic models can be developed based on the statistical associations contained in the dataset as a way to predict the output of other biological relationships. (C) After obtaining the metabolic model, the dataset is partitioned into multiple smaller parts and then the model is trained and validated multiple times. This confusion matrix and cross-validation process allow the generalization performance and stability of the model to be assessed. (D) Machine learning can effectively predict the interactions and regulatory relationships between genes and lignin derivatives, thus providing a powerful digital tool for elucidating the complex lignin metabolic network in bacterial and fungal

(legend continued on next page)

depolymerization of lignin, biomedicine, and paper processing. Another major player in protein engineering is deep learning-based MutCompute, which has been run to predict the amino acid sites to be optimized in poly(ethylene terephthalate) hydrolases (PETase).[147] The PETase mutant designed by MutCompute showed satisfactory robustness and catalytic activity. The performance-optimized mutant can efficiently degrade PET waste to obtain high-purity aromatic monomers, which are repolymerized to obtain new PET films.[147] Although lignin and PET have distinctly different origins, they exhibit remarkable similarities in their aromatic polymer structures and inter-monomer linkage bonds. For example, both contain a high proportion of C-O bonds.[148] Over evolutionary time, many enzyme active sites have adapted to accommodate multiple substrates, enabling them to catalyze a broad range of reactions. Notably, the PETase purified from leaf-branch compost has also been shown to degrade plant cell walls.[149] Similarly, *Tm*Fae-PETase, a lignocellulose-degrading enzyme screened from the gut microbiome of *T. molitor* larvae, also exhibited PET degradation activity.[150] These findings highlight the potential for cross-application of biocatalysts in both lignin valorization and plastic waste upcycling, suggesting that advanced strategies could be co-developed to accelerate progress in both fields. Therefore, deep learning shows its great potential in the bio-depolymerization of aromatic polymers and is a flexible tool for developing novel ligninolytic enzymes with high stability and high catalytic activity. Additionally, the computer algorithm can assist in predicting the enzymatic properties of unvalidated mutants to save time and labor. A set of ancestral sequences of phenolic acid decarboxylases was reconstructed by GRASP based on the maximum likelihood method.[151] The structural attributes of the three selected ancestors were calculated to train regression models, which enabled the prediction of the thermal stability of the remaining phenolic acid decarboxylase mutants. The obtained mutant can still catalyze ferulic acid decarboxylation efficiently at high temperatures.[151]

Microorganisms respond to the stress of environmental change by regulating gene activity. The binding of transcription factors to specific DNA sequences is one of the major gene transcriptional regulation mechanisms.[152] However, the highly dynamic and complex nature of transcriptional regulation poses a challenge in resolving microbial mechanisms in controlling ligninolytic enzyme expression. Fortunately, the powerful feature extraction advantages of machine learning make it a powerful tool for mining new transcription factors.[153] The N terminus of the transcription factor contains a sequence-specific DNA-binding domain, while the C terminus harbors a highly diverse and poorly conserved acidic activation domain (AD). By integrating high-throughput quantification of *in vivo* activity and *in vitro* interaction data with convolutional neural network training and computational modeling, the PADDLE model successfully predicted ADs in eukaryotic transcription factors.[154] Additionally, the model provided a detailed understanding of the regulatory mechanisms of AD: a large number of acidic and bulky hydrophobic residues, especially aspartic acid, tryptophan, phenylalanine, and leucine, contributed to the activation. A specific example was the white-rot fungus *Trametes hirsuta* AH28-2. The lignin-derived aromatic monomer guaiacol and the structurally similar compound *O*-toluidine triggered the enhanced expression of LacA and LacB.[155] Proteomic analysis unearthed two Zn_2Cys_6-type transcription factors, namely TH8421 and TH4300. The deep learning model predicted that the ADs in TH8421 and TH4300 were located at amino acids 1,216–1,268 and 141–193, respectively. Bioinformatics docking analysis showed that TH8421 and TH4300 activated the downstream laccase expression by forming heterodimers that directly bind to the promoters of *lacA* and *lacB*. At the same time, lignin-derived aromatic monomers promoted the formation of such heterodimers, thus realizing the regulation of laccase expression.[155] These advances will significantly enhance understanding of the regulatory mechanisms underlying lignin metabolism and inspire the development of novel biosensors and dynamic regulation strategies for lignin valorization.

The green and circular development of the bio-based economy places demands on higher titers of high-value compounds produced by microorganisms. However, the complexity of heterologous biosynthetic pathways for lignin valorization often necessitates constructing and testing numerous strains for combinatorial optimization. Fortunately, machine learning can analyze high-quality experimental datasets to uncover microbial behavioral patterns, offering an efficient and accurate approach for designing and optimizing metabolic pathways.[156] The method first requires a library of experimental units with different regulatory elements and subsequently uses phenotypic datasets to train the machine learning algorithm.[157] Machine learning-assisted optimization of *p*-coumaric acid microbial production has been achieved in *S. cerevisiae*.[157] The promoter-open reading frame-terminator cassette structure constituted a factor, and a combinatorial library was constructed using a one-pot method. Through algorithm training and machine learning, the production of *p*-coumaric acid increased by 68% within two design-build-test-learn cycles.[157] In the production of the aromatic natural product naringenin in *E. coli*, machine learning effectively mitigated the complicated epistasis by promoter optimization.[158] Based on the ProEnsemble algorithm, the expression of pathway enzymes for naringenin biosynthesis was optimized by promoter engineering. The highest titer of naringenin produced from tyrosine was achieved, which was 3.65 g/L. This record was three times higher than the fermentation production using lignin-derived *p*-coumaric acid as intermediate feeding.[158] Additionally, the combination of machine learning and multi-dimensional liquid chromatography-ion mobility spectrometry-mass spectrometry measurements enables precise metabolomic analysis of engineered bacterial strains, facilitating the prediction of key rate-limiting steps and guiding the design of targeted metabolic regulation strategies. PeakDecoder algorithm is able to achieve the differentiation of co-elution and co-mobility.[159] This high-throughput analytical and computational workflow was applied to process metabolic data for producing bisabolene from lignocellulosic hydrolysate by the engineered *R. toruloides* GB2. The results showed higher levels of IPP/DMAPP, geranyl pyrophosphate (precursor of farnesyl pyrophosphate), bisphenol (a derivative of farnesyl pyrophosphate), and HMG-CoA on high-ash, low-moisture hydrolysate. Farnesyl pyrophosphate and its derivatives induce the degradation of 3-hydroxy-3-methylglutaryl-CoA reductase, leading to a blockage of HMG-CoA conversion in the mevalonate pathway. This is consistent with the reduced levels of 3-hydroxy-3-methylglutaryl-CoA reductase observed in the proteomic analysis.[159] Unfortunately, machine learning-assisted metabolic pathway design and regulation have not yet been sufficiently exemplified in the field of lignin valorization. However, its current contribution to smart biomanufacturing has confirmed its importance in regulating microbial metabolism to promote lignin valorization.

Data-driven machine learning has become integral to all stages of systems metabolic engineering. The current accumulation of biological information on lignin biological valorization is ongoing, including changes in the transcriptome and metabolome under aromatic substrate stress, sequences and crystal structures of lignin-modifying enzymes, and libraries of regulatory elements from dominant chassis strains. These high-quality datasets serve as a powerful foundation for applying machine learning to predict metabolic regulation targets, engineer rate-limiting enzymes, and optimize metabolic pathways (Figure 5). As a result, AI-driven metabolic regulation for enhancing lignin bio-upgrading is becoming an increasingly tangible reality.

CONCLUDING REMARKS AND FUTURE PERSPECTIVES

Lignin valorization is an important part of the bio-based economic development strategy. The development of microbial cell factories to convert lignin into high-value chemicals provides a green and sustainable solution. However, the mechanical and violent introduction of artificially designed metabolic pathways into chassis strains can lead to a series of problems leading to unsatisfactory bioconversion efficiency. Intelligent and flexible metabolic regulation strategies open up bright prospects for overcoming physiological imbalances in engineered strains and improving their production performance and potential for industrial applications. The footprint of metabolic regulation is widely emerging at four levels: biotransformation critical enzymes, metabolic pathway networks, genome-phenotype, and learning prediction. However, advancing the

systems. (E) Machine learning can be used to design ligninolytic enzymes with improved performance based on sequence-structure-function relationships of proteins, such as high-expression enzymes, heat-stable enzymes, acid- or base-tolerant enzymes, and multifunctional enzymes. Compared with wild-type enzymes, these engineered enzymes are more suitable for industrial production requirements and greatly contribute to the scale-up of the valorization of lignin. (F) Machine learning can predict the optimal structure and combination of building blocks in functional gene expression cassettes. The optimized metabolic pathways designed by machine learning can effectively balance the various physiological metabolisms of the cell factory to achieve lignin bio-upgrading.

development of metabolic regulation also presents several difficulties and challenges that must be addressed.

Although the lignin-sensitive biological elements characterized so far offer an accessible toolbox for constructing regulatable genetic circuits, they fall short of the functional diversity needed for the industrial applications of lignin valorization. Developing high-throughput genomics platforms empowered by machine learning to analyze multi-omics data under varying conditions facilitates the discovery of natural regulatory elements. However, precise spatiotemporal control over the response strength of biological elements to regulate metabolic fluxes remains an area for further exploration. By combining the strengths of human expertise in identifying robust genetic traits with AI's ability to detect correlations in large datasets, intelligent control of biological elements can be achieved. While GEMs have made significant strides in metabolic regulation, the modeling of ligninolytic microbes is still constrained by limitations in biological data, computational power, and automation tools. Advancing detection technologies for metabolites, protein abundance, and other indicators, along with improved mathematical methods, will help bring predictions closer to the true metabolic state of lignin valorization. Additionally, creating a dedicated database for lignin biotransformation would also help to reduce the burden of manually managing genetic information related to lignin-derived aromatics. While machine learning has greatly assisted metabolic regulation, its practical application in lignin valorization still requires further expansion. A key challenge is synchronizing the updates of machine learning models with new genetic data on lignin metabolism. Therefore, efforts should focus on generating high-quality, standardized, and machine-readable datasets related to lignin biotransformation and developing optimization algorithms for online learning. Overall, synthetic biology-guided metabolic regulation opens new avenues to develop microbial cell factories, facilitating the industrially attractive bio-upgrading of lignin.

FUNDING AND ACKNOWLEDGMENTS

This work was supported by the National Key Research and Development Program of China (2023YFC3403500).

AUTHOR CONTRIBUTIONS

R.-Y.L., B.-Z.L., Y.-J.Y., and Z.-H.L. conceived and organized the manuscript. R.-Y.L. wrote the abstract, introduction, and the section about enzyme regulation, metabolic network regulation, genome-scale metabolic regulation, and machine learning-assisted metabolic regulation. Z.H.L. wrote the public summary and the concluding remarks and future perspectives. Z.H.L. and B.Z.L. revised the manuscript. All authors contributed to the manuscript and approved the final version.

DECLARATION OF INTERESTS

The authors declare no competing interests.

SUPPLEMENTAL INFORMATION

It can be found online at https://doi.org/10.1016/j.xinn.2025.100993.

REFERENCES

1. Liao, Y., Koelewijn, S.F., Van den Bossche, G. et al. (2020). A sustainable wood biorefinery for low–carbon footprint chemicals production. *Science* **367**:1385–1390. DOI:https://doi.org/10.1126/science.aau1567.
2. del Río, J.C., Rencoret, J., Gutiérrez, A. et al. (2020). Lignin monomers from beyond the canonical monolignol biosynthetic pathway: another brick in the wall. *ACS Sustain. Chem. Eng.* **8**:4997–5012. DOI:https://doi.org/10.1021/acssuschemeng.0c01109.
3. Bing, R.G., Sulis, D.B., Carey, M.J. et al. (2024). Beyond low lignin: Identifying the primary barrier to plant biomass conversion by fermentative bacteria. *Sci. Adv.* **10**:eadq4941. DOI:https://doi.org/10.1126/sciadv.adq4941.
4. Liu, Z.H., Liu, H., Xu, T. et al. (2025). Lignin valorization reshapes sustainable biomass refining. *Renew. Sustain. Energy Rev.* **211**:115296. DOI:https://doi.org/10.1016/j.rser.2024.115296.
5. Khan, R.J., Lau, C.Y., Guan, J. et al. (2022). Recent advances of lignin valorization techniques toward sustainable aromatics and potential benchmarks to fossil refinery products. *Bioresour. Technol.* **346**:126419. DOI:https://doi.org/10.1016/j.biortech.2021.126419.
6. Liu, Z.H., Hao, N., Wang, Y.Y. et al. (2021). Transforming biorefinery designs with 'Plug-In Processes of Lignin' to enable economic waste valorization. *Nat. Commun.* **12**:3912. DOI:https://doi.org/10.1038/s41467-021-23920-4.
7. Queneau, Y. and Han, B. (2022). Biomass: Renewable carbon resource for chemical and energy industry. *Innovation* **3**:100184. DOI:https://doi.org/10.1016/j.xinn.2021.100184.
8. Corona, A., Biddy, M.J., Vardon, D.R. et al. (2018). Life cycle assessment of adipic acid production from lignin. *Green Chem.* **20**:3857–3866. DOI:https://doi.org/10.1039/C8GC00868J.
9. Liu, H., Liu, Z.H., Zhang, R.K. et al. (2022). Bacterial conversion routes for lignin valorization. *Biotechnol. Adv.* **60**:108000. DOI:https://doi.org/10.1016/j.biotechadv.2022.108000.
10. Schutyser, W., Renders, T., Van den Bosch, S. et al. (2018). Chemicals from lignin: an interplay of lignocellulose fractionation, depolymerisation, and upgrading. *Chem. Soc. Rev.* **47**:852–908. DOI:https://doi.org/10.1039/C7CS00566K.
11. da Costa Lopes, A.M., Silvestre, A.J.D. and Coutinho, J.A.P. (2023). On the path to improve lignin depolymerization and functionalization into bio-based platform chemicals: a short review. *Curr. Opin. Green Sustain. Chem.* **43**:100850. DOI:https://doi.org/10.1016/j.cogsc.2023.100850.
12. Zhu, S.Y., Li, N., Liu, Z.H. et al. (2025). Harnessing aromatic properties for sustainable biovalorization of lignin derivatives into flavonoids. *Green Carbon*. DOI:https://doi.org/10.1016/j.greenca.2024.11.005.
13. Ge, J., Wang, G., Sui, W. et al. (2023). Highly efficient metal-acid synergetic catalytic fractionation of lignocellulose under mild conditions over lignin-coordinated N-anchoring Co single-atom catalyst. *Chem. Eng. J.* **462**:142109. DOI:https://doi.org/10.1016/j.cej.2023.142109.
14. Ma, C., Zhang, J., Yin, Y. et al. (2024). Free radical theory in lignin oxidation depolymerization. *Trends Chem.* **6**:234–247. DOI:https://doi.org/10.1016/j.trechm.2024.03.006.
15. Rahimi, A., Ulbrich, A., Coon, J.J. et al. (2014). Formic-acid-induced depolymerization of oxidized lignin to aromatics. *Nature* **515**:249–252. DOI:https://doi.org/10.1038/nature13867.
16. Liu, Z.H., Li, B.Z., Yuan, J.S. et al. (2022). Creative biological lignin conversion routes toward lignin valorization. *Trends Biotechnol.* **40**:1550–1566. DOI:https://doi.org/10.1016/j.tibtech.2022.09.014.
17. Li, F., Zhao, Y., Xue, L. et al. (2022). Microbial lignin valorization through depolymerization to aromatics conversion. *Trends Biotechnol.* **40**:1469–1487. DOI:https://doi.org/10.1016/j.tibtech.2022.09.009.
18. Liu, Z.H., Shinde, S., Xie, S. et al. (2019). Cooperative valorization of lignin and residual sugar to polyhydroxyalkanoate (PHA) for enhanced yield and carbon utilization in biorefineries. *Sustain. Energy Fuels* **3**:2024–2037. DOI:https://doi.org/10.1039/c9se00021f.
19. Duran, K., Kohlstedt, M., van Erven, G. et al. (2024). From ^{13}C-lignin to ^{13}C-mycelium: *Agaricus bisporus* uses polymeric lignin as a carbon source. *Sci. Adv.* **10**:eadl3419. DOI:https://doi.org/10.1126/sciadv.adl3419.
20. Linger, J.G., Vardon, D.R., Guarnieri, M.T. et al. (2014). Lignin valorization through integrated biological funneling and chemical catalysis. *Proc. Natl. Acad. Sci. USA* **111**:12013–12018. DOI:https://doi.org/10.1073/pnas.1410657111.
21. Erickson, E., Bleem, A., Kuatsjah, E. et al. (2022). Critical enzyme reactions in aromatic catabolism for microbial lignin conversion. *Nat. Catal.* **5**:86–98. DOI:https://doi.org/10.1038/s41929-022-00747-w.
22. Yaguchi, A.L., Lee, S.J. and Blenner, M.A. (2021). Synthetic biology towards engineering microbial lignin biotransformation. *Trends Biotechnol.* **39**:1037–1064. DOI:https://doi.org/10.1016/j.tibtech.2021.02.003.
23. Liu, L., Wang, F., Wang, L. et al. (2025). Addressing challenges in microbial manufacturing: systematic microbial biotechnology. *Innovation* **6**:100871. DOI:https://doi.org/10.1016/j.xinn.2025.100871.
24. Yu, T., Liu, Q., Wang, X. et al. (2022). Metabolic reconfiguration enables synthetic reductive metabolism in yeast. *Nat. Metab.* **4**:1551–1559. DOI:https://doi.org/10.1038/s42255-022-00654-1.
25. Nystrom, A., Papachristodoulou, A. and Angel, A. (2018). A dynamic model of resource allocation in response to the presence of a synthetic construct. *ACS Synth. Biol.* **7**:1201–1210. DOI:https://doi.org/10.1021/acssynbio.8b00015.
26. Kim, G.B., Choi, S.Y., Cho, I.J. et al. (2023). Metabolic engineering for sustainability and health. *Trends Biotechnol.* **41**:425–451. DOI:https://doi.org/10.1016/j.tibtech.2022.12.014.
27. Ko, Y.S., Kim, J.W., Lee, J.A. et al. (2020). Tools and strategies of systems metabolic engineering for the development of microbial cell factories for chemical production. *Chem. Soc. Rev.* **49**:4615–4636. DOI:https://doi.org/10.1039/d0cs00155d.
28. Chen, Y., Hu, Y., Wang, X. et al. (2022). Synergistic engineering of CRISPR-Cas nucleases enables robust mammalian genome editing. *Innovation* **3**:100264. DOI:https://doi.org/10.1016/j.xinn.2022.100264.
29. Gong, Z., Chen, J., Jiao, X. et al. (2024). Genome-scale metabolic network models for industrial microorganisms metabolic engineering: current advances and future prospects. *Biotechnol. Adv.* **72**:108319. DOI:https://doi.org/10.1016/j.biotechadv.2024.108319.
30. Sun, J., Zhu, T., Cui, Y. et al. (2025). Structure-based self-supervised learning enables ultrafast protein stability prediction upon mutation. *Innovation* **6**:100750. DOI:https://doi.org/10.1016/j.xinn.2024.100750.
31. Faulon, J.L. and Faure, L. (2021). In silico, in vitro, and in vivo machine learning in synthetic biology and metabolic engineering. *Curr. Opin. Chem. Biol.* **65**:85–92. DOI:https://doi.org/10.1016/j.cbpa.2021.06.002.
32. Liu, R.Y., Lan, H.N., Liu, Z.H. et al. (2024). Microbial valorization of lignin toward coumarins: Challenges and perspectives. *Renew. Sustain. Energy Rev.* **191**:114205. DOI:https://doi.org/10.1016/j.rser.2023.114205.
33. Liu, Z.H., Le, R.K., Kosa, M. et al. (2019). Identifying and creating pathways to improve biological lignin valorization. *Renew. Sustain. Energy Rev.* **105**:349–362. DOI:https://doi.org/10.1016/j.rser.2019.02.009.

34. Bleem, A., Kuatsjah, E., Presley, G.N. et al. (2022). Discovery, characterization, and metabolic engineering of Rieske non-heme iron monooxygenases for guaiacol *O*-demethylation. *Chem Catal.* **2**:1989–2011. DOI:https://doi.org/10.1016/j.checat.2022.04.019.
35. Wolf, M.E., Hinchen, D.J., DuBois, J.L. et al. (2022). Cytochromes P450 in the biocatalytic valorization of lignin. *Curr. Opin. Biotechnol.* **73**:43–50. DOI:https://doi.org/10.1016/j.copbio.2021.06.022.
36. Alherech, M., Omolabake, S., Holland, C.M. et al. (2021). From lignin to valuable aromatic chemicals: Lignin depolymerization and monomer separation via centrifugal partition chromatography. *ACS Cent. Sci.* **7**:1831–1837. DOI:https://doi.org/10.1021/acscentsci.1c00729.
37. Li, S., Du, L. and Bernhardt, R. (2020). Redox partners: function modulators of bacterial P450 Enzymes. *Trends Microbiol.* **28**:445–454. DOI:https://doi.org/10.1016/j.tim.2020.02.012.
38. Kovaleva, E.G. and Lipscomb, J.D. (2008). Versatility of biological non-heme Fe(II) centers in oxygen activation reactions. *Nat. Chem. Biol.* **4**:186–193. DOI:https://doi.org/10.1038/nchembio.71.
39. Notonier, S., Werner, A.Z., Kuatsjah, E. et al. (2021). Metabolism of syringyl lignin-derived compounds in *Pseudomonas putida* enables convergent production of 2-pyrone-4,6-dicarboxylic acid. *Metab. Eng.* **65**:111–122. DOI:https://doi.org/10.1016/j.ymben.2021.02.005.
40. Zhang, Z., Wang, Y., Zheng, P. et al. (2021). Promoting lignin valorization by coping with toxic C1 byproducts. *Trends Biotechnol.* **39**:331–335. DOI:https://doi.org/10.1016/j.tibtech.2020.09.005.
41. Mitsui, R., Kusano, Y., Yurimoto, H. et al. (2003). Formaldehyde fixation contributes to detoxification for growth of a nonmethylotroph, *Burkholderia cepacia* TM1, on vanillic acid. *Appl. Environ. Microbiol.* **69**:6128–6132. DOI:https://doi.org/10.1128/AEM.69.10.6128-6132.2003.
42. Nguyen, L.T., Tran, M.H. and Lee, E.Y. (2021). Co-upgrading of ethanol-assisted depolymerized lignin: A new biological lignin valorization approach for the production of protocatechuic acid and polyhydroxyalkanoic acid. *Bioresour. Technol.* **338**:125563. DOI:https://doi.org/10.1016/j.biortech.2021.125563.
43. Lee, J.Y., Sung, B.H., Oh, S.H. et al. (2019). C1 compound biosensors: design, functional study, and applications. *Int. J. Mol. Sci.* **20**:2253. DOI:https://doi.org/10.3390/ijms20092253.
44. Chen, R., Yang, S., Zhang, L. et al. (2020). Advanced strategies for production of natural products in yeast. *iScience* **23**:100879. DOI:https://doi.org/10.1016/j.isci.2020.100879.
45. Yu, T., Zhou, Y.J., Huang, M. et al. (2018). Reprogramming yeast metabolism from alcoholic fermentation to lipogenesis. *Cell* **174**:1549–1558.e14. DOI:https://doi.org/10.1016/j.cell.2018.07.013.
46. Lanfranchi, E., Trajković, M., Barta, K. et al. (2019). Exploring the selective demethylation of aryl methyl ethers with a pseudomonas rieske monooxygenase. *Chembiochem* **20**:118–125. DOI:https://doi.org/10.1002/cbic.201800594.
47. Kohler, A.C., Mills, M.J.L., Adams, P.D. et al. (2017). Structure of aryl *O*-demethylase offers molecular insight into a catalytic tyrosine-dependent mechanism. *Proc. Natl. Acad. Sci. USA* **114**:E3205–E3214. DOI:https://doi.org/10.1073/pnas.1619263114.
48. Rosini, E., D'Arrigo, P. and Pollegioni, L. (2016). Demethylation of vanillic acid by recombinant LigM in a one-pot cofactor regeneration system. *Catal. Sci. Technol.* **6**:7729–7737. DOI:https://doi.org/10.1039/c6cy01402j.
49. Cai, C., Xu, Z., Zhou, H. et al. (2021). Valorization of lignin components into gallate by integrated biological hydroxylation, *O*-demethylation, and aryl side-chain oxidation. *Sci. Adv.* **7**:eabg4585. DOI:https://doi.org/10.1126/sciadv.abg4585.
50. Zhang, R.K., Tan, Y.S., Cui, Y.Z. et al. (2021). Lignin valorization for protocatechuic acid production in engineered *Saccharomyces cerevisiae*. *Green Chem.* **23**:6515–6526. DOI:https://doi.org/10.1039/d1gc01442k.
51. Chenprakhon, P., Wongnate, T. and Chaiyen, P. (2019). Monooxygenation of aromatic compounds by flavin-dependent monooxygenases. *Protein Sci.* **28**:8–29. DOI:https://doi.org/10.1002/pro.3525.
52. Deng, Y., Zhou, Q., Wu, Y. et al. (2022). Properties and mechanisms of flavin-dependent monooxygenases and their applications in natural product synthesis. *Int. J. Mol. Sci.* **23**:2622. DOI:https://doi.org/10.3390/ijms23052622.
53. Kuatsjah, E., Johnson, C.W., Salvachúa, D. et al. (2022). Debottlenecking 4-hydroxybenzoate hydroxylation in *Pseudomonas putida* KT2440 improves muconate productivity from *p*-coumarate. *Metab. Eng.* **70**:31–42. DOI:https://doi.org/10.1016/j.ymben.2021.12.010.
54. Wang, H., Wang, L., Chen, J. et al. (2023). Promoting $FADH_2$ regeneration of hydroxylation for high-level production of hydroxytyrosol from glycerol in *Escherichia coli*. *J. Agric. Food Chem.* **71**:16681–16690. DOI:https://doi.org/10.1021/acs.jafc.3c05477.
55. Zhang, Q., Zhang, Z., Chen, Y. et al. (2023). Engineering of 4-hydroxyphenylacetate-3-hydroxylase from *Escherichia coli* for efficient biosynthesis of piceatannol. *Process Biochem.* **135**:33–39. DOI:https://doi.org/10.1016/j.procbio.2023.11.002.
56. Xin, X., Zhang, R.K., Liu, S.C. et al. (2024). Engineering yeast to convert lignocellulose into vanillin. *Chem. Eng. J.* **485**:149815. DOI:https://doi.org/10.1016/j.cej.2024.149815.
57. Kawazoe, M., Takahashi, K., Tokue, Y. et al. (2023). Catabolic System of 5-formylferulic acid, a downstream metabolite of a *β*-5-type lignin-derived dimer, in *Sphingobium lignivorans* SYK-6. *J. Agric. Food Chem.* **71**:19663–19671. DOI:https://doi.org/10.1021/acs.jafc.3c06128.
58. Marshall, S.A., Payne, K.A.P., Fisher, K. et al. (2021). UbiD domain dynamics underpins aromatic decarboxylation. *Nat. Commun.* **12**:5065. DOI:https://doi.org/10.1038/s41467-021-25278-z.
59. Del Cerro, C., Erickson, E., Dong, T. et al. (2021). Intracellular pathways for lignin catabolism in white-rot fungi. *Proc. Natl. Acad. Sci. USA* **118**:e2017381118. DOI:https://doi.org/10.1073/pnas.2017381118.
60. Payne, K.A.P., White, M.D., Fisher, K. et al. (2015). New cofactor supports α, β-unsaturated acid decarboxylation via 1, 3-dipolar cycloaddition. *Nature* **522**:497–501. DOI:https://doi.org/10.1038/nature14560.
61. Lee, H.N., Shin, W.S., Seo, S.Y. et al. (2018). Corynebacterium cell factory design and culture process optimization for muconic acid biosynthesis. *Sci. Rep.* **8**:18041. DOI:https://doi.org/10.1038/s41598-018-36320-4.
62. Johnson, C.W., Salvachúa, D., Khanna, P. et al. (2016). Enhancing muconic acid production from glucose and lignin-derived aromatic compounds via increased protocatechuate decarboxylase activity. *Metab. Eng. Commun.* **3**:111–119. DOI:https://doi.org/10.1016/j.meteno.2016.04.002.
63. Kohlstedt, M., Starck, S., Barton, N. et al. (2018). From lignin to nylon: Cascaded chemical and biochemical conversion using metabolically engineered *Pseudomonas putida*. *Metab. Eng.* **47**:279–293. DOI:https://doi.org/10.1016/j.ymben.2018.03.003.
64. Sonoki, T., Takahashi, K., Sugita, H. et al. (2017). Glucose-free *cis,cis*-muconic acid production via new metabolic designs corresponding to the heterogeneity of lignin. *ACS Sustain. Chem. Eng.* **6**:1256–1264. DOI:https://doi.org/10.1021/acssuschemeng.7b03597.
65. Varman, A.M., He, L., Follenfant, R. et al. (2016). Decoding how a soil bacterium extracts building blocks and metabolic energy from ligninolysis provides road map for lignin valorization. *Proc. Natl. Acad. Sci. USA* **113**:E5802–E5811. DOI:https://doi.org/10.1073/pnas.1606043113.
66. Grüning, N.M., Lehrach, H. and Ralser, M. (2010). Regulatory crosstalk of the metabolic network. *Trends Biochem. Sci.* **35**:220–227. DOI:https://doi.org/10.1016/j.tibs.2009.12.001.
67. Chou, A., Lee, S.H., Zhu, F. et al. (2021). An orthogonal metabolic framework for one-carbon utilization. *Nat. Metab.* **3**:1385–1399. DOI:https://doi.org/10.1038/s42255-021-00453-0.
68. Montano, L.J., Duran, L. and Avalos, J.L. (2022). Physiological limitations and opportunities in microbial metabolic engineering. *Nat. Rev. Microbiol.* **20**:35–48. DOI:https://doi.org/10.1038/s41579-021-00600-0.
69. Lee, E.G., Yoon, S.H., Das, A. et al. (2009). Directing vanillin production from ferulic acid by increased acetyl-CoA consumption in recombinant *Escherichia coli*. *Biotechnol. Bioeng.* **102**:200–208. DOI:https://doi.org/10.1002/bit.22040.
70. Manfrao-Netto, J.H.C., Lund, F., Muratovska, N. et al. (2021). Metabolic engineering of *Pseudomonas putida* for production of vanillylamine from lignin-derived substrates. *Microb. Biotechnol.* **14**:2448–2462. DOI:https://doi.org/10.1111/1751-7915.13764.
71. Johnson, C.W., Salvachúa, D., Rorrer, N.A. et al. (2019). Innovative chemicals and materials from bacterial aromatic catabolic pathways. *Joule* **3**:1523–1537. DOI:https://doi.org/10.1016/j.joule.2019.05.011.
72. Loeschcke, A. and Thies, S. (2020). Engineering of natural product biosynthesis in *Pseudomonas putida*. *Curr. Opin. Biotechnol.* **65**:213–224. DOI:https://doi.org/10.1016/j.copbio.2020.03.007.
73. Salvachua, D., Rydzak, T., Auwae, R. et al. (2020). Metabolic engineering of *Pseudomonas putida* for increased polyhydroxyalkanoate production from lignin. *Microb. Biotechnol.* **13**:290–298. DOI:https://doi.org/10.1111/1751-7915.13481.
74. Lin, L., Cheng, Y., Pu, Y. et al. (2016). Systems biology-guided biodesign of consolidated lignin conversion. *Green Chem.* **18**:5536–5547. DOI:https://doi.org/10.1039/c6gc01131d.
75. Liu, H., Tao, X., Ntakirutimana, S. et al. (2024). Engineering *Pseudomonas putida* for lignin bioconversion into *cis-cis* muconic acid. *Chem. Eng. J.* **495**:153375. DOI:https://doi.org/10.1016/j.cej.2024.153375.
76. Rorrer, N.A., Notonier, S.F., Knott, B.C. et al. (2022). Production of *β*-ketoadipic acid from glucose in *Pseudomonas putida* KT2440 for use in performance-advantaged nylons. *Cell Rep. Phys. Sci.* **3**:100840. DOI:https://doi.org/10.1016/j.xcrp.2022.100840.
77. Werner, A.Z., Cordell, W.T., Lahive, C.W. et al. (2023). Lignin conversion to *β*-ketoadipic acid by *Pseudomonas putida* via metabolic engineering and bioprocess development. *Sci. Adv.* **9**:eadj0053. DOI:https://doi.org/10.1126/sciadv.adj0053.
78. Johnson, C.W., Abraham, P.E., Linger, J.G. et al. (2017). Eliminating a global regulator of carbon catabolite repression enhances the conversion of aromatic lignin monomers to muconate in *Pseudomonas putida* KT2440. *Metab. Eng. Commun.* **5**:19–25. DOI:https://doi.org/10.1016/j.meteno.2017.05.002.
79. Gómez-Álvarez, H., Iturbe, P., Rivero-Buceta, V. et al. (2022). Bioconversion of lignin-derived aromatics into the building block pyridine 2, 4-dicarboxylic acid by engineering recombinant *Pseudomonas putida* strains. *Bioresour. Technol.* **346**:126638. DOI:https://doi.org/10.1016/j.biortech.2021.126638.
80. Zhou, K., Qiao, K., Edgar, S. et al. (2015). Distributing a metabolic pathway among a microbial consortium enhances production of natural products. *Nat. Biotechnol.* **33**:377–383. DOI:https://doi.org/10.1038/nbt.3095.
81. Zhu, S.Y., Liu, S.C., Zhang, C.X. et al. (2024). Pathway and enzyme engineering for the bioconversion of lignin derivatives into homoeriodictyol in *Saccharomyces cerevisiae*. *Green Chem.* **26**:5260–5272. DOI:https://doi.org/10.1039/d4gc00183d.
82. Yang, S.M., Shim, G.Y., Kim, B.G. et al. (2015). Biological synthesis of coumarins in *Escherichia coli*. *Microb. Cell Fact.* **14**:65. DOI:https://doi.org/10.1186/s12934-015-0248-y.
83. Zhao, C.H., Zhang, R.K., Qiao, B. et al. (2020). Engineering budding yeast for the production of coumarins from lignin. *Biochem. Eng. J.* **160**:107634. DOI:https://doi.org/10.1016/j.bej.2020.107634.

84. Wang, Z., Zhou, Y., Wang, Y. et al. (2023). Reconstitution and optimization of the marmesin biosynthetic pathway in yeast. *ACS Synth. Biol.* **12**:2922–2933. DOI:https://doi.org/10.1021/acssynbio.3c00267.
85. Karlen, S.D., Fasahati, P., Mazaheri, M. et al. (2020). Assessing the viability of recovery of hydroxycinnamic acids from lignocellulosic biorefinery alkaline pretreatment waste streams. *ChemSusChem* **13**:1922. DOI:https://doi.org/10.1002/cssc.202000820.
86. Zhao, J., Ou, S., Ding, S. et al. (2011). Effect of activated charcoal treatment of alkaline hydrolysates from sugarcane bagasse on purification of *p*-coumaric acid. *Chem. Eng. Res. Des.* **89**:2176–2181. DOI:https://doi.org/10.1016/j.cherd.2011.02.010.
87. Palme, J., Li, A. and Springer, M. (2025). The galactokinase enzyme of yeast senses metabolic flux to stabilize galactose pathway regulation. *Nat. Metab.* **7**:137–147. DOI:https://doi.org/10.1038/s42255-024-01181-x.
88. Shi, S., Ang, E.L. and Zhao, H. (2018). In vivo biosensors: mechanisms, development, and applications. *J. Ind. Microbiol. Biotechnol.* **45**:491–516. DOI:https://doi.org/10.1007/s10295-018-2004-x.
89. Alvarez-Gonzalez, G. and Dixon, N. (2019). Genetically encoded biosensors for lignocellulose valorization. *Biotechnol. Biofuels* **12**:246. DOI:https://doi.org/10.1186/s13068-019-1585-6.
90. Lo, T.M., Chng, S.H., Teo, W.S. et al. (2016). A two-layer gene circuit for decoupling cell growth from metabolite production. *Cell Syst.* **3**:133–143. DOI:https://doi.org/10.1016/j.cels.2016.07.012.
91. Elmore, J.R., Dexter, G.N., Salvachúa, D. et al. (2021). Production of itaconic acid from alkali pretreated lignin by dynamic two stage bioconversion. *Nat. Commun.* **12**:2261. DOI:https://doi.org/10.1038/s41467-021-22556-8.
92. Li, J., Yue, C., Wei, W. et al. (2022). Construction of a *p*-coumaric and ferulic acid auto-regulatory system in *Pseudomonas putida* KT2440 for protocatechuate production from lignin-derived aromatics. *Bioresour. Technol.* **344**:126221. DOI:https://doi.org/10.1016/j.biortech.2021.126221.
93. Wu, W., Liu, F. and Singh, S. (2018). Toward engineering *E. coli* with an autoregulatory system for lignin valorization. *Proc. Natl. Acad. Sci. USA* **115**:2970–2975. DOI:https://doi.org/10.1073/pnas.1720129115.
94. Zhao, Y., Xue, L., Huang, Z. et al. (2024). Lignin valorization to bioplastics with an aromatic hub metabolite-based autoregulation system. *Nat. Commun.* **15**:9288. DOI:https://doi.org/10.1038/s41467-024-53609-3.
95. Deng, C., Wu, Y., Lv, X. et al. (2022). Refactoring transcription factors for metabolic engineering. *Biotechnol. Adv.* **57**:107935. DOI:https://doi.org/10.1016/j.biotechadv.2022.107935.
96. Wang, X., Chen, N., Cruz-Morales, P. et al. (2024). Elucidation of genes enhancing natural product biosynthesis through co-evolution analysis. *Nat. Metab.* **6**:933–946. DOI:https://doi.org/10.1038/s42255-024-01024-9.
97. Gao, J., Li, Y., Yu, W. et al. (2022). Rescuing yeast from cell death enables overproduction of fatty acids from sole methanol. *Nat. Metab.* **4**:932–943. DOI:https://doi.org/10.1038/s42255-022-00601-0.
98. O'Brien, E.J., Monk, J.M. and Palsson, B.O. (2015). Using genome-scale models to predict biological capabilities. *Cell* **161**:971–987. DOI:https://doi.org/10.1016/j.cell.2015.05.019.
99. Paul, M., Pandey, N.K., Banerjee, A. et al. (2023). An insight into omics analysis and metabolic pathway engineering of lignin-degrading enzymes for enhanced lignin valorization. *Bioresour. Technol.* **379**:129045. DOI:https://doi.org/10.1016/j.biortech.2023.129045.
100. Drula, E., Garron, M.L., Dogan, S. et al. (2022). The carbohydrate-active enzyme database: functions and literature. *Nucleic Acids Res.* **50**:D571–D577. DOI:https://doi.org/10.1093/nar/gkab1045.
101. Kijpornyongpan, T., Schwartz, A., Yaguchi, A. et al. (2022). Systems biology-guided understanding of white-rot fungi for biotechnological applications: a review. *iScience* **25**:104640. DOI:https://doi.org/10.1016/j.isci.2022.104640.
102. Grigoriev, I.V., Nikitin, R., Haridas, S. et al. (2014). MycoCosm portal: gearing up for 1000 fungal genomes. *Nucleic Acids Res.* **42**:D699–D704. DOI:https://doi.org/10.1093/nar/gkt1183.
103. Zhang, Z., Shah, A.M., Mohamed, H. et al. (2021). Genomic studies of white-rot fungus *Cerrena unicolor* SP02 provide insights into food safety value-added utilization of non-food lignocellulosic biomass. *J. Fungi* **7**:835. DOI:https://doi.org/10.3390/jof7100835.
104. Meyer, V., Basenko, E.Y., Benz, J.P. et al. (2020). Growing a circular economy with fungal biotechnology: a white paper. *Fungal Biol. Biotechnol.* **7**:5. DOI:https://doi.org/10.1186/s40694-020-00095-z.
105. Salvachua, D., Werner, A.Z., Pardo, I. et al. (2020). Outer membrane vesicles catabolize lignin-derived aromatic compounds in *Pseudomonas putida* KT2440. *Proc. Natl. Acad. Sci. USA* **117**:9302–9310. DOI:https://doi.org/10.1073/pnas.1921073117.
106. Mohammad, S.H. and Bhukya, B. (2022). Biotransformation of toxic lignin and aromatic compounds of lignocellulosic feedstock into eco-friendly biopolymers by *Pseudomonas putida* KT2440. *Bioresour. Technol.* **363**:128001. DOI:https://doi.org/10.1016/j.biortech.2022.128001.
107. Nelson, K.E., Weinel, C., Paulsen, I.T. et al. (2002). Complete genome sequence and comparative analysis of the metabolically versatile *Pseudomonas putida* KT2440. *Environ. Microbiol.* **4**:799–808. DOI:https://doi.org/10.1046/j.1462-2920.2002.00366.x.
108. Liang, P., Zhang, Y., Xu, B. et al. (2020). Deletion of genomic islands in the *Pseudomonas putida* KT2440 genome can create an optimal chassis for synthetic biology applications. *Microb. Cell Fact.* **19**:70. DOI:https://doi.org/10.1186/s12934-020-01329-w.
109. Zong, Q.J., Xu, T., Liu, H. et al. (2022). Microbial valorization of lignin to bioplastic by genome-reduced *Pseudomonas putida*. *Front. Microbiol.* **13**:923664. DOI:https://doi.org/10.3389/fmicb.2022.923664.
110. Masai, E., Kamimura, N., Kasai, D. et al. (2012). Complete genome sequence of *Sphingobium* sp. strain SYK-6, a degrader of lignin-derived biaryls and monoaryls. *J. Bacteriol.* **194**:534–535. DOI:https://doi.org/10.1128/JB.06254-11.
111. McLeod, M.P., Warren, R.L., Hsiao, W.W.L. et al. (2006). The complete genome of *Rhodococcus* sp. RHA1 provides insights into a catabolic powerhouse. *Proc. Natl. Acad. Sci. USA* **103**:15582–15587. DOI:https://doi.org/10.1073/pnas.0607048103.
112. Nogales, J., Palsson, B.Ø. and Thiele, I. (2008). A genome-scale metabolic reconstruction of *Pseudomonas putida* KT2440: iJN746 as a cell factory. *BMC Syst. Biol.* **2**:79. DOI:https://doi.org/10.1186/1752-0509-2-79.
113. Puchalka, J., Oberhardt, M.A., Godinho, M. et al. (2008). Genome-scale reconstruction and analysis of the *Pseudomonas putida* KT2440 metabolic network facilitates applications in biotechnology. *PLoS Comput. Biol.* **4**:e1000210. DOI:https://doi.org/10.1371/journal.pcbi.1000210.
114. Sohn, S.B., Kim, T.Y., Park, J.M. et al. (2010). In silico genome-scale metabolic analysis of *Pseudomonas putida* KT2440 for polyhydroxyalkanoate synthesis, degradation of aromatics and anaerobic survival. *Biotechnol. J.* **5**:739–750. DOI:https://doi.org/10.1002/biot.201000124.
115. Oberhardt, M.A., Puchałka, J., Martins dos Santos, V.A.P. et al. (2011). Reconciliation of genome-scale metabolic reconstructions for comparative systems analysis. *PLoS Comput. Biol.* **7**:e1001116. DOI:https://doi.org/10.1371/journal.pcbi.1001116.
116. Belda, E., van Heck, R.G.A., José Lopez-Sanchez, M. et al. (2016). The revisited genome of *Pseudomonas putida* KT2440 enlightens its value as a robust metabolic chassis. *Environ. Microbiol.* **18**:3403–3424. DOI:https://doi.org/10.1111/1462-2920.13230.
117. Yuan, Q., Huang, T., Li, P. et al. (2017). Pathway-consensus approach to metabolic network reconstruction for *Pseudomonas putida* KT2440 by systematic comparison of published models. *PLoS One* **12**:e0169437. DOI:https://doi.org/10.1371/journal.pone.0169437.
118. Nogales, J., Mueller, J., Gudmundsson, S. et al. (2020). High-quality genome-scale metabolic modelling of *Pseudomonas putida* highlights its broad metabolic capabilities. *Environ. Microbiol.* **22**:255–269. DOI:https://doi.org/10.1111/1462-2920.14843.
119. Bujdoš, D., Popelářová, B., Volke, D.C. et al. (2023). Engineering of *Pseudomonas putida* for accelerated co-utilization of glucose and cellobiose yields aerobic overproduction of pyruvate explained by an upgraded metabolic model. *Metab. Eng.* **75**:29–46. DOI:https://doi.org/10.1016/j.ymben.2022.10.011.
120. Spagnuolo, M., Yaguchi, A. and Blenner, M. (2019). Oleaginous yeast for biofuel and oleochemical production. *Curr. Opin. Biotechnol.* **57**:73–81. DOI:https://doi.org/10.1016/j.copbio.2019.02.011.
121. Kim, J., Coradetti, S.T., Kim, Y.M. et al. (2020). Multi-omics driven metabolic network reconstruction and analysis of lignocellulosic carbon utilization in *Rhodosporidium toruloides*. *Front. Bioeng. Biotechnol.* **8**:612832. DOI:https://doi.org/10.3389/fbioe.2020.612832.
122. Thiele, I. and Palsson, B.Ø. (2010). A protocol for generating a high-quality genome-scale metabolic reconstruction. *Nat. Protoc.* **5**:93–121. DOI:https://doi.org/10.1038/nprot.2009.203.
123. Mendoza, S.N., Olivier, B.G., Molenaar, D. et al. (2019). A systematic assessment of current genome-scale metabolic reconstruction tools. *Genome Biol.* **20**:158. DOI:https://doi.org/10.1186/s13059-019-1769-1.
124. Machado, D., Andrejev, S., Tramontano, M. et al. (2018). Fast automated reconstruction of genome-scale metabolic models for microbial species and communities. *Nucleic Acids Res.* **46**:7542–7553. DOI:https://doi.org/10.1093/nar/gky537.
125. Roell, G.W., Schenk, C., Anthony, W.E. et al. (2023). A high-quality genome-scale model for *Rhodococcus opacus* metabolism. *ACS Synth. Biol.* **12**:1632–1644. DOI:https://doi.org/10.1021/acssynbio.2c00618.
126. Chatterjee, A., DeLorenzo, D.M., Carr, R. et al. (2020). Bioconversion of renewable feedstocks by *Rhodococcus opacus*. *Curr. Opin. Biotechnol.* **64**:10–16. DOI:https://doi.org/10.1016/j.copbio.2019.08.013.
127. Henry, C.S., DeJongh, M., Best, A.A. et al. (2010). High-throughput generation, optimization and analysis of genome-scale metabolic models. *Nat. Biotechnol.* **28**:977–982. DOI:https://doi.org/10.1038/nbt.1672.
128. Peng, Q., Zhao, C., Wang, X. et al. (2025). Modeling bacterial interactions uncovers the importance of outliers in the coastal lignin-degrading consortium. *Nat. Commun.* **16**:639. DOI:https://doi.org/10.1038/s41467-025-56012-8.
129. Fang, X., Lloyd, C.J. and Palsson, B.O. (2020). Reconstructing organisms in silico: genome-scale models and their emerging applications. *Nat. Rev. Microbiol.* **18**:731–743. DOI:https://doi.org/10.1038/s41579-020-00440-4.
130. Gu, C., Kim, G.B., Kim, W.J. et al. (2019). Current status and applications of genome-scale metabolic models. *Genome Biol.* **20**:121. DOI:https://doi.org/10.1186/s13059-019-1730-3.
131. Banerjee, D., Yunus, I.S., Wang, X. et al. (2024). Genome-scale and pathway engineering for the sustainable aviation fuel precursor isoprenol production in *Pseudomonas putida*. *Metab. Eng.* **82**:157–170. DOI:https://doi.org/10.1016/j.ymben.2024.02.004.
132. Banerjee, D., Eng, T., Lau, A.K. et al. (2020). Genome-scale metabolic rewiring improves titers rates and yields of the non-native product indigoidine at scale. *Nat. Commun.* **11**:5385. DOI:https://doi.org/10.1038/s41467-020-19171-4.
133. Fowler, Z.L., Gikandi, W.W. and Koffas, M.A.G. (2009). Increased malonyl coenzyme A biosynthesis by tuning the *Escherichia coli* metabolic network and its application to flavanone production. *Appl. Environ. Microbiol.* **75**:5831–5839. DOI:https://doi.org/10.1128/AEM.00270-09.

134. Esteva, A., Robicquet, A., Ramsundar, B. et al. (2019). A guide to deep learning in healthcare. *Nat. Med.* **25**:24–29. DOI:https://doi.org/10.1038/s41591-018-0316-z.
135. Xu, Y., Liu, X., Cao, X. et al. (2021). Artificial intelligence: a powerful paradigm for scientific research. *Innovation* **2**:100179. DOI:https://doi.org/10.1016/j.xinn.2021.100179.
136. Konno, N. and Iwasaki, W. (2023). Machine learning enables prediction of metabolic system evolution in bacteria. *Sci. Adv.* **9**:eadc9130. DOI:https://doi.org/10.1126/sciadv.adc9130.
137. Patra, P., Disha, B., Kundu, P. et al. (2023). Recent advances in machine learning applications in metabolic engineering. *Biotechnol. Adv.* **62**:108069. DOI:https://doi.org/10.1016/j.biotechadv.2022.108069.
138. Lawson, C.E., Martí, J.M., Radivojevic, T. et al. (2021). Machine learning for metabolic engineering: A review. *Metab. Eng.* **63**:34–60. DOI:https://doi.org/10.1016/j.ymben.2020.10.005.
139. Stephens, Z.D., Lee, S.Y., Faghri, F. et al. (2015). Big Data: Astronomical or genomical? *PLoS Biol.* **13**:e1002195. DOI:https://doi.org/10.1371/journal.pbio.1002195.
140. Liebermeister, W. (2002). Linear modes of gene expression determined by independent component analysis. *Bioinformatics* **18**:51–60. DOI:https://doi.org/10.1093/bioinformatics/18.1.51.
141. Lim, H.G., Rychel, K., Sastry, A.V. et al. (2022). Machine-learning from *Pseudomonas putida* KT2440 transcriptomes reveals its transcriptional regulatory network. *Metab. Eng.* **72**:297–310. DOI:https://doi.org/10.1016/j.ymben.2022.04.004.
142. Borchert, A.J., Bleem, A.C., Lim, H.G. et al. (2024). Machine learning analysis of rb-tnseq fitness data predicts functional gene modules in *Pseudomonas putida* KT2440. *mSystems* **9**:e0094223. DOI:https://doi.org/10.1128/msystems.00942-23.
143. Hasegawa, N., Sugiyama, M. and Igarashi, K. (2024). Random forest machine-learning algorithm classifies white- and brown-rot fungi according to the number of the genes encoding carbohydrate-active enzyme families. *Appl. Environ. Microbiol.* **90**:e0048224. DOI:https://doi.org/10.1128/aem.00482-24.
144. Kouba, P., Kohout, P., Haddadi, F. et al. (2023). Machine learning-guided protein engineering. *ACS Catal.* **13**:13863–13895. DOI:https://doi.org/10.1021/acscatal.3c02743.
145. Du, Z., Su, H., Wang, W. et al. (2021). The trRosetta server for fast and accurate protein structure prediction. *Nat. Protoc.* **16**:5634–5651. DOI:https://doi.org/10.1038/s41596-021-00628-9.
146. Barber-Zucker, S., Mindel, V., Garcia-Ruiz, E. et al. (2022). Stable and functionally diverse versatile peroxidases designed directly from sequences. *J. Am. Chem. Soc.* **144**:3564–3571. DOI:https://doi.org/10.1021/jacs.1c12433.
147. Lu, H., Diaz, D.J., Czarnecki, N.J. et al. (2022). Machine learning-aided engineering of hydrolases for PET depolymerization. *Nature* **604**:662–667. DOI:https://doi.org/10.1038/s41586-022-04599-z.
148. Lee, K., Jing, Y., Wang, Y. et al. (2022). A unified view on catalytic conversion of biomass and waste plastics. *Nat. Rev. Chem* **6**:635–652. DOI:https://doi.org/10.1038/s41570-022-00411-8.
149. Sulaiman, S., Yamato, S., Kanaya, E. et al. (2012). Isolation of a novel cutinase homolog with polyethylene terephthalate-degrading activity from leaf-branch compost by using a metagenomic approach. *Appl. Environ. Microbiol.* **78**:1556–1562. DOI:https://doi.org/10.1128/AEM.06725-11.
150. Mamtimin, T., Ouyang, X., Wu, W.M. et al. (2024). Novel feruloyl esterase for the degradation of polyethylene terephthalate (PET) screened from the gut microbiome of plastic-degrading mealworms (*Tenebrio Molitor* Larvae). *Environ. Sci. Technol.* **58**:17717–17731. DOI:https://doi.org/10.1021/acs.est.4c01495.
151. Myrtollari, K., Calderini, E., Kracher, D. et al. (2024). Stability increase of phenolic acid decarboxylase by a combination of protein and solvent engineering unlocks applications at elevated temperatures. *ACS Sustain. Chem. Eng.* **12**:3575–3584. DOI:https://doi.org/10.1021/acssuschemeng.3c06513.
152. Zheng, A., Lamkin, M., Zhao, H. et al. (2021). Deep neural networks identify sequence context features predictive of transcription factor binding. *Nat. Mach. Intell.* **3**:172–180. DOI:https://doi.org/10.1038/s42256-020-00282-y.
153. Kim, G.B., Gao, Y., Palsson, B.O. et al. (2021). DeepTFactor: a deep learning-based tool for the prediction of transcription factors. *Proc. Natl. Acad. Sci. USA* **118**:e2021171118. DOI:https://doi.org/10.1073/pnas.2021171118.
154. Sanborn, A.L., Yeh, B.T., Feigerle, J.T. et al. (2021). Simple biochemical features underlie transcriptional activation domain diversity and dynamic, fuzzy binding to mediator. *eLife* **10**:e68068. DOI:https://doi.org/10.7554/eLife.68068.
155. Wang, C., Zhang, X., Wu, K. et al. (2024). Two Zn_2Cys_6-type transcription factors respond to aromatic compounds and regulate the expression of laccases in the white-rot fungus *Trametes hirsuta*. *Appl. Environ. Microbiol.* **90**:e0054524. DOI:https://doi.org/10.1128/aem.00545-24.
156. Zhang, J., Petersen, S.D., Radivojevic, T. et al. (2020). Combining mechanistic and machine learning models for predictive engineering and optimization of tryptophan metabolism. *Nat. Commun.* **11**:4880. DOI:https://doi.org/10.1038/s41467-020-17910-1.
157. Moreno-Paz, S., van der Hoek, R., Eliana, E. et al. (2024). Machine learning-guided optimization of *p*-coumaric acid production in Yeast. *ACS Synth. Biol.* **13**:1312–1322. DOI:https://doi.org/10.1021/acssynbio.4c00035.
158. Deng, H., Yu, H., Deng, Y. et al. (2024). Pathway evolution through a bottlenecking-debottlenecking strategy and machine. *Adv. Sci.* **11**:2306935. DOI:https://doi.org/10.1002/advs.202306935.
159. Bilbao, A., Munoz, N., Kim, J. et al. (2023). PeakDecoder enables machine learning-based metabolite annotation and accurate profiling in multidimensional mass spectrometry measurements. *Nat. Commun.* **14**:2461. DOI:https://doi.org/10.1038/s41467-023-37031-9.

The Innovation, Volume *6*

Supplemental Information

Multiscale metabolic engineering in biological lignin valorization

Ruo-Ying Liu, Bing-Zhi Li, Ying-Jin Yuan, and Zhi-Hua Liu

Table S1 Lignocellulose fractionation and lignin depolymerization yield aromatic derivatives suitable for microbial metabolism

Strategies	Lignocellulose source or lignin type	System specifications or catalysts	Reaction temperature (℃)	Reaction time	Cleavage of linkages	Aromatic derivatives	Refs
Alkaline pretreatment	Sugarcane	NaOH (9% ww)	120	60 h	*β*-*O*-4	Phenol, guaiacol, 4-methylphenol, 4-methyl guaiacol, vanillin	S1
	Corn stover	50% Ethanol with 1% NaOH	120	80 h	*β*-*O*-4 and *β*-5	4-Hydroxybenzaldehyde, coumarone, *p*-coumaric acid, ferulic acid	S2
Acid pretreatment	Poplar chips	0.05 % (w/w) sulfuric acid	160-280	2-10 min	*β*-*O*-4	Vanillin, benzaldehyde, hydroxybenzaldehyde, 2-methoxy-4-vinylphenol, 2,6-dimethoxy-phenol, 4-hydroxy-3,5-dimethoxy-benzaldehyde, benzoic acids.	S3
	Alkali lignin	1.5 M hydrochloric acid with microwave heating	100	1 h	*β*-*O*-4	Phenol, Catechol, Isoeugenol, Eugenol, Vanillin, 2,6-dimethoxyphenol	S4
Extractive-ammonia pretreatment	Corn stover	Ammonia	120	30 min	Ester bonds	Low molecular weight lignin	S5
Organosolv pretreatment	Sorghum stem	1.90% NaOH with 70% ethanol	69.8	1 h	Ester bonds	*p*-Coumaric acid, ferulic acid	S6
	Rice straw	Ethanol/water mixture of 60.5% (w/w) ethanol concentration	30	1 h	Ester bonds	Tricin, vanillin, ferulic acid and *p*-coumaric acid	S7
Catalytic oxidative depolymerization	Aspen lignin	4-acetamido-TEMPO-initiated oxidation	Oxidation at 65℃, depolymerization at 110℃	Oxidation for 48 h	C-O bond	4-hydroxybenzoic acid, vanillin, vanillic acid, syringaldehyde, syringic acid	S8

	β-*O*-4 Linkages lignin model compounds	Heterogeneous cobalt nanoparticles as catalyst with dioxygen as the oxidant	150	24 h	C−C bond	Corresponding aromatic esters	S9
Catalytic reductive depolymerization	Poplar	Cobalt monatomic catalyst (CoSAs-N@LC)	160	1 h	C_β-C_γ, *β*-*O*-4	Methylparaben, *β*-hydroxypropiovanillone and acetovanillone	S10
	Soda lignin	Bimetallic $Ru_{0.6}Ni_{0.4}$ catalyst	350	40 min	*β*-*O*-4, *β*-5 and *β*-*β*	Guaiacol, 4-ethylphenol, 4-methylguaiacol, syringol	S11
Biological depolymerization	Lignin model compounds containing *β*-ether bonds	Versatile peroxidase from *B. adusta*	25	/	*β*-*O*-4	Guaiacol	S12
	Model dimeric lignin	Lignin peroxidase from *P. chrysosporium*	25	/	*β*-*O*-4	Guaiacol	S13
	Alkali lignin	DyP-type peroxidase from *Bacillus sp. Strain* BL5	35	20 min	*α*, *β* and *β*-ether bonds	Vanillin, 2-methyoxyphenol, *p*-hydroxybenzoic acid	S14
	Kraft lignin	Laccase from *Bacillus* sp. PCH94	50	Overnight	C−C bond	Ferulic acid, acetovanillone, dimers, trimer	S15
	Industry alkaline lignin	Laccase and manganese peroxidase from *L. betulina* and *T. versicolor*	30	168-192 h	Side-chain cleavage	4-Hydroxybenzoic acid, benzoic acid, phenylacetic acid	S16
	Alkaline lignin	Laccase from *Bacillus ligniniphilus* L1	37	24 h	*β*-*O*-4, *β*-5, *β*-*β*, 4-*O*-5, 5-5	Vanillyl alcohol, guaiacol, cetosyringenin	S17

	Corn stalk	Laccase from *Bacillus ligniniphilus* L1	37	24 h	*β-O*-4, *β*-5, *β*-*β,* 4-*O*-5, 5-5	Coniferyl aldehyde, syringaldehyde, 4-hydroxyphenylacetic acid, benzoic acid	S17
	5-5' Linkages lignin model compounds	Versatile peroxidase from *Physisporinus vitreus*	30	48 h	5-5'	Vanillic alcohol, vanillic aldehyde, and vanillic acid	S18
Combinatorial fractionation	Corn stover	Combinatorial chemical and mixed-enzyme treatments	/	/	*β-O*-4 and *β*-5	*p*-Coumaric acid, ferulic acid	S19

Table S2 Key enzymes and regulatory strategies in biological lignin valorization

Catalysis reaction	Enzymes	Gene origin	Classification	Coenzymes	Substrates	Products	Regulatory strategies	Regulation-related enzymes	Refs
O-demethylation	VanAB	*P. putida* KT2440	Rieske	FMN, NAD(P)H	Vanillic acid	Protocatechuic acid	Formaldehyde detoxification	HPS, PHI	S20
	VanAB	*Pseudomonas* sp. HR199	Rieske	FMN, NAD(P)H	Vanillic acid	Protocatechuic acid	NADH regeneration Formaldehyde detoxification	FADH, FDH	S21
	LigXaXcXd	*Sphingobium* sp. SYK-6	Rieske	FAD, NADH	5,5′-Dehydrovanillic acid	2,2′,3-Trihydroxy-3′-methoxy-5,5′-dicarboxybiphenyl	/	/	S22
	DesA	*Sphingobium* sp. SYK-6	THF-dependent demethylase	THF	Syringic acid	3-O-Methylgallic acid	THF regeneration	MetF, LigH	S23
	LigM	*Sphingobium* sp. SYK-6	THF-dependent demethylase	THF	Vanillic acid; 3-*O*-Methylgallic acid	Protocatechuic acid; Gallic acid	THF regeneration	MetF, LigH	S23
	GcoAB	*Amycolatopsis* sp. ATCC 39116	Cytochrome P450 enzyme	FAD, NADH	Guaiacol; Vanillin	Catechol; Protocatechualdehyde;	/	/	S24
Hydroxylation	PobA	*P. putida* KT2440	One-component FMO	FAD, NADPH	4-Hydroxybenzoic acid	Protocatechuic acid	Increasing cofactor availability	PraI	S25
	HpaBC	*E. coli*	Two-	FAD,	Tyrosine; Tyrosol	Levodopa;	$FADH_2$	LAAD	S26

			component FMO	NADH		Hydroxytyrosol	regeneration		
	HpaBC	HpaB from *P. aeruginosa* HpaC from *S. enterica*	Two-component FMO	FAD, NADH	*p*-Coumaric acid; 4-Hydroxybenzaldehyde;	Caffeic acid; Protocatechuic aldehyde	Enzyme fusion	/	[S27]
Decarboxylation	GS_120062	*G. subvermispora*	Oxidative decarboxylase	NAD(P)H	4-Hydroxybenzoic acid; Protocatechuic acid	Hydroquinone; 1,2,4-Benzenetriol	/	/	[S28]
	GS_90429	*G. subvermispora*	Oxidative decarboxylase	NAD(P)H	4-Hydroxybenzoic acid; Protocatechuic acid	Hydroquinone; 1,2,4-Benzenetriol	/	/	[S28]
	TV_32834	*T. versicolor*	Oxidative decarboxylase	NAD(P)H	Protocatechuic acid	1,2,4-Benzenetriol	/	/	[S28]
	FDC	*S. cerevisiae*	UbiD-type	PrFMN	*p*-Coumaric acid; Ferulic acid; Cinnamic acid	4-Vinylphenol; 4-Vinylguaiacol; Styrene	Co-expression of related proteins	PAD	[S29]
	AroY	*E. cloacae*	UbiD-type	PrFMN	Protocatechuic acid	Catechol	Co-expression of related proteins	EcdB; EcdD	[S30]
	LigW	*Sphingobium* sp. SYK-6	Amidohydrolase superfamily	Mn^{2+}	5-Carboxyvanillic acid	Vanillic acid	/	/	[S31]

Table S3 Examples of regulation of metabolic networks for valorizing lignin into high-value products

Classification	Products	Host strains	Substrates	Functional modules for regulation	Engineering strategies	Other strategies	Titers	Refs
Aromatic platform chemicals	Vanillin	*E. coli*	Ferulic acid	Vanillin synthesis module and CoA-regeneration module	Heterologous expression of *fcs* and *ech* from *Amycolatopsis* sp. HR104<br>Overexpression of *gltA*<br>Knockout of *icdA*	Application of XAD-2 resin for adsorption of toxic products	5.14 g/L	S32
	Vanillin	*S. cerevisiae*	Real hydrolysate of corn stover	Vanillin synthesis module, xylose metabolism module and tyrosine overproduction module	Knockout of *ADH*6, *ADH*7, *BDH*1, *BDH*2, *GRE*2, *HFD*1, *ARO*10, *PDC*5, *PHA*2, *FDC*1 and *PAD*1<br>Heterologous expression of *Pc*4CL, *Pp*Ech, *Pa*HpaB, *Se*HpaC, *At*COMT, *Nt*COMT, *Fj*TAL, *Ec*AROL,<br>Overexpression of *SAH1*, *MET6*, $MET13_{atsc}$, Aro3^{K222L}, Aro4^{K229L}, Aro7^{G141S}, XYL1, mXYL2, XKS1	Combined depolymerization strategies of lignin; Protein fusion expression	1.97 mM	S27
	Vanillin	*E. coli*	Ferulic acid	Product synthesis module and cell growth module	Combinatorial expression of PP3359 and *Pp*FCS	Two-Layer gene circuit	852 mg/L	S33
	p-Hydroxybenzaldehyde	*E. coli*	*p*-Coumaric acid	Product synthesis module and cell growth module	Combinatorial expression of PP3359 and *Pp*FCS	Two-Layer gene circuit	393 mg/L	S33
	Protocatechuic acid	*S. cerevisia*	Alkaline pretreatment	Protocatechuic acid synthesis	Knockout of *ADH6*, *ADH7*, *BDH2*, *FDC*, *MHT1*, *SAM4*	Fermentation optimization	810 mg/L	S34

	e	liquor	module and THF-regeneration module	Heterologous expression of *Pc*4CL, *Pp*ECH, *Pp*VDH, *Pp*PobA, *Pd*VanA, *Pd*VanB, *Sp*LigM, *Rd*Metf1<br>Overexpression of MET6			
Protocatechuic acid	*P. putida* KT2440	*p*-Coumaric acid and ferulic acid	Protocatechuic acid synthesis module and aromatic substrates transport module	Knockout of PcaGH<br>Combinatorial expression of PobA, Hcnk, *Bs*PadR and *Ac*VanAB	Auto-regulatory system; Promoter engineering; Fed-batch fermentation	12.7 g/L	S35
Vanillic acid	*E. coli*	Ferulic acid	Product synthesis module and cell growth module	Combinatorial expression of PP3359 and *Pp*FCS	Two-Layer gene circuit	880 mg/L	S33
Gallic acid	*Rhodococcus opacus* PD630	Base-depolymerized ammonia fiber explosion lignin	Gallic acid synthesis module and THF-regeneration module	Knockout of catechol 2,3-dioxygenase gene and protocatechuate 3,4-dioxygenase gene<br>Overexpression expression of PobA$^{Tyr386Phe/Thr295Ala}$, DesV, DesA,<br>Heterologous expression of *Ss*LigM, *Ss*MetF, *Ss*LigH	Protein engineering<br>Combined depolymerization strategies of lignin	2.469 mM	S23
Vanillylamine	*P. putida* KT2440	Ferulic acid	Vanillylamine synthesis module and L-alanine regeneration module	Knockout of PcaGH, AreA, VDH, ALD and BDH<br>Overexpression of FCS, ECH<br>Heterologous expression of *Cv*ATA, *Bs*AlaDH	Whole-cell bioconversion	0.473 mM	S36
Vanillylamine	*E. coli*	Ferulic acid	Vanillylamine	Heterologous expression of *Pp*FCS,	Increasing the ratio	19.22	S37

				synthesis module and L-alanine regeneration	*Pp*ECH, *Cv*TA, *Bs*AlaDH	of the NH4Cl donor	mM	
	Vanillylamine	*E. coli*	Vanillic acid	Vanillylamine synthesis module and L-alanine regeneration	Heterologous expression of *Sr*CAR, *Ni*PPTase, *Cv*TA, *Bs*AlaDH	Increasing the ratio of the NH4Cl donor	18.9 mM	[S37]
	Catechol	*E. coli*	Vanillin	Catechol synthesis module and aromatic substrates transport module	Combinatorial expression of *Rp*CouP, *Sp*LigV, *Sp*LigM, *Kp*AroY	Autoregulatory system	Approx. 8 mg/L	[S38]
Ring-opening products	Polyhydroxyalkanoates (PHAs)	*P. putida* KT2440	*p*-Coumaric acid	PHA accumulation module, fatty acid synthesis module, and *β*-oxidation module	Knockout of PhaZ, FadA, FadB Overexpression of PhaG, AlkK, PhaC	Fed-batch fermentation	953 mg/L	[S39]
		P. putida KT2440	Soluble lignin-rich stream	PHA accumulation module, fatty acid synthesis module, and *β*-oxidation module	Knockout of PhaZ, FadA, FadB Overexpression of PhaG, AlkK, PhaC	Combined depolymerization strategies of lignin	116 mg/L	[S39]
		P. putida KT2440	*p*-Coumaric acid	PHA accumulation	Knockout of PhaZ,AldB, FadA, FadB Overexpression of PhaG, PhaC, PhaJ4	Fed-batch fermentation	2.46 g/L	[S40]

			module and fatty acid *β*-oxidation				
	P. putida KT2440	Real lignin hydrolysate	PHA accumulation module and fatty acid *β*-oxidation module	Knockout of PhaZ,AldB, FadA, FadB Overexpression of PhaG, PhaC, PhaJ4	Fed-batch fermentation	0.93 g/L	S40
	P. putida A514	Lignin-enriched biorefinery residue	Lignin enzymatic depolymerization module, PHA accumulation module and fatty acid *β*-oxidation module	Heterologous expression of *Am*DyP2 Overexpression of VanAB, PhaJ4, PhaC1	Multi-omics analysis	Over 160 mg/L	S41
Polyhydroxybutyrate (PHB)	*Ralstonia eutropha* H16	Alkaline pretreated liquor of *Pinus massoniana*	*O*-demethylation module, Aromatic aldehyde metabolism module, and the mitigation of by-product inhibition module	Laboratory-evolved strains with the *gabD1* gene replaced with $pcaQ^{R145K}$ /P_{PCA}-*vanAB* and *fdhA* gene; the *iclA* gene replaced with $pcaQ^{R145K}$/P_{PCA}-*vdh*-*aldBI*-*aldBII* and the *iclB* gene replaced with $pcaQ^{R145K}$/P_{PCA}-*vanAB* and Ptac *fdhA* gene	Fed-batch fermentation, protein engineering, laboratory evolution and autoregulation	2.38 g/L	S42
cis-cis Muconic acid	*P. putida* KT2440	Real lignin hydrolysate	Muconic acid synthesis module, the S-type lignin utilization	Knockout of PcaHG, Overexpression of VanAB, ZwfA Heterologous expression of *Ec*AroY, EcdB	Fed-batch fermentation	9.6 mM	S43

			module, and the NADPH regeneration module				
	P. putida KT2440	Glycerol, *p*-coumaric acid, ferulic acid, and syringic acid	Muconic acid synthesis module, the S-type lignin utilization module, and the NADPH regeneration module	Knockout of PcaHG, Overexpression of VanAB, ZwfA Heterologous expression of *Ec*AroY, EcdB	Fed-batch fermentation	46.5 mM	S43
	P. putida KT2440	*p*-Coumaric acid	Muconic acid synthesis module and global carbon metabolism module	Knockout of CatRBC, Crc, PcaHG Heterologous expression of AroY, EcdBD	High pH fed-batch fermentation	50 g/L	S44
	P. putida KT2440	High-pH lignin streams	Muconic acid synthesis module and global carbon metabolism module	Knockout of CatRBC, Crc, PcaHG Heterologous expression of AroY, EcdBD	Fed-batch fermentation	3.7 g/L	S44
β-Ketoadipic acid	*P. putida* KT2440	*p*-Coumaric acid	*β*-Ketoadipic acid synthesis module and global carbon metabolism	Knockout of PobA, Crc, PcaHG Overexpression of VanAB, PacHG Heterologous expression of *Pa*$\text{PraI}_{\text{JJ-1b}}$	Fed-batch fermentation	44.5 g/L	S45

				module				
		P. putida KT2440	Alkaline pretreated liquor	*β*-Ketoadipic acid synthesis module and global carbon metabolism module	Knockout of PobA, Crc, PcaHG, PcaIJ, LvaE<br>Overexpression of VanAB, PacHG<br>Heterologous expression of *Pa*Pra$I_{JJ\text{-}1b}$	Fed-batch fermentation	25 g/L	S45
	2-Pyrone-4,6-dicarboxylic acid	*P. putida* KT2440	Protocatechuic acid	2-Pyrone-4,6-dicarboxylic acid synthesis module and aromatic substrates transport module	Knockout of PcaG<br>Heterologous expression of *Sp*LigAB<br>Overexpression of PcaK, PobA	Resting cells	0.58 g/L/h	S46
Natural products	Naringenin	*E. coli*	*p*-Coumaric acid	Flavonoids synthesis module and Malonate assimilation module	Heterologous expression of *Rt*MatB, *Rt*MatC, 4CL, CHS, CHI	Shake flask	155 mg/L	S47
	Naringenin	*E. coli*	*p*-Coumaric acid	Flavonoids synthesis module and malonyl CoA overproduction module	Knockout of SdhA, AdhE, BrnQ, CitE<br>Heterologous expression of CHI, CHS, 4CL2, ACC, BPL	Developed a cipher of evolutionary design to predict gene targets	215 mg/L	S48
	Homoeriodictyol	*S. cerevisiae*	*p*-Coumaric acid	Homoeriodictyol synthesis module, CoA supply module and SAM	Heterologous expression of *Pc*4CL, *Ms*CHI, *Ph*CHS, *Md*ECR, *At*ATR1, *At*F3'H, *Os*ROMT<br>Overexpression of ACC1, MET6, SAH1,	Protein engineering	3.2 mM	S49

				regeneration module	ADO1, MET13, Mthfr,<br>Knockout of CIT2, FDC1, Tsc13,			
	Kaempferol	*S. cerevisiae*	*p*-Coumaric acid and Glucose	Kaempferol synthesis module and Acetyl-CoA overproduction module	Heterologous expression of *Eb*4CL, *Eb*CHS, *Eb*CHI,*Eb*PAL, *Eb*C4H, *At*F3H, *Pd*FLS<br>Overexpression of *ADH2*, *ALD6*, *ACS*^{SE}, *ACC1*	Fed-batch fermentation	66.29 mg/L	S50

Table S4 Genome-scale metabolic models of ligninolytic microbes

Strains	Models	Metabolites	Genes	Reactions	Sources of data	Reconstruction	Aromatic compound metabolic pathways/enzymes	Refs
Pseudomonas putida KT2440	*i*JN746	911	746 (14%) [a]	950	TIGR, KEGG, PSEUDOCYC, SYSTOMONAS	Constraint-based reconstruction	*β*-Ketoadipate pathway, homogentisate pathway, phenylacetate pathway, gallate pathways and toluene and xylene degradation pathway	[S51]
	*i*JP815	888	815 (15%) [a]	877	The *Pseudomonas* Genome Database, NCBI, KEGG, BRENDA	Constraint-based reconstruction	Benzoate degradation via hydroxylation and benzoate degradation via CoA ligation	[S52]
	PpuMBEL1071	1044	900 (16.6%) [a]	1071	Genome annotation, CMR, KEGG, UMBBD, the *Pseudomonas* Genome Database, BioSilico, TransportDB	Constraint-based reconstruction	*β*-Ketoadipate pathway and homogentisate pathway	[S53]
	*i*JP962	992	962 (17%) [a]	1070	*Pseudomonas* Genome Database V2	Metabolic network reconciliation	Naphthalene degradation pathway, anthracene degradation pathway, phenylalanine degradation pathway and benzoate degradation pathway	[S54]
	*i*EB1050	1122	1050 (19%) [a]	1256	RFAM, UniProtKB, COG, HAMAP, FIGfam	MicroScope automatic reconstruction	CatA/A1, CatB, CatC,CatR, PcaHG,PcaIJ,PcaBCD, PcaP, PcaR, PcaK ,PcaF, GalABCD,GalT, GalP, BenABCDE, BenK, BenR, Fcs,Ech,	[S55]

							Vdh,VanAB, PobA, CalAB	
	PpuQY1140	1104	1140 (21%) [a]	1171	NCBI, KEGG, MetaCyc	Pathway-Consensus approach	PP_2518, PP_2513, PP_2515, PP_2514, PP_3357, PP_3358, PP_3356, PP_5120	S56
	*i*JN1462	2155	1462 (27%) [a]	2929	Genome annotation	Iterative tri-dimensional expansion	PcaK, 3-oxoadipatesuccinyl-CoA transferase and 3-oxoadipyl-CoA thiolase	S57
Rhodococcus opacus PD630	iGR1773	1956	1773	3025	Genome annotation, transcript data from the National Center for Bioinformatics Sequence Read Archive, , BiGG and KEGG	CarveMe automatic reconstruction	Phenol degradation pathway	S58
Novosphingobium aromaticivorans	iNovo479	604	479	645	KEGG annotation data for *N. aromaticivorans* DSM 12444, Integrated Microbial Genomes Database,	Constraints based on S-type aromatic metabolism	Vanillic acid demethylation pathway, syringic acid demethylation pathway, guaiacylglycerol-*β*-guaiacyl ether degradation pathway	S59
Pluralibacter gergoviae	*i*LL1097	1771	/	1886	Genome sequence for *P. gergoviae*, GenBank, UniProt , BiGG, KEGG and MetaCyc	Model Seed automatic reconstruction	DyP,AdhAB, Cat, Dld, Gox, Gpx, MdlC, NuoE,NuoF,NuoG, Sod, TrxB, CatABC, BenABCD, PobA, PcaHG,PcaIJ,PcaBCD, HpaBCG	S60
Rhodosporidium toruloides	/	2051	1141	2398	OrthoMCL, BiGG	COBRApy was used for modeling of the	Long-chain acyl-CoA synthetase, enoyl-CoA hydratase/isomerase, peroxisomal dehydratase, 3-	S61

		reconstructed metabolic network	oxoacyl-(acyl-carrier protein) reductase, 3-oxoacyl CoA thiolase, alpha/beta hydrolase, monooxygenase, lactonase, carboxymuconolactone decarboxylase, 3-oxoacid CoA-transferase, acetyl-CoA acyltransferase, mitochondrial 2-oxodicarboxylate transporter, aspartate aminotransferase, vanillin dehydrogenase, aldehyde dehydrogenase, Phenylalanine/tyrosine ammonia-lyase, cytochrome P450

a: % of genes represented

References

S1. Martínez P. M., Punt A. M., Kabel M. A., et al. (2016). Deconstruction of lignin linked *p*-coumarates, ferulates and xylan by NaOH enhances the enzymatic conversion of glucan. *Bioresour. Technol.* **216**:44 – 51. DOI:10.1016/j.biortech.2016.05.040

S2. Liu Z.H., Olson M. L., Shinde S., et al. (2017). Synergistic maximization of the carbohydrate output and lignin processability by combinatorial pretreatment. *Green Chem.* **19**:4939 – 4955. DOI:10.1039/c7gc02057k

S3. Zhang L., Yan L., Wang Z., et al. (2015). Characterization of lignin derived from water-only and dilute acid flowthrough pretreatment of poplar wood at elevated temperatures. *Biotechnol. Biofuels* **8**:1 – 14. DOI:10.1186/s13068-015-0377-x

S4. Duan D., Ruan R., Wang Y., et al. (2018). Microwave-assisted acid pretreatment of alkali lignin: Effect on characteristics and pyrolysis behavior. *Bioresour. Technol.* **251**:57 – 62. DOI:10.1016/j.biortech.2017.12.022

S5. da Costa Sousa L., Foston M., Bokade V., et al. (2016). Isolation and characterization of new lignin streams derived from extractive-ammonia (EA) pretreatment. *Green Chem.* **18**:4205 – 4215. DOI:10.1039/C6GC00298F

S6. Li D., Long L. and Ding S. (2020). Alkaline organosolv pretreatment of different sorghum stem parts for enhancing the total reducing sugar yields and *p*-coumaric acid release. *Biotechnol. Biofuels* **13**:106. DOI:10.1186/s13068-020-01746-4

S7. Moniz P., Serralheiro C., Matos C. T., et al. (2018). Membrane separation and characterisation of lignin and its derived products obtained by a mild ethanol organosolv treatment of rice straw. *Process Biochem.* **65**:136 – 145. DOI:10.1016/j.procbio.2017.11.012

S8. Rahimi A., Ulbrich A., Coon J. J., et al. (2014). Formic-acid-induced depolymerization of oxidized lignin to aromatics. *Nature* **515**:249 – 252. DOI:10.1038/nature13867

S9. Luo H., Wang L., Shang S., et al. (2020). Cobalt nanoparticles-catalyzed widely applicable successive C-C bond cleavage in alcohols to access esters. *Angew. Chem., Int. Ed. Engl.* **59**:19268 – 19274. DOI:10.1002/anie.202008261

S10. Ge J., Wang G., Sui W., et al. (2023). Highly efficient metal-acid synergetic catalytic fractionation of lignocellulose under mild conditions over lignin-coordinated N-anchoring Co single-atom catalyst. *Chem. Eng. J.* **462**:142109. DOI:10.1016/j.cej.2023.142109

S11. Kim J.Y., Park S. Y., Choi I.G., et al. (2018). Evaluation of Ru_xNi_{1-x}/SBA-15 catalysts for depolymerization features of lignin macromolecule into monomeric phenols. *Chem. Eng. J.* **336**:640 – 648. DOI:10.1016/j.cej.2017.11.118

S12. Zeng J., Mills M. J., Simmons B. A., et al. (2017). Understanding factors controlling depolymerization and polymerization in catalytic degradation of *β*-ether linked model lignin compounds by versatile peroxidase. *Green Chem.* **19**:2145 – 2154. DOI:10.1039/C6GC03379B

S13. Pham L. T. M., Seo H., Kim K.J., et al. (2018). In silico-designed lignin peroxidase from *Phanerochaete chrysosporium* shows enhanced acid stability for depolymerization of lignin. *Biotechnol. Biofuels* **11**:1 – 13. DOI:10.1186/s13068-018-1324-4

S14. Khan S. I., Zada N. S., Sahinkaya M., et al. (2021). Cloning, expression and biochemical characterization of lignin-degrading DyP-type peroxidase from *Bacillus* sp. Strain BL5. *Enzyme Microb. Technol.* **151**:109917. DOI:10.1016/j.enzmictec.2021.109917

S15. Kumar V, Chandra D, Thakur V, et al. (2023). Depolymerization of lignin using laccase from

Bacillus sp. PCH94 for production of valuable chemicals: a sustainable approach for lignin valorization. *Int. J. Biol. Macromol.* **234**:123601. DOI:10.1016/j.ijbiomac.2023.123601

S16. Cui T., Yuan B., Guo H., et al. (2021). Enhanced lignin biodegradation by consortium of white rot fungi: microbial synergistic effects and product mapping. *Biotechnol. Biofuels* **14**:1 – 11. DOI:10.1186/s13068-021-02011-y

S17. Zhu D., Liang N., Zhang R., et al. (2020). Insight into depolymerization mechanism of bacterial laccase for lignin. *ACS Sustainable Chem. Eng.* **8**:12920 – 12933. DOI:10.1021/acssuschemeng.0c03457

S18. Kong W., Fu X., Wang L., et al. (2017). A novel and efficient fungal delignification strategy based on versatile peroxidase for lignocellulose bioconversion. *Biotechnol. Biofuels* **10**:218. DOI:10.1186/s13068-017-0906-x

S19. Liu Z.H., Shinde S., Xie S., et al. (2019). Cooperative valorization of lignin and residual sugar to polyhydroxyalkanoate (PHA) for enhanced yield and carbon utilization in biorefineries. *Sustainable Energy Fuels* **3**:2024 – 2037. DOI:10.1039/c9se00021f

S20. Nguyen L. T., Tran M. H. and Lee E. Y. (2021). Co-upgrading of ethanol-assisted depolymerized lignin: A new biological lignin valorization approach for the production of protocatechuic acid and polyhydroxyalkanoic acid. *Bioresour. Technol.* **338**:125563. DOI:10.1016/j.biortech.2021.125563

S21. Lanfranchi E., Trajkovic M., Barta K., et al. (2019). Exploring the selective demethylation of aryl methyl ethers with a *Pseudomonas* rieske monooxygenase. *Chembiochem* **20**:118 – 125. DOI:10.1002/cbic.201800594

S22. Yoshikata T., Suzuki K., Kamimura N., et al. (2014). Three-component *O*-demethylase system essential for catabolism of a lignin-derived biphenyl compound in *Sphingobium* sp. strain SYK-6. *Appl. Environ. Microbiol.* **80**:7142 – 7153. DOI:10.1128/AEM.02236-14

S23. Cai C., Xu Z., Zhou H., et al. (2021). Valorization of lignin components into gallate by integrated biological hydroxylation, O-demethylation, and aryl side-chain oxidation. *science advance* **7**:eabg4585

S24. Mallinson S. J. B., Machovina M. M., Silveira R. L., et al. (2018). A promiscuous cytochrome P450 aromatic *O*-demethylase for lignin bioconversion. *Nat. Commun.* **9**:2487. DOI:10.1038/s41467-018-04878-2

S25. Kuatsjah E., Johnson C. W., Salvachua D., et al. (2022). Debottlenecking 4-hydroxybenzoate hydroxylation in *Pseudomonas putida* KT2440 improves muconate productivity from *p*-coumarate. *Metab. Eng.* **70**:31 – 42. DOI:10.1016/j.ymben.2021.12.010

S26. Wang H., Wang L., Chen J., et al. (2023). Promoting $FADH_2$ regeneration of hydroxylation for high-level production of hydroxytyrosol from glycerol in *Escherichia coli*. *J. Agric. Food Chem.* **71**:16681 – 16690. DOI:10.1021/acs.jafc.3c05477

S27. Xin X., Zhang R. K., Liu S. C., et al. (2024). Engineering yeast to convert lignocellulose into vanillin. *Chem. Eng. J.* **485**:149815. DOI:10.1016/j.cej.2024.149815

S28. Del Cerro C., Erickson E., Dong T., et al. (2021). Intracellular pathways for lignin catabolism in white-rot fungi. *Proc. Natl. Acad. Sci. U. S. A.* **118**:e2017381118. DOI:10.1073/pnas.2017381118

S29. Richard P., Viljanen K. and Penttila M. (2015). Overexpression of PAD1 and FDC1 results in significant cinnamic acid decarboxylase activity in *Saccharomyces cerevisiae*. *AMB Express.* **5**:12. DOI:10.1186/s13568-015-0103-x

S30. Johnson C. W., Salvachua D., Khanna P., et al. (2016). Enhancing muconic acid production from glucose and lignin-derived aromatic compounds via increased protocatechuate decarboxylase activity. *Metab. Eng. Commun.* **3**:111–119. DOI:10.1016/j.meteno.2016.04.002

S31. Peng X., Masai E., Kasai D., et al. (2005). A second 5-carboxyvanillate decarboxylase gene, ligW2, is important for lignin-related biphenyl catabolism in *Sphingomonas paucimobilis* SYK-6. *Appl. Environ. Microbiol.* **71**:5014–5021. DOI:10.1128/AEM.71.9.5014-5021.2005

S32. Lee E. G., Yoon S. H., Das A., et al. (2008). Directing vanillin production from ferulic acid by increased acetyl-CoA consumption in recombinant *Escherichia coli*. *Biotechnol. Bioeng.* **102**:200–208. DOI:10.1002/bit.22040

S33. Lo T. M., Chng S. H., Teo W. S., et al. (2016). A two-layer gene circuit for decoupling cell growth from metabolite production. *Cell Syst.* **3**:133–143. DOI:10.1016/j.cels.2016.07.012

S34. Zhang R. K., Tan Y. S., Cui Y. Z., et al. (2021). Lignin valorization for protocatechuic acid production in engineered *Saccharomyces cerevisiae*. *Green Chem.* **23**:6515–6526. DOI:10.1039/d1gc01442k

S35. Li J., Yue C., Wei W., et al. (2022). Construction of a *p*-coumaric and ferulic acid auto-regulatory system in *Pseudomonas putida* KT2440 for protocatechuate production from lignin-derived aromatics. *Bioresour. Technol.* **344**:126221. DOI:10.1016/j.biortech.2021.126221

S36. Manfrao-Netto J. H. C., Lund F., Muratovska N., et al. (2021). Metabolic engineering of *Pseudomonas putida* for production of vanillylamine from lignin-derived substrates. *Microb. Biotechnol.* **14**:2448–2462. DOI:10.1111/1751-7915.13764

S37. Fu B., Xiao G., Zhu Y., et al. (2021). Renewable vanillylamine synthesis from lignin-derived feedstocks. *ACS Agric. Sci. Technol.* **1**:566–571. DOI:10.1021/acsagscitech.1c00174

S38. Wu W., Liu F. and Singh S. (2018). Toward engineering *E. coli* with an autoregulatory system for lignin valorization. *Proc. Natl. Acad. Sci. U. S. A.* **115**:2970–2975. DOI:10.1073/pnas.1720129115

S39. Salvachua D., Rydzak T., Auwae R., et al. (2020). Metabolic engineering of *Pseudomonas putida* for increased polyhydroxyalkanoate production from lignin. *Microb. Biotechnol.* **13**:290–298. DOI:10.1111/1751-7915.13481

S40. Wang C., Liu R. Y., Xu T., et al. (2024). Valorizing lignin and coprecursors into homogeneous polyhydroxyalkanoates by engineered *Pseudomonas putida*. *ACS Sustainable Chem. Eng.* **12**:8402–8414. DOI:10.1021/acssuschemeng.4c01184

S41. Lin L., Cheng Y. B., Pu Y. Q., et al. (2016). Systems biology-guided biodesign of consolidated lignin conversion. *Green Chem.* **18**:5536–5547. DOI:10.1039/c6gc01131d

S42. Zhao Y., Xue L., Huang Z., et al. (2024). Lignin valorization to bioplastics with an aromatic hub metabolite-based autoregulation system. *Nat. Commun.* **15**:9288. DOI:10.1038/s41467-024-53609-3

S43. Liu H., Tao X., Ntakirutimana S., et al. (2024). Engineering *Pseudomonas putida* for lignin bioconversion into *cis-cis* muconic acid. *Chem. Eng. J.* **495**:153375. DOI:10.1016/j.cej.2024.153375

S44. Salvachúa D., Johnson C. W., Singer C. A., et al. (2018). Bioprocess development for muconic acid production from aromatic compounds and lignin. *Green Chem.* **20**:5007–5019. DOI:10.1039/c8gc02519c

S45. Werner A. Z., Cordell W. T., Lahive C. W., et al. (2023). Lignin conversion to *β*-ketoadipic acid by *Pseudomonas putida* via metabolic engineering and bioprocess development. *Sci. Adv.*

9:eadj0053. DOI: 10.1126/sciadv.adj0053

S46. Gómez-Álvarez H., Iturbe P., Rivero-Buceta V., et al. (2022). Bioconversion of lignin-derived aromatics into the building block pyridine 2, 4-dicarboxylic acid by engineering recombinant *Pseudomonas putida* strains. *Bioresour. Technol.* **346**:126638. DOI:10.1016/j.biortech.2021.126638

S47. Leonard E., Yan Y., Fowler Z. L., et al. (2008). Strain improvement of recombinant *Escherichia coli* for efficient production of plant flavonoids. *Mol. Pharmaceutics* **5**:257 – 265. DOI:10.1021/mp7001472

S48. Fowler Z. L., Gikandi W. W. and Koffas M. A. (2009). Increased malonyl coenzyme A biosynthesis by tuning the *Escherichia coli* metabolic network and its application to flavanone production. *Appl. Environ. Microbiol.* **75**:5831 – 5839. DOI:10.1128/AEM.00270-09

S49. Zhu S. Y., Liu S. C., Zhang C. X., et al. (2024). Pathway and enzyme engineering for the bioconversion of lignin derivatives into homoeriodictyol in *Saccharomyces cerevisiae*. *Green Chem.* 26:5260 – 5272. DOI:10.1039/d4gc00183d

S50. Duan L., Ding W., Liu X., et al. (2017). Biosynthesis and engineering of kaempferol in *Saccharomyces cerevisiae*. *Microb. Cell. Fact.* **16**:165. DOI:10.1186/s12934-017-0774-x

S51. Nogales J., Palsson B. O. and Thiele I. (2008). A genome-scale metabolic reconstruction of *Pseudomonas putida* KT2440: *i*JN746 as a cell factory. *BMC Syst. Biol.* **2**:79. DOI:10.1186/1752-0509-2-79

S52. Puchalka J., Oberhardt M. A., Godinho M., et al. (2008). Genome-scale reconstruction and analysis of the *Pseudomonas putida* KT2440 metabolic network facilitates applications in biotechnology. *PLoS Comput. Biol.* **4**:e1000210. DOI:10.1371/journal.pcbi.1000210

S53. Sohn S. B., Kim T. Y., Park J. M., et al. (2010). In silico genome-scale metabolic analysis of *Pseudomonas putida* KT2440 for polyhydroxyalkanoate synthesis, degradation of aromatics and anaerobic survival. *Biotechnol. J.* **5**:739 – 750. DOI:10.1002/biot.201000124

S54. Oberhardt M. A., Puchałka J., Martins dos Santos V. A., et al. (2011). Reconciliation of genome-scale metabolic reconstructions for comparative systems analysis. *PLoS Comput. Biol.* **7**:e1001116. DOI:10.1371/journal.pcbi.1001116

S55. Belda E., Van Heck R. G., José Lopez-Sanchez M., et al. (2016). The revisited genome of *Pseudomonas putida* KT2440 enlightens its value as a robust metabolic chassis. *Environ. Microbiol.* **18**:3403–3424. DOI:10.1111/1462-2920.13230

S56. Yuan Q., Huang T., Li P., et al. (2017). Pathway-consensus approach to metabolic network reconstruction for *Pseudomonas putida* KT2440 by systematic comparison of published models. *PloS one* **12**:e0169437. DOI:10.1371/journal.pone.0169437

S57. Nogales J., Mueller J., Gudmundsson S., et al. (2020). High-quality genome-scale metabolic modelling of *Pseudomonas putida* highlights its broad metabolic capabilities. *Environ. Microbiol.* **22**:255 – 269. DOI:10.1111/1462-2920.14843

S58. Roell G. W., Schenk C., Anthony W. E., et al. (2023). A high-quality genome-scale model for *Rhodococcus opacus* metabolism. *ACS Synth. Biol.* **12**:1632–1644. DOI:10.1021/acssynbio.2c00618

S59. Linz A. M., Ma Y., Scholz S., et al. (2022). iNovo479: Metabolic modeling provides a roadmap to optimize bioproduct yield from deconstructed lignin aromatics by *Novosphingobium aromaticivorans*. *Metabolites* **12**:366. DOI:10.3390/metabo12040366

S60. Peng Q., Zhao C., Wang X., et al. (2025). Modeling bacterial interactions uncovers the

importance of outliers in the coastal lignin-degrading consortium. *Nat. Commun.* **16**:639. DOI:10.1038/s41467-025-56012-8

S61. Kim J., Coradetti S. T., Kim Y. M., et al. (2020). Multi-omics driven metabolic network reconstruction and analysis of lignocellulosic carbon utilization in *Rhodosporidium toruloides*. *Front. Bioeng. Biotechnol.* **8**:612832. DOI:10.3389/fbioe.2020.612832
